# AI662270/GRP94 axis couples the unfolded protein response to mitochondrial dynamics during acute myocardial infarction

**DOI:** 10.1172/jci.insight.188904

**Published:** 2025-10-08

**Authors:** Suling Ding, Wen Liu, Zhiwei Zhang, Xiyang Yang, Dili Sun, Jianfu Zhu, Xiaowei Zhu, Shijun Wang, Mengshi Xie, Hongyu Shi, Junbo Ge, Xiangdong Yang

**Affiliations:** 1 Department of Cardiology, Shanghai Institute of Cardiovascular Diseases,; 2 NHC Key Laboratory of Ischemic Heart Diseases, Zhongshan Hospital, Fudan University, Shanghai, China.; 3 Department of Echocardiography,; 4 Reproductive Medicine Centre, and; 5 Department of Critical Care Medicine, Zhongshan Hospital, Fudan University, Shanghai, China.; 6 Wusong Hospital, Zhongshan Hospital, Fudan University, Shanghai, China.; 7 Department of Cardiology, The Third People’s Hospital of Huizhou, Huizhou, Guangdong, China.

**Keywords:** Cardiology, Cell biology, Cell stress, Hypoxia, Noncoding RNAs

## Abstract

The unfolded protein response (UPR), triggered by endoplasmic reticulum (ER) stress, comprises distinct pathways orchestrated by conserved molecular sensors. Although several of these components have been suggested to protect cardiomyocytes from ischemic injury, their precise functions and mechanisms remain elusive. In this study, we observed a marked increase in glucose-regulated protein 94 (GRP94) expression at the border zone of cardiac infarct in a mouse model. GRP94 overexpression ameliorated post-infarction myocardial damage and reduced infarct size. Conversely, GRP94 deficiency exacerbated myocardial dysfunction and infarct size. Mechanistically, GRP94 alleviated hypoxia-induced mitochondrial fragmentation, whereas its depletion exacerbated this fragmentation. Molecular investigations revealed that GRP94 specifically facilitated the cleavage of Opa1 into L-Opa1, but not S-Opa1. The study further elucidated that under hypoxic conditions, the binding shift of Yy1 from lncRNA Oip5os1 to AI662270 promoted Yy1’s binding on the GRP94 promoter, thereby enhancing GRP94 expression. AI662270 attenuated mitochondrial over-fragmentation and ischemic injury after myocardial infarction similarly to GRP94. Moreover, coimmunoprecipitation coupled with LC-MS/MS identified the interaction of GRP94 with Anxa2, which regulates Akt1 signaling to maintain L-Opa1 levels. Overall, these findings unveiled what we believe is a novel role for the AI662270/GRP94 axis in linking ER stress to mitochondrial dynamics regulation, proposing new therapeutic avenues for managing cardiovascular conditions through ER stress modulation.

## Abstract 2

LncRNA AI662270-GRP94 protects myocardial cells from ischemic injury by regulating mitochondrial dynamics, specifically maintaining L-Opa1 levels under hypoxic stress.

## Graphical abstract

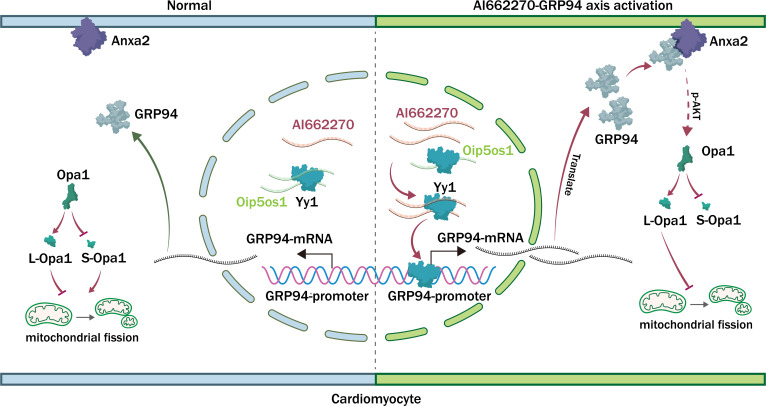

## Introduction

Acute myocardial infarction (AMI), distinguished by myocardial hypoxia and the ensuing demise of cardiomyocytes, stands as a prominent contributor to sudden fatalities on a global scale (1, 2). Furthermore, even in cases of survival, it can lead to the development of various medical conditions, including interstitial fibrosis, hypertension, atherosclerosis, and heart failure (3, 4). Early prevention of cardiomyocyte loss constitutes a therapeutic cornerstone in AMI management. Nonetheless, the advancement in the development of cardioprotective agents has yet to meet satisfactory standards, underscoring the pressing necessity to explore the molecular mechanisms controlling cardiomyocyte fate from a novel perspective.

The endoplasmic reticulum (ER) is a membranous organelle in which essential cellular processes, including protein processing, calcium homeostasis, and lipid biosynthesis, are orchestrated (5). Various cellular stressors, such as oxidative stress, ischemic injury, and disturbances in calcium homeostasis, can lead to the accumulation of unfolded proteins, a condition termed ER stress. ER stress activates the unfolded protein response (UPR) to alleviate stress and restore ER homeostasis, involving 3 ER sensors: PERK, ATF6, and IRE1 (6). ER stress and the ensuing UPR have been implicated in the pathogenesis of conditions such as MI (7), ischemia (8), dilated cardiomyopathy (9), atherosclerosis (10), and heart failure (11). While prolonged UPR activation has been reported to have deleterious effects (12), several UPR-related regulators, including ATF6 (12), XBP1 (13), and GRP78 (14), have been documented to confer cardioprotection during acute ischemia. Therefore, strategies aimed at selectively modulating specific components of the UPR hold promise for the development of novel therapeutic interventions to mitigate cardiomyocyte death in the early stages of cardiac diseases. Glucose-regulated protein 94 GRP94 (*Hsp90b1*), a UPR-regulated molecular chaperone, shows elevated myocardial expression in cardiovascular patients (15, 16); however, its precise role and underlying mechanisms remain to be fully elucidated.

Emerging evidence highlights mitochondria as membranous organelles crucial for energy production and cellular viability (17, 18). Acute stress and mitochondrial damage can lead to excessive fragmentation of the mitochondrial network, triggering cellular apoptosis (19, 20). Accumulating evidence has revealed that the ER and mitochondria share pathways that regulate cellular activity and cell death (21). Comprehending the molecular crosstalk between the mitochondrial dynamic machinery and ER stress response components could provide valuable insights into our understanding of diseases linked to mitochondrial dysfunction, particularly in cardiac physiology and pathology.

Long noncoding RNAs (lncRNAs), characterized by their length of more than 200 nucleotides, have been shown to play roles in various biological and pathological processes (22) by interacting directly with RNA, DNA, or proteins to remodel chromatin architecture or serving as scaffolds for microRNA sponges (23, 24). However, it remains unclear whether any specific lncRNAs are involved in the regulation of cardiac ER stress and UPR following AMI.

The present study reveals that GRP94 attenuates mitochondrial fragmentation and myocardial injury through interacting with Anxa2 to maintain the long isoform of Opa1 (L-Opa1) levels after AMI. We demonstrate that Yy1 takes part in activating GRP94 transcription. Furthermore, we observed that the shift of Yy1 binding with lncRNA Oip5os1 to AI662270 promoted its binding to the promoter region of GRP94, favoring the transcription of GRP94 under hypoxic conditions. The manipulation of AI662270 has the potential to regulate mitochondrial fragmentation and cardiac function through facilitating the GRP94/L-Opa1 pathway after AMI. Collectively, this study provides evidence that a AI662270/GRP94/Opa1 axis modulates cardiomyocyte ER stress response and mitochondrial crosstalk to protect the cardiac tissue from AMI.

## Results

### Elevated GRP94 expression confers cardioprotection during the early stages of MI.

**Figure 1:**
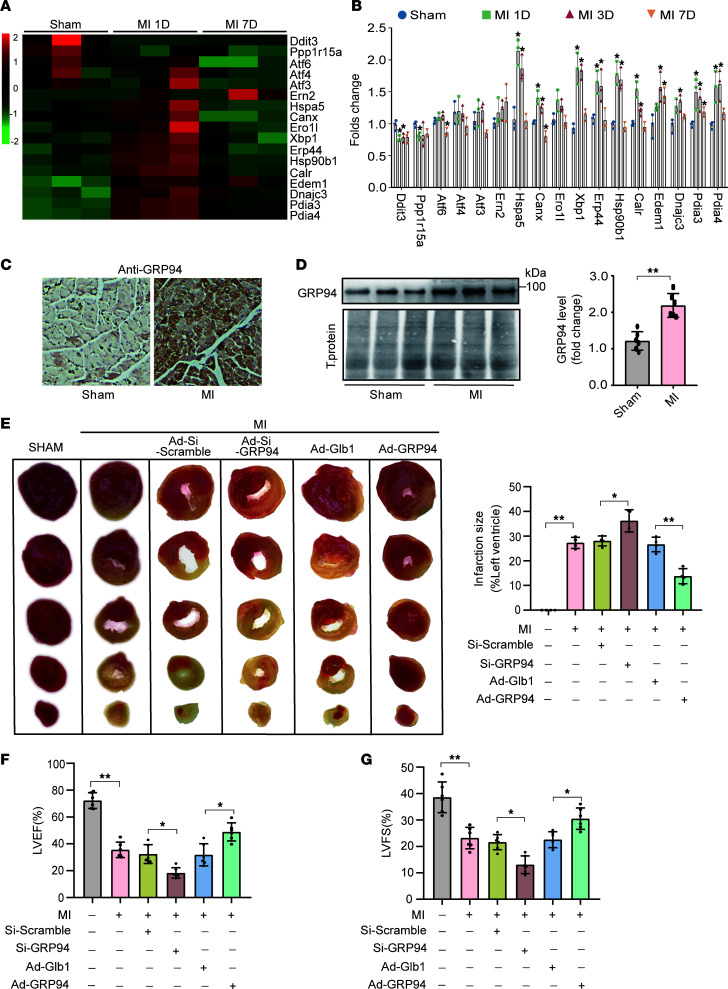
GRP94 attenuates cardiac dysfunction after MI. (**A**) Heatmap showing DEGs specific to ER stress. (**B**) qPCR validation of DEGs specific to ER stress revealed by RNA-seq. (**C**) Immunohistochemical GRP94 expression in peri-infarct myocardium. (**D**) Mice were subjected to MI surgery as described in Methods. GRP94 levels were detected by immunoblot (mean ± SD, *n* = 4). (**E**) GRP94 attenuates infarct sizes 1 day after MI. TTC staining on the left shows infarct size modulation by AAV2/9-GRP94 overexpression (Ad-GRP94) or siRNA knockdown (Si-GRP94) at 24 hours after MI. Right panel shows the percentage of infarct sizes. *n* = 4. (**F** and **G**) GRP94 markedly improves cardiac function after MI. Transthoracic echocardiographic analysis was performed at 1 week after surgery. *n* = 6. LVEF, left ventricular ejection fraction; LVFS, left ventricular fractional shortening. *P < 0.05; **P < 0.01 by unpaired, 2-tailed Student’s *t* test (**D**) or 1-way ANOVA with Tukey-Kramer post hoc analysis (**E**–**G**).

RNA-seq analysis of left ventricle myocardium from MI mouse models at 1 day and 7 days revealed the extensive upregulation of ER stress response genes at 1 day after MI, especially *Hspa5*, *Xbp1*, *Erp44*, and *Hsp90b1* (Figure 1A and Supplemental Table 1; supplemental material available online with this article; https://doi.org/10.1172/jci.insight.188904DS1). The results were confirmed by quantitative real-time PCR (qRT-PCR) (Figure 1B). Although the roles of *Hspa5*, *Xbp1*, and *Erp44* in cardiac pathophysiology are well characterized (25, 26), the pathophysiological role of *Hsp90b1* (GRP94) during ischemic myocardial remodeling remains elusive. Immunohistochemistry and immunoblotting confirmed the increased level of GRP94 in the infarct border zone at 1 day after MI (Figure 1, C and D). To delineate the distribution of GRP94 in cardiac tissue, we conducted immunofluorescent costaining analysis with cell-specific markers in myocardial sections. The results demonstrated predominant perinuclear localization of GRP94 in cardiomyocytes (α-actinin^+^), with more α-actinin^+^ cells showing strong GRP94 signal in the 1-day-post-MI group (Supplemental Figure 1A). In contrast, GRP94 signal intensity remained stable in both cardiac fibroblasts (vimentin^+^) and coronary endothelial cells (CD31^+^) across experimental groups (Supplemental Figure 1B). Adeno-associated virus 2/9 (AAV2/9) carrying cardiac troponin T (cTnT) promoter–driven small hairpin RNA targeting GRP94 (Si-GRP94) in vivo exacerbated MI size, while the in vivo administration of AAV9 carrying cTnT promoter–driven GRP94 overexpression (Ad-GRP94) markedly reduced infarct size at 1 day after MI (Supplemental Figure 1, C and D, and Figure 1E). Echocardiography studies revealed a reduced left ventricular ejection fraction (LVEF) (Figure 1F) and left ventricular fractional shortening (LVFS) (Figure 1G) in mice administered GRP94 siRNA adenoviruses 7 days after MI. In contrast, AAV9 overexpressing GRP94 markedly improved LVEF (Figure 1F) and LVFS (Figure 1G) in mice.

To further determine whether there is a dose-response relationship between GRP94 modulation and its cardioprotective effect, graded doses of GRP94-overexpressing AAV2/9 vectors were administered via intramyocardial injection in peri-infarct zones. Quantitative TUNEL analysis revealed a trend toward dose-dependent effects, with the higher dose showing a more pronounced reduction in cardiomyocyte apoptosis compared with the lower dose (Supplemental Figure 1, E and F). Cardiac functional parameters demonstrated dose-dependent improvement until reaching a therapeutic plateau (Supplemental Figure 1, G and H), with a 2-fold escalation (from 1 × 10¹¹ to 2 × 10¹¹ viral genomes) failed to produce additional significant improvement in cardiac function.

### GRP94 participates in the regulation of mitochondrial dynamics through mitigating Opa1 proteolytic cleavage to S-Opa1.

**Figure 2:**
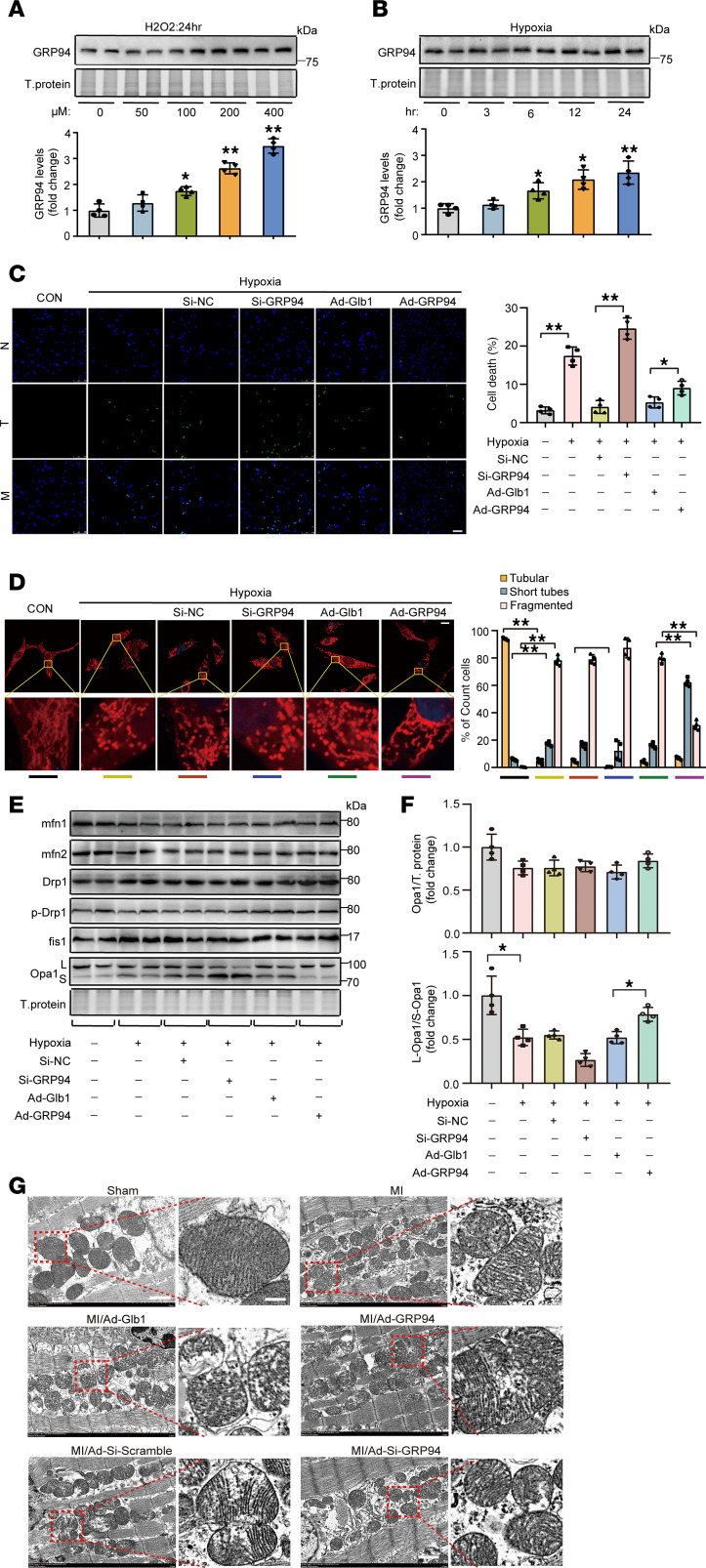
GRP94 attenuates cardiomyocyte apoptosis and mitochondrial fragmentation induced by hypoxia. (**A**) Cardiomyocytes were exposed to the indicated dose of H_2_O_2_. After 24 hours, cells were harvested for the analysis of GRP94 levels by immunoblot. *n* = 4. (**B**) Cardiomyocytes were cultivated under hypoxia for the indicated time. After 24 hours, cells were harvested for the analysis of GRP94 levels by immunoblot (*n* = 4). Data analyzed versus the untreated group. (**C**) GRP94 attenuates cells’ sensitivity to hypoxia-induced apoptosis. Cardiomyocytes were infected with adenoviruses overexpressing *GRP94* (Ad-GRP94) or *Glb1* (Ad-Glb1) at MOIs of 50, or transfected with 60 nM GRP94 siRNA (Si-GRP94) or siRNA scramble (Si-NC). After 36 hours, cells were cultivated for another 12 hours under hypoxic conditions. The TUNEL assay was utilized to detect apoptosis. Quantitative analysis of apoptosis is shown in right panel. CON, control. Scale bar: 50 μm. (**D**) GRP94 attenuates mitochondrial fission caused by hypoxia stress. Cardiomyocytes were treated as in **C**. Representative photos show mitochondrial fission (left). The cells were stained with MitoTracker, and then the ratios of cells with lamentous network, intermediate, or completely fragmented mitochondrial structures were calculated. At least 50 cells were counted from different optical fields of each sample (*n* = 4). CON, control. Scale bars: 20 μm. (**E** and **F**) GRP94 attenuates the cleavage of Opa1 into (S)-Opa1 caused by hypoxia stress. Cardiomyocytes were treated as in **C**. The levels of MFN1, MFN2, Opa1, DRP1, p-DRP1, and FIS1 were analyzed by immunoblot. *n* = 4. (**G**) GRP94 attenuated AMI-induced mitochondrial over-fragmentation. Adult male C57BL/6 mice (8–10 weeks old) were injected with AAV2/9 carrying *GRP94*, *Glb1*, *GRP94* siRNA, or siRNA scramble and subjected to permanent left anterior descending coronary artery ligation. The left panels are representative photos of TEM images of cardiac slices. Scale bars: 1 μm (left images) and 100 nm (enlarged images). *P < 0.05; **P < 0.01 by 1-way ANOVA with Tukey-Kramer post hoc analysis (**A**–**C**, **E**, and **F**) or 2-way ANOVA followed by Bonferroni’s multiple-comparison test (**D**).

The in vivo results established the cardioprotective efficacy of GRP94 against cardiac injury after AMI. To explore the detailed function of GRP94 in cardiomyocytes, the expression pattern of GRP94 protein was detected in cardiomyocytes under oxidative stress or hypoxic conditions. Immunoblotting revealed dose-dependent GRP94 upregulation with H_2_O_2_ treatment (Figure 2A and Supplemental Figure 2A), paralleled by acute hypoxia–induced GRP94 accumulation within 6 hours (Figure 2B).

Next, cardiomyocytes were infected with GRP94 siRNA or overexpressing adenoviruses to investigate their impact on cardiomyocyte death caused by hypoxia (Supplemental Figure 2B). CCK8 assay revealed that GRP94 silencing exacerbated hypoxia-induced viability loss, whereas overexpression preserved viability (Supplemental Figure 2C). TUNEL assay confirmed that GRP94 silencing intensified, while overexpression reduced, cardiomyocyte apoptosis (Figure 2C).

The dynamic alteration of mitochondria is an intracellular signal for the execution of apoptosis, and the close association of ER with mitochondria inspired us to determine whether GRP94 was involved in the regulation of mitochondrial dynamics. Mitochondrial tracker staining assays showed that GRP94 silencing intensified, while overexpression mitigated, mitochondrial fragmentation caused by hypoxia treatment in cardiomyocytes (Figure 2D).

Next, we explored whether there was any change in the levels of the components in the machinery mediating mitochondrial fission and fusion (27). The results of immunoblotting showed no changes in the levels of total Drp1, Opa1, fis1, Mfn1, and Mfn2, or Drp1-Ser616 phosphorylation (Figure 2E and Supplemental Figure 2, D–H). Strikingly, GRP94 silencing promoted Opa1 cleavage to the short isoform (S-Opa1), while overexpression preserved L-Opa1 (Figure 2, E and F), which was confirmed in the in vivo mouse model (Supplemental Figure 2, I and J).

The regulatory role of GRP94 in mitochondrial fission was also confirmed in an in vivo mouse model, with GRP94 knockdown decreasing but overexpression increasing the mean mitochondrial size in AMI cardiomyocytes (Figure 2G and Supplemental Figure 2K); however, neither manipulation altered inter-cristae width (Supplemental Figure 2K). These findings align with evidence that Opa1 proteolytic processing primarily governs mitochondrial fission/fusion dynamics rather than cristae maintenance (28, 29), suggesting that GRP94 contributes to mitochondrial homeostasis through Opa1-dependent mechanisms without directly modulating cristae architecture.

### Yy1 activates GRP94 transcription and expression.

**Figure 3:**
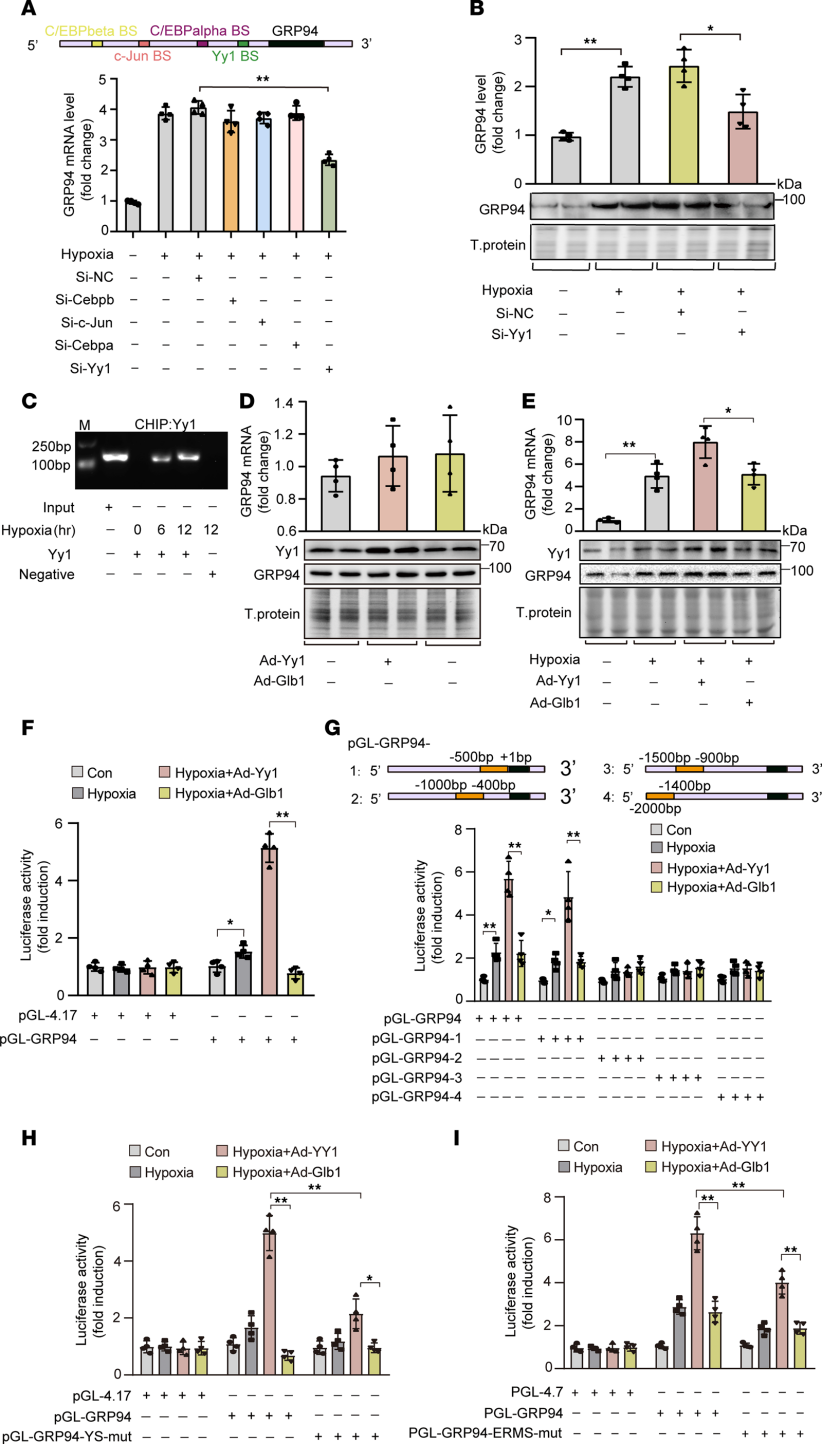
GRP94 is a transcriptional target of Yy1. (**A**) Hypoxia-induced GRP94 mRNA upregulation is suppressed by Yy1 knockdown. Mouse cardiomyocytes transfected with 40 nM siRNAs targeting *Cebpb*, *c-Jun*, *Cebpa*, *Yy1*, or scramble control (Si-NC) were exposed to hypoxia for 12 hours. GRP94 mRNA levels were quantified by qRT-PCR (*n* = 4). (**B**) Yy1 knockdown inhibits hypoxia-induced GRP94 protein expression. Cardiomyocytes transfected with Si-Yy1 or Si-NC were subjected to hypoxia. GRP94 protein was analyzed by immunoblot with total protein as loading control (*n* = 4). (**C**) Yy1 binds to the GRP94 promoter under hypoxia. ChIP assays were performed using Yy1 or β-actin (negative control) antibodies in cardiomyocytes exposed to hypoxia for the indicated durations. (**D** and **E**) Yy1 regulates GRP94 protein in a hypoxia-dependent manner. (**D**) GRP94 levels were unaffected by Yy1 overexpression (Ad-Yy1, MOI = 40) under normoxia. (**E**) Ad-Yy1 enhanced hypoxia-induced GRP94 expression (*n* = 4). (**F**–**H**) Yy1 activates GRP94 promoter via direct binding. (**F**) Luciferase reporter assays in MCM cells transfected with GRP94 promoter (pGL-GRP94) or empty vector (pGL-4.17), followed by Ad-*Yy1*/Ad-*Glb1* (MOI = 40) and hypoxia (*n* = 4). (**G**) The –500 to +1 bp promoter region mediates Yy1 responsiveness. (**H**) Mutation of Yy1-binding site (pGL-GRP94-YS-mut) abolishes Yy1-driven activation. (**I**) ER stress response element (ERMS) mutation (pGL-GRP94-ERMS-mut) attenuates Yy1-dependent promoter activity (*n* = 4). *P < 0.05; **P < 0.01 versus the indicated group (or vs. Si-NC in **A**) by 1-way ANOVA with Tukey-Kramer post hoc analysis (**A**, **B**, and **D**–**G**) or 2-way ANOVA with Bonferroni’s multiple-comparison test (**H** and **I**). All data are shown as mean ± SD.

To investigate the mechanisms underlying hypoxia-induced GRP94 protein upregulation in cardiomyocytes, we generated luciferase vectors containing either the promoter region or 3′UTR sequence of *GRP94* mRNA to transfect HL-1 cells. The activity of luciferase vectors containing the promoter region but not the 3′UTR region showed significant activation in response to hypoxia treatment (Supplemental Figure 3A). Consistently, *GRP94* mRNA levels showed marked upregulation in response to hypoxia (Supplemental Figure 3B). Analyzing the promoter region of mouse GRP94 using PROMO revealed that there are 9 transcription factors possessing ideal binding sites on them, including *Cebpb*, *Hes1*, *c-Fos*, *Cebpa*, *GR*, *c-Jun*, *Nf1*, *Hoxa5*, and *Yy1*. Among them, Cebpb, Cebpa, c-Jun, and Yy1 have been implicated in the modulation of ER stress response. Functional screening using siRNA-mediated knockdown revealed that Yy1 depletion specifically abolished hypoxia-triggered GRP94 induction at both mRNA (Figure 3A) and protein levels (Figure 3B). Immunofluorescent costaining revealed stable nuclear-restricted Yy1 expression in cardiomyocytes (actinin^+^), fibroblasts (vimentin^+^), and endothelial cells (CD31^+^) across myocardial compartments between Sham and 1 day after MI (Supplemental Figure 3C). The ChIP assay demonstrated that Yy1 is not bound to the GRP94 promoter under normoxic conditions in cardiomyocytes, but hypoxia treatment led to an increase in the association of Yy1 within the promoter of GRP94 (Figure 3C). Coherently, although hypoxia treatment slightly upregulated the total and nuclear level of Yy1 in cardiomyocytes (Supplemental Figure 3, D and E), Yy1 overexpression augmented the expression of GRP94 only under hypoxia treatment but not normoxic conditions (Figure 3, D and E, and Supplemental Figure 3, F and G). Luciferase reporter assays demonstrated that Yy1 overexpression potentiated hypoxia-responsive GRP94 promoter activation (Figure 3F). Segmenting the promoter region of GRP94 to construct luciferase vectors uncovered that the nucleotide sequence from –500 to +1 in the GRP94 promoter, which contains the Yy1 binding site at nucleotides –202 to –193, responded to hypoxia treatment and Yy1 overexpression (Figure 3G). Site-directed mutagenesis of this Yy1-binding element (Supplemental Figure 3, H and I) abolished Yy1-mediated promoter activation (Figure 3H). Notably, beside the Yy1-binding motif, the –500 to +1 bp GRP94 promoter region contains a conserved ER stress response element (ERSE, CCAAT[N9]CCACG). Given prior evidence of Yy1-ERSE interactions in gene regulation (30), we performed site-directed mutagenesis of the ERSE. This intervention partially abolished the ability of Yy1 to activate the GRP94 promoter (Figure 3I). Moreover, combinatorial mutations in the Yy1 binding site and ERSE site almost completely abrogated the effects of hypoxia treatment and Yy1 on the activation of the GRP94 promoter (Supplemental Figure 3J), implying functional synergy between these 2 *cis* elements in hypoxic transcriptional regulation of GRP94.

### lncRNA AI662270 favors Yy1-mediated GRP94 transcription.

**Figure 4:**
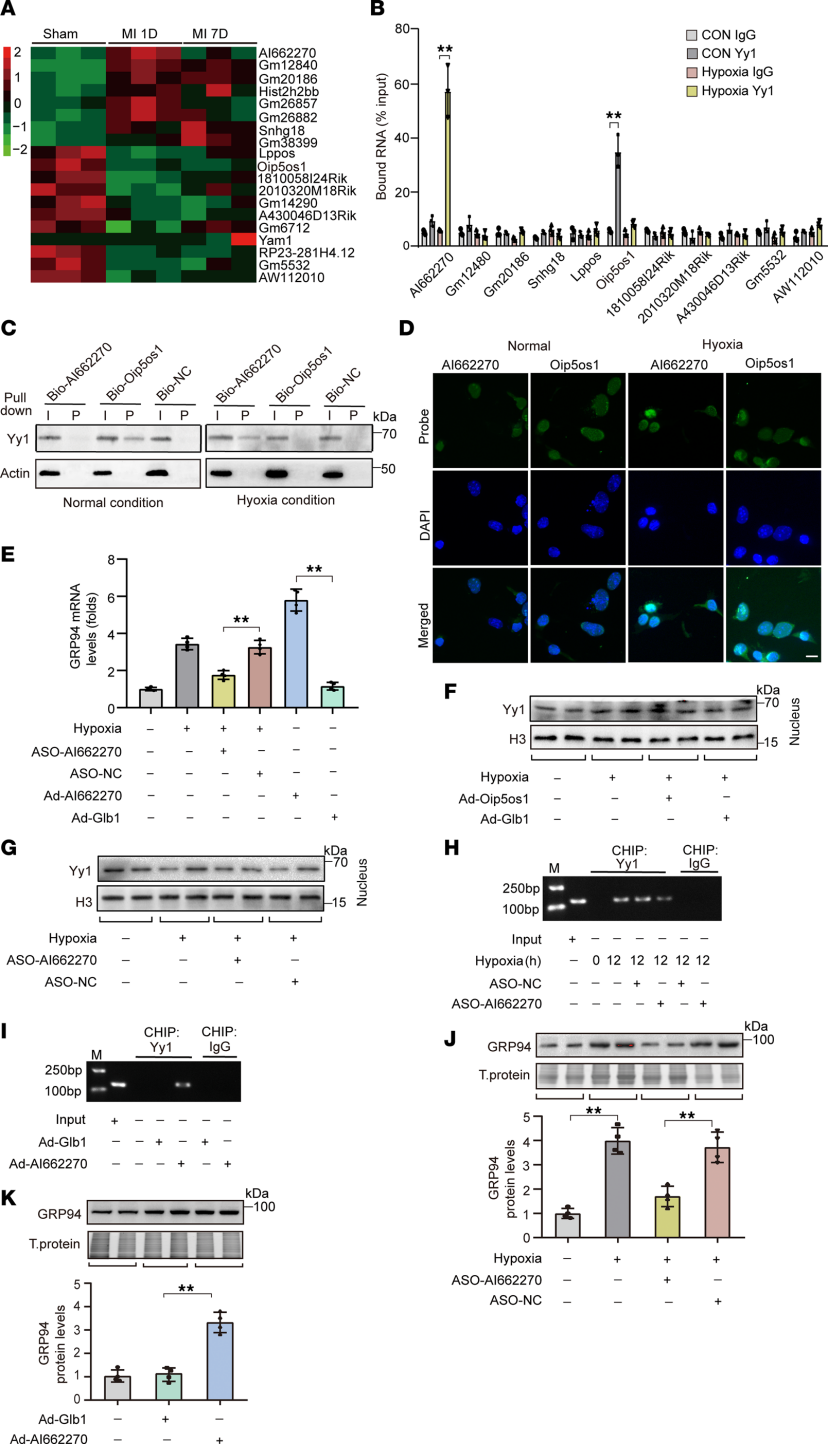
Lnc-AI662270 binds to Yy1 and facilitates Yy1-mediated GRP94 transcription. (**A**) Heatmap shows differentially expressed lncRNAs in the mouse model after MI. *n* = 3. (**B**) Hypoxia enhances Yy1-dependent enrichment of Lnc-AI662270. Cardiomyocytes infected with Ad-Glb1 or Ad-Yy1 (MOI = 40) for 24 hours were exposed to hypoxia (12 hours). RNA immunoprecipitation (RIP) using anti-Yy1 or IgG antibody, followed by qRT-PCR (*n* = 3). (**C**) Hypoxia redirects Yy1 binding from Lnc-Oip5os1 to Lnc-AI662270. RNA pull-down with biotinylated probes (Bio-AI662270, Bio-Oip5os1, or Bio-NC) in cardiomyocytes. Yy1 binding was analyzed by immunoblot (actin as loading control). (**D**) Subcellular localization of Lnc-AI662270 (cytoplasmic) and Lnc-Oip5os1 (nuclear) under hypoxia. Fluorescence in situ hybridization (FISH) in cardiomyocytes after 12-hour hypoxia. Scale bar: 20 μm. (**E**) Lnc-AI662270 regulates hypoxia-induced *GRP94* mRNA. Cardiomyocytes infected with Ad-AI662270 or Ad-Glb1 (MOI = 40), or transfected with 40 nM ASO-AI662270/ASO-NC for 36 hours, were exposed to hypoxia (12 hours). GRP94 mRNA levels analyzed by qRT-PCR (*n* = 4). (**F** and **G**) AI662270 or Oip5os1 does not alter nuclear Yy1 abundance. (**F**) Nuclear Yy1 levels in cells infected with Ad-Oip5os1 or Ad-Glb1 (MOI = 40). (**G**) Nuclear Yy1 in cells transfected with ASO-AI662270/ASO-NC. Cells were exposed to hypoxia (12 hours) prior to nuclear fractionation (*n* = 4). (**H** and **I**) Lnc-AI662270 modulates Yy1 occupancy on the GRP94 promoter. (**H**) ASO-AI662270 reduces Yy1 binding to the GRP94 promoter. (**I**) Ad-AI662270 enhances Yy1-promoter interaction. ChIP-qPCR with anti-Yy1 or IgG after 36 hr treatment (*n* = 4). (**J** and **K**) Hypoxia-dependent GRP94 regulation by Lnc-AI662270. (**J**) ASO-AI662270 attenuates hypoxia-induced GRP94 protein. (**K**) Ad-AI662270 elevates GRP94 under hypoxia. Immunoblots quantified from 48 hours after transfection (*n* = 4). *P < 0.05; **P < 0.01 versus the indicated group by 1-way ANOVA with Tukey-Kramer post hoc analysis (**B**, **E**, **J**, and **K**). All data are shown as mean ± SD.

Given established roles of lncRNAs in modulating transcription factor activity (31), we interrogated potential Yy1-interacting lncRNAs regulating GRP94 in cardiomyocytes. The analysis of the above RNA-seq 1 day after MI uncovered 19 differentially expressed lncRNAs in mouse left ventricular myocardium, with a multiple of expression level change of less than 0.7 or more than 1.5 and *P* value of less than 0.05 (Figure 4A and Supplemental Table 2). We confirmed the change in levels of these lncRNAs in hypoxic cardiomyocytes using qRT-PCR and excluded those with extremely low expression (Ct < 35 cycles). Five lncRNAs were substantially decreased, while 4 lncRNAs were markedly upregulated upon hypoxia treatment (Supplemental Figure 4A). To delineate Yy1-associated lncRNAs, we performed RNA immunoprecipitation (RIP) experiments. Unexpectedly, the results showed that Yy1 was enriched with Oip5os1 under normal conditions but enriched with AI662270 instead under hypoxia treatment in cardiomyocytes (Figure 4B). To further validate their interaction, we generated biotinylated probes for Oip5os1 and AI662270 to be utilized for RNA pull-down assays. The results showed that Oip5os1 strongly bound with Yy1 only in normoxic cardiomyocytes, and AI662270 bound with Yy1 in hypoxic cardiomyocytes (Figure 4C), which suggested a switch of the interaction of Yy1 with Oip5os1 to AI66227 from normal conditions to hypoxia treatment. The lncLocator algorithm (http://www.csbio.sjtu.edu.cn/bioinf/lncLocator/) predicted predominant nuclear localization of both Oip5os1 and AI662270 (Supplemental Figure 4B), which was subsequently validated by FISH assays (Figure 4D). Hypoxic exposure upregulated AI662270 expression and downregulated Oip5os1 (Supplemental Figure 4A). To determine whether the hypoxia-induced upregulation of AI662270 or downregulation of Oip5os1 contributes to Yy1-mediated regulation of GRP94 transcription, we reversed the change of their expression levels caused by hypoxia treatment (Supplemental Figure 4, C and D). We observed that overexpression or silencing of Oip5os1 failed to affect GRP94 mRNA levels significantly (Supplemental Figure 4E). However, suppressing AI662270 upregulation attenuated hypoxia-induced GRP94 expression, whereas AI662270 overexpression further elevated *GRP94* mRNA levels beyond those observed under hypoxia alone (Figure 4E). Neither AI662270 nor Oip5os1 affected the protein levels of Yy1 in total cells or nuclei (Figure 4, F and G, and Supplemental Figure 4, F and G). However, ChIP analysis revealed that AI662270 knockdown attenuated Yy1 binding to the GRP94 promoter under hypoxia (Figure 4H), whereas AI662270 overexpression enhanced this interaction (Figure 4I). Consistent with these findings, AI662270 depletion markedly suppressed hypoxia-induced GRP94 protein upregulation (Figure 4J), while its overexpression substantially elevated GRP94 expression beyond baseline hypoxic levels (Figure 4K). These findings revealed a potentially novel role for lncRNA AI662270 in facilitating Yy1 recruitment to the GRP94 promoter, thereby driving the transcription of GRP94.

### AI662270 is specifically induced by ER stress and has a similar effect on GRP94 in cardiomyocytes.

**Figure 5:**
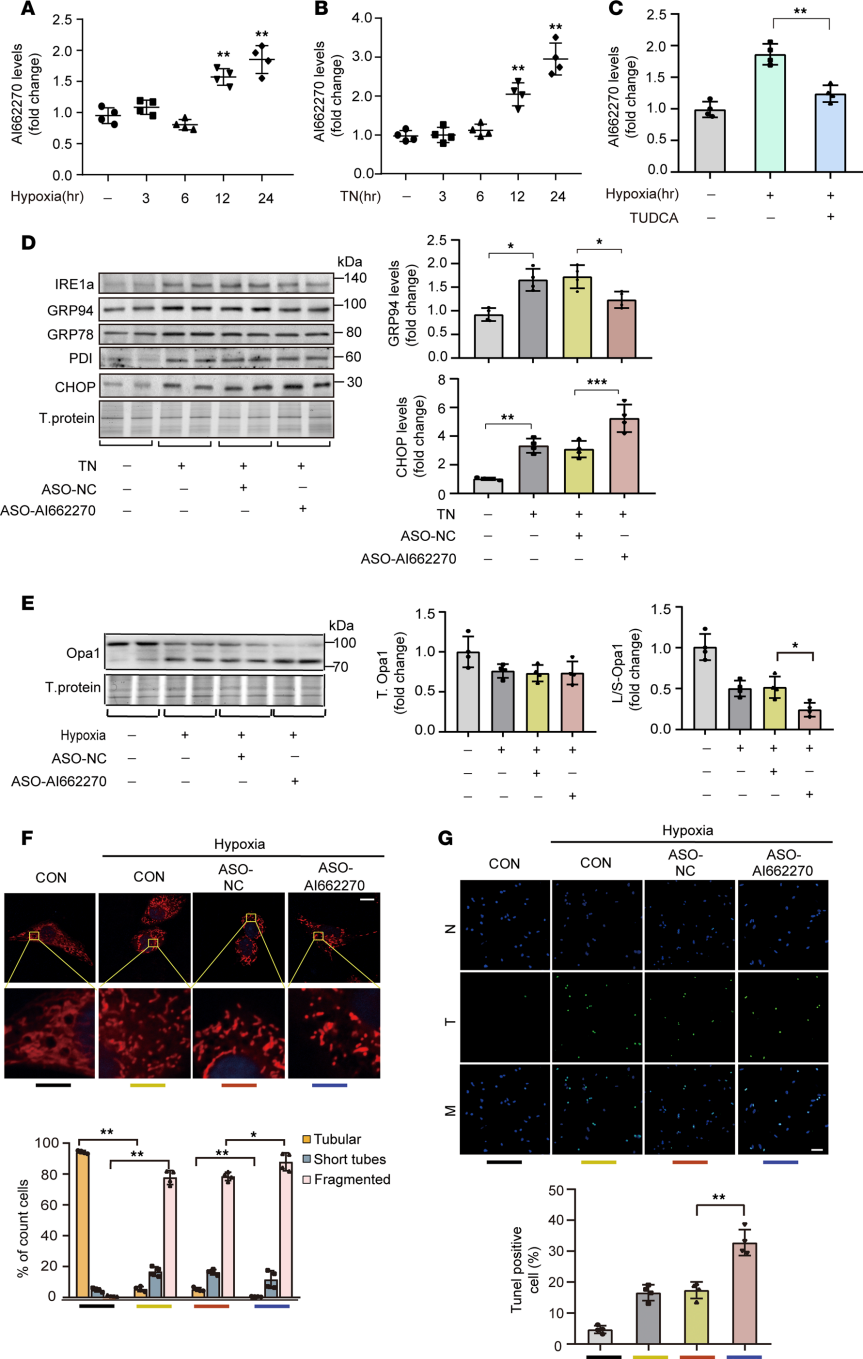
ER stress–induced AI662270 possesses a similar effect to GRP94 in vitro. (**A**) Hypoxia treatment induces an increase in AI662270 levels. Cardiomyocytes were exposed to hypoxia for the indicated durations. AI662270 mRNA analyzed by qRT-PCR (*n* = 4). (**B**) Tunicamycin (TN, 15 mg/L) induces AI662270 expression. Time-course analysis after TN treatment (*n* = 4). (**C**) ER stress inhibitor TUDCA (120 mg/L) blocks hypoxia-induced AI662270 upregulation. Cells were pretreated with TUDCA for 2 hours before 12-hour hypoxia (*n* = 4). (**D**) AI662270 knockdown specifically attenuates TN-induced GRP94. Cardiomyocytes were transfected with 40 nM ASO-AI662270/ASO-NC for 24 hours, and then treated with TN (15 mg/L, 12 hours). ER stress markers (IRE1α, GRP78, PDI, GRP94, CHOP) analyzed by immunoblotting (*n* = 4). (**E**) AI662270 silencing exacerbates hypoxia-induced Opa1 cleavage. Cells transfected with ASO-AI662270/ASO-NC were exposed to hypoxia (12 hours). Opa1 protein was analyzed by immunoblot. (**F**) AI662270 deficiency promotes mitochondrial fragmentation. Mitochondrial morphology was assessed by MitoTracker staining (≥100 cells/group). Categories: filamentous (>80% tubular), intermediate (30%–80%), fragmented (<30%). Scale bar: 20 μm (*n* = 4). (**G**) AI662270 knockdown enhances hypoxia-induced apoptosis. TUNEL assay in cells treated as in **E**. Scale bar: 50 μm (*n* = 4). *P < 0.05; **P < 0.01 versus the indicated group by 1-way ANOVA with Tukey-Kramer post hoc analysis (**A**–**E** and **G**) or 2-way ANOVA with Bonferroni’s multiple-comparison test (**F**). All data are shown as mean ± SD.

To explore the underlying role of lncRNA AI662270 in cardiomyocytes, we determined the level of change in AI662270 under different stimuli. AI662270 exhibited obvious upregulation following H_2_O_2_ and hypoxia exposure (Figure 5A and Supplemental Figure 5A). Similarly, tunicamycin (TN), an ER stress inducer, aggravated the level of AI662270 (Figure 5B). Hypoxia-triggered AI662270 induction was markedly attenuated by cotreatment with the ER stress inhibitor tauroursodeoxycholic acid (TUDCA) (Figure 5C). These results suggested AI662270 is likely uniquely induced by ER stress. Next, the effect of AI662270 on ER stress markers was detected. TN treatment upregulated key ER stress indicators, including IRE1α, PDI, GRP94, GRP78, and the marker of ER stress–induced apoptosis, CHOP. Among these, only the level of GRP94 was reduced after AI662270 silencing, accompanied by the increased CHOP (Figure 5D and Supplemental Figure 5B). Consistent with GRP94 as shown in Figure 2E, AI662270 deletion did not alter total Opa1 levels but promoted hypoxia-induced cleavage of Opa1 to S-Opa1 (Figure 5E). Consequently, AI662270 deletion intensified mitochondrial fragmentation in hypoxia-treated cardiomyocytes (Figure 5F), accompanied by more severe apoptosis (Figure 5G). Together, these data demonstrate the potential role for lncRNA AI662270 in regulating ER stress and mitochondrial dynamics.

### AI662270 protects against AMI.

Absolute qRT-PCR in cardiomyocytes isolated from Sham and MI models revealed low basal AI662270 expression in control cardiomyocytes that substantially increased after MI. In contrast, cardiac fibroblasts exhibited stable, low AI662270 levels (Supplemental Figure 6A).

**Figure 6:**
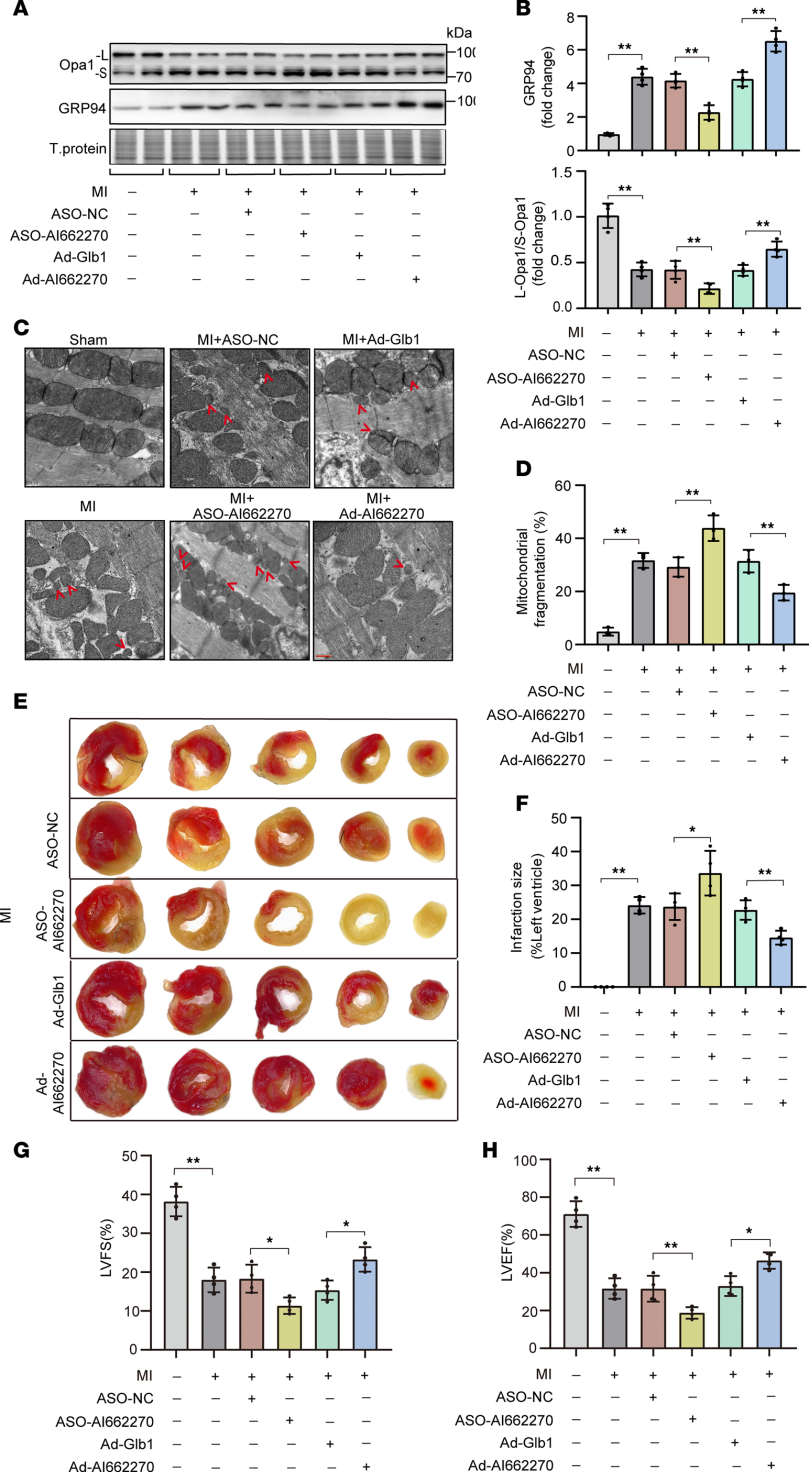
Lnc-AI662270 alleviates post-MI cardiac dysfunction via GRP94 and mitochondrial homeostasis. (**A** and **B**) AI662270 regulates GRP94 and L-Opa1 in vivo. C57BL/6 mice (8–10 weeks old) injected with AAV2/9-AI662270 (1 × 10^11^), Ad-Glb1, ASO-AI662270 (40 nM), or ASO-NC underwent permanent LAD ligation. (**A**) GRP94 and Opa1-L protein levels at 24 hours after MI. (**B**) Quantification of L-Opa1/S-Opa1 ratio (*n* = 4). (**C** and **D**) AI662270 suppresses MI-induced mitochondrial fragmentation. (**C**) TEM images of cardiomyocyte mitochondria. (**D**) Quantification of fragmented mitochondria (*n* = 3). Scale bar: 1 μm. (**E** ansd **F**) AI662270 reduces infarct size. (**E**) TTC-stained heart sections (red, viable; white, infarct). (**F**) Infarct area quantification at 24 hours after MI (*n* = 4). (**G** and **H**) AI662270 preserves cardiac function. Echocardiography at 1 week after MI. (**G**) LVEF and (**H**) LVFS (*n* = 4). LVEF, left ventricular ejection fraction; LVFS, left ventricular fractional shortening. *P < 0.05; **P < 0.01 versus the indicated group by 1-way ANOVA with Tukey-Kramer post hoc analysis (**B**, **D**, and **F**–**H**). All data are shown as mean ± SD.

To test the role of AI662270 in vivo, cTnT promoter–driven adenovirus harboring AI662270 or AI662270 antisense oligonucleotide (ASO), which could effectively manipulate the levels of AI662270 in heart (Supplemental Figure 6A), was intravenously delivered into mice on 1 day prior to MI surgery. The silencing of AI662270 attenuated the early-phase upregulation of GRP94 on the first day after MI, whereas its overexpression amplified GRP94 induction (Figure 6, A and B). Consistent with the in vitro results, AI662270 manipulation modulated Opa1 proteolytic processing, with overexpression specifically favoring the cleavage of Opa1 to L-Opa1 but not S-Opa1 (Figure 6, A and B, and Supplemental Figure 6B).

Transmission electron microscopy (TEM) confirmed that AI662270 deletion potentiated mitochondrial fragmentation caused by MI, which was mitigated by AI662270 enforcement (Figure 6, C and D). Staining with 2,3,5-triphenyl tetrazolium chloride (TTC) demonstrated the exacerbated MI size responding to AI662270 deletion and the attenuated MI size responding to AI662270 overexpression at 1 day after MI (Figure 6, E and F). Echocardiography studies revealed a markedly reduced LVEF (Figure 6G) and LVFS (Figure 6H) in the AI662270 ASO group compared with the negative control group at 7 days after MI. Per contra, AI662270 overexpression substantially improved cardiac function at 7 days after MI as shown by LVEF (Figure 6G) and LVFS (Figure 6H) in mice. Taken together, AI662270, GRP94, and Opa1 constitute an axis coupling ER stress response with mitochondrial dynamics during AMI.

### GRP94 interacts with Anxa2 to regulate Opa1 proteolytic cleavage.

**Figure 7:**
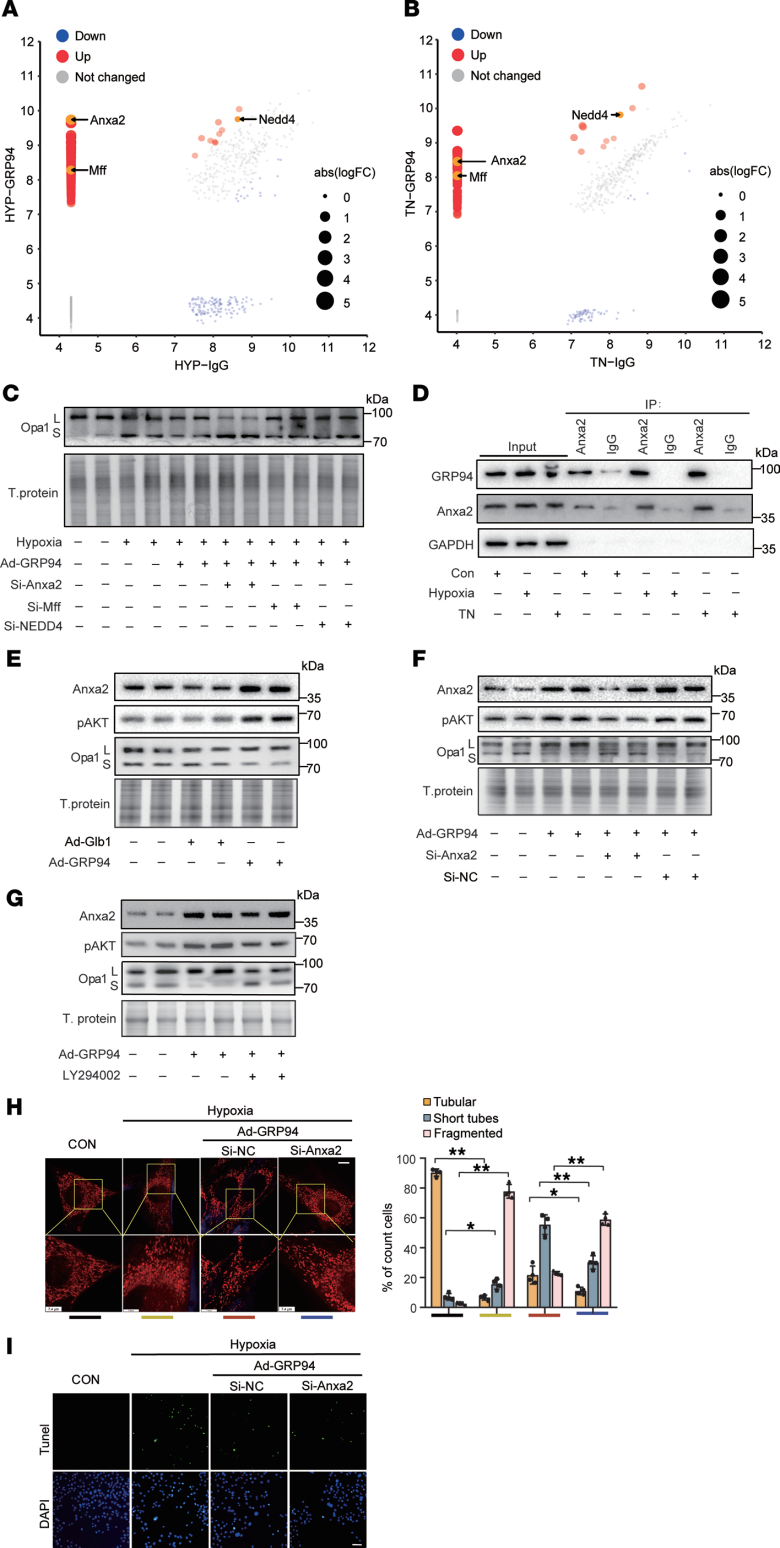
GRP94 interacts with Anxa2 to regulate the cleavage of Opa1. (**A** and **B**) Hypoxia and ER stress remodel GRP94 interactomes. Cardiomyocytes exposed to (**A**) hypoxia (12 hours) or (**B**) tunicamycin (TN, 15 mg/L, 12 hours). GRP94 coimmunoprecipitated proteins identified by LC-MS/MS. (**C**) Anxa2 knockdown (si-Anxa2, 40 nM) blocks GRP94-mediated L-Opa1 stabilization. Cells infected with Ad-*GRP94* or Ad-*Glb1* (MOI = 50) and transfected with si-Anxa2/si-NC for 36 hours, followed by hypoxia (12 hours). Opa1 isoforms analyzed by immunoblotting (*n* = 4). (**D**) Endogenous GRP94-Anxa2 interaction under hypoxia. Co-IP with anti-GRP94 or IgG in hypoxic cardiomyocytes (12 hours). (**E**) GRP94 overexpression enhances Anxa2 and p-Akt1 levels. Ad-GRP94–infected cells (MOI = 50, 48 hours). Anxa2, p-Akt1 (Ser473), and L-Opa1 analyzed (*n* = 4). (**F**) Anxa2 deletion abrogates GRP94-induced p-Akt1 and L-Opa1. Ad-GRP94 (MOI = 50) + si-Anxa2/si-NC (40 nM, 48 hours). Protein levels quantified (*n* = 4). (**G**) Akt1 inhibitor LY294002 (50 μM) reverses GRP94 effects. Ad-GRP94–infected cells treated with LY294002 for 48 hours (*n* = 4). Anxa2, p-Akt1, and Opa1 protein was analyzed by immunoblot (*n* = 4). (**H**) Anxa2 knockdown negates GRP94-mediated mitochondrial protection. Mitochondrial morphology was assessed by MitoTracker (≥50 cells/group). Categories: filamentous (>80% tubular), intermediate (30%–80%), fragmented (<30%). Scale bar: 20 μm (*n* = 4). *P < 0.05; **P < 0.01 versus the indicated group by 2-way ANOVA with Bonferroni’s multiple-comparison test. (**I**) Anxa2 deletion abolishes the GRP94 antiapoptotic effect. TUNEL assay in cells treated as in **F**. Scale bar: 50 μm (*n* = 4). All data are expressed as mean ± SD.

To elucidate the molecular mechanisms underlying GRP94-mediated regulation of Opa1 proteolytic processing, the interactors of GRP94 were isolated by coimmunoprecipitation (co-IP) with anti-GRP94 antibody from the lysate of cells with hypoxia or ER stress inducer (TN) treatment and analyzed by liquid chromatography coupled with tandem mass spectrometry (LC/MS-MS) (Supplemental Tables 3 and 4). Proteomic profiling identified Anxa2 as the top-ranked GRP94 interactor under hypoxia (Figure 7A) and the 11th candidate under TN treatment (Figure 7B). Furthermore, NEDD4 and Mff were also identified and caught our attention (Figure 7, A and B) due to their reported relationship with mitochondrial dynamics (32, 33). However, only Anxa2 knockdown substantially inhibited the cleavage of Opa1 to L-Opa1 induced by GRP94 (Figure 7C and Supplemental Figure 7A). Mutual co-IP confirmed the interaction between GRP94 and Anxa2 under hypoxia or TN treatment (Figure 7D and Supplemental Figure 7B). GRP94 overexpression upregulated Anxa2 protein expression without altering its mRNA levels (Figure 7E and Supplemental Figure 7, C and D), concomitant with enhanced phosphorylation of Akt1 (p-Akt1) and L-Opa1 accumulation (Figure 7E). This is consistent with the recent report that Anxa2 regulates the activation of Akt1 signaling to maintain the protein level of L-Opa1 (34). Notably, Anxa2 silencing abolished GRP94-induced p-Akt1 elevation and L-Opa1 generation (Figure 7F and Supplemental Figure 7E). LY294002, the inhibitor of Akt1 phosphorylation, blocked GRP94-mediated Opa1 processing (Figure 7G and Supplemental Figure 7F). Functionally, Anxa2 knockdown inhibited GRP94-attenuated mitochondrial fragmentation (Figure 7H) and cell apoptosis (Figure 7I and Supplemental Figure 7G) induced by hypoxia treatment. Similarly, AI662270 silencing reduced the protein levels of Anxa2 and p-Akt1, while AI662270 overexpression upregulated the protein levels of Anxa2 and p-Akt1, as GRP94 did (Supplemental Figure 7, H and I). These findings establish GRP94 as a critical regulator of mitochondrial integrity through Anxa2/Akt1-dependent control of Opa1 processing during hypoxic stress.

In addition, immune checkpoint inhibitors (ICIs), such as anti–PD-1 monoclonal antibodies, have revolutionized cancer therapy. However, patients receiving anti–PD-1 antibodies show a high incidence of cardiac adverse events (35, 36). The mechanisms underlying ICI-induced cardiotoxicity remain poorly understood. In this study, GRP94 levels were reduced in the cardiac tissue of mice treated with 200 μg of anti–PD-1 3 times per week for 2 weeks. This reduction was accompanied by lower levels of Anxa2 and L-Opa1 (Supplemental Figure 7J), indicating a potential role of the GRP94/L-Opa1 axis in PD-1 inhibitor–induced cardiotoxicity, which needs further exploration in the next work.

## Discussion

**Figure 8:**
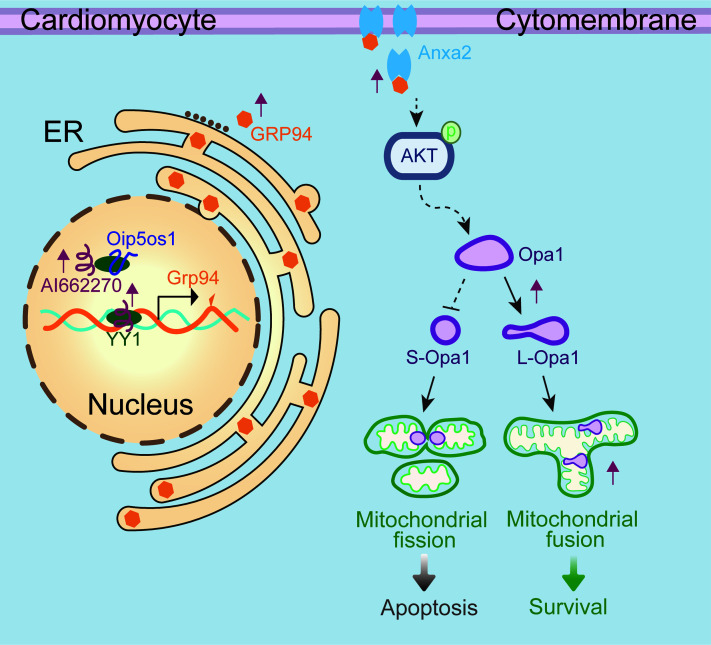
Model of AI662270/Yy1/GRP94/Opa1 signaling in coupling ER stress and mitochondrial dynamics. The red arrow represents the effect of the AI662270/GRP94 signaling axis activation induced by early hypoxic stress.

ER stress is increasingly considered the initial trigger of overall cellular stress. The occurrence of ER stress is often accompanied by mitochondrial dysfunction (37–39). These 2 processes are pathologically intertwined in multiple disease contexts, including cardiovascular (40, 41) and neurodegenerative disorders (42, 43). However, the mechanisms coupling ER stress and mitochondrial dysfunction are still largely blurred. Our current findings (Figure 8) demonstrate that GRP94, a key UPR effector triggered by ER stress, mitigates ischemia-induced mitochondrial hyper-fragmentation and promotes cardiomyocyte survival by stabilizing L-Opa1 via Anxa2/p-Akt1. Through assisting Yy1 binding to the GRP94 promoter, lncRNA AI226670 participates in modulating this ER-mitochondria crosstalk.

The initially discovered function of GRP94 was as a molecular chaperone in the ER, assisting in the folding and maturation of newly synthesized proteins such as Toll-like receptors and integrins (44). As research has progressed, GRP94 has been reported to interact with a diverse range of client proteins, including receptors, enzymes, and signaling molecules, to execute its diverse functions (15, 45). Understanding these interactions is crucial for deciphering the role of GRP94 in various cellular processes. Our study demonstrates that GRP94 physically interacts with Anxa2 to activate the p-Akt1 pathway, thereby regulating Opa1 proteolytic processing in hypoxic cardiomyocytes. Although GRP94 is primarily localized to the ER, it can also be found in the cytoplasm during cellular stress (46). Anxa2 can be found in the plasma membrane, cytosol, endosomal compartments, and occasionally the nucleus (47), where it orchestrates diverse cellular activities (48). Notably, we found that the interaction of GRP94 with Anxa2 enhances Anxa2 protein stability, although the structural determinants and spatiotemporal dynamics governing this interaction warrant systematic investigation.

While LeBeau et al. established that PERK-mediated eIF2α phosphorylation coordinates translational attenuation to drive protective mitochondrial hyperfusion during acute ER stress in fibroblasts (49), our study mechanistically extends this paradigm to myocardial pathophysiology. We identify GRP94, a downstream factor of the ATF6 branch (50), as a mediator of adaptive mitochondrial hyperfusion in myocardial ischemia. This evolutionary conservation of mitochondrial morphology regulation through distinct UPR branches (PERK vs. ATF6) highlights context-dependent stress adaptation mechanisms.

Although our work did not explore the regulatory role of ATF6 in GRP94 transcription, we propose that Yy1 and ATF6 may jointly regulate the transcription of GRP94 by forming a complex. This hypothesis builds on the evidence of Yy1 functioning as an indispensable coactivator for ATF6-driven GRP78 induction during ER stress (30), suggesting evolutionary conservation in UPR transcriptional machinery. The complexes participating in GRP94 transcriptional regulation and their precise architecture need systematic interrogation through chromatin occupancy studies and combinatorial knockdown approaches.

Yy1 can both activate and repress gene expression, which depends on the context and its cofactors. Recently, Yy1 has been found to interact with lncRNA to modulate the recruitment of other transcription factors and chromatin-modifying proteins to specific genomic loci, influencing gene expression patterns, exemplified by GAS5-Yy1 complexes enhancing PFKFB3 transcription in cerebral ischemia (51) and ANRIL-Yy1 interactions silencing tumor suppressors p15/p16 in cancer (52). Our findings extend this regulatory axis to cardiovascular pathophysiology; hypoxia induces a Yy1 switch from constitutive Oip5os1 binding to AI662270 association, with the latter directing Yy1 chromatin occupancy to the GRP94 promoter. This lncRNA-mediated target selection mechanism resolves the paradox of the pleiotropic gene regulation of Yy1, demonstrating the important role of lncRNAs as cofactors in determining the target genes of transcription factors. The structure of the interaction between Yy1 and AI662270 and the mechanism of AI662270 promoting Yy1 binding to the GRP94 promoter region need further exploration in future.

ER stress, as an actuating/collaborative mechanism of cellular stress, is increasingly recognized as a potential target for disease treatment in various pathological conditions, including neurodegenerative disorders such as Alzheimer disease (53) and Parkinson disease (54), metabolic disorders such as obesity (55) and type 2 diabetes (56), cardiovascular diseases (11), liver diseases (57), and oncogenesis (58). Targeting ER stress as a therapeutic strategy involves modulating the UPR, including the IRE1, PERK, and ATF6 branches. Each branch exhibits distinct activation mechanisms and pathology-specific signaling cascades that coordinate cellular adaptation. Thus, elucidating the regulatory mechanisms of each UPR factor holds great translational potential for developing stress-targeted clinical interventions. Notably, GRP94 serves as a master regulator of proteostasis, with its dysregulation mechanistically linked to oncogenesis (59), autoimmune disorders (60), and neurodegenerative diseases (61). While direct evidence of GRP94 dysregulation in human AMI remains limited, its upregulation in patients with atrial fibrillation (16) and elevated expression in heart failure tissues (GSE135055) suggest its conserved roles in cardiovascular pathophysiology. Our mechanistic discovery that AI662270 recruits Yy1 to activate GRP94 transcription could guide the development of GRP94-selective modulators, and potentially overcome the cross-reactivity with other Hsp90 family isoforms and associated toxicity issues. Although AI662270 lacks strict human orthologs, identification of its functional Yy1-binding motif (chr12q24.31 homologous region) provides a roadmap for discovering functional analogs in human noncoding transcriptomes. These findings position GRP94 as a therapeutic target for AMI, warranting validation in human induced pluripotent stem cell–derived cardiomyocyte models and patient-derived organoids.

Due to the intricate interplay between the ER and mitochondria in cellular homeostasis, energy metabolism, stress responses, and disease processes (62, 63), clarifying the molecular relationship between ER stress and mitochondrial function regulation is crucial for understanding cellular physiology, disease pathogenesis, and developing therapeutic strategies. The current study is the first to our knowledge to demonstrate the role of the AI662270/Yy1/GRP94/L-Opa1 axis in coupling ER stress response with mitochondrial dynamic regulation. We predict that manipulating AI662270 or its homolog could be exploited as a novel therapeutic approach to confer protection against mitochondrial over-fragmentation through specifically activating the GRP94 branch of the UPR in cardiac myocytes.

## Methods

### Sex as a biological variable.

Our study examined male and female animals, and similar findings are reported for both sexes.

### Treatment and culture of cardiomyocytes.

Primary cardiomyocytes were isolated from ventricles of postnatal day 1 or 2 C57BL/6 mice following established protocols (64). The mouse cardiac myocyte (MCM) cell line, sourced from the American Type Culture Collection (ATCC), was also utilized. Both cell types were maintained in Dulbecco’s modified Eagle medium/Nutrient Mixture F-12 (DMEM/F-12, Invitrogen/Gibco) supplemented with 10% fetal bovine serum (FBS) and 100 μg/mL penicillin/streptomycin at 37°C in a 5% CO_2_ atmosphere. For hypoxia induction, cells were subjected to hypoxic conditions (1% O_2_) within a specialized chamber for either 12 or 24 hours.

Adult mouse cardiomyocytes were isolated using a modified Langendorff-free perfusion method (65). Briefly, hearts were rapidly excised from anesthetized adult C57BL/6 mice (8–10 weeks old) and immediately immersed in ice-cold Krebs-Henseleit buffer. Retrograde perfusion of the left ventricle was achieved through a 26-gauge cannula, followed by enzymatic digestion at 37°C for 8–10 minutes using a solution containing 1.8 mg/mL collagenase II (Worthington, CLS-2) and 0.14 mg/mL protease XIV (Sigma-Aldrich). The digested tissue was minced, filtered through a 40 μm cell strainer, and subjected to differential centrifugation (200*g* for 3 minutes) to isolate high-purity cardiomyocytes. Cells were immediately lysed with TRIzol reagent (Invitrogen) for total RNA extraction, with RNA integrity verified using an Agilent 2100 Bioanalyzer (RIN > 8.0). For isolating adult mouse cardiac fibroblasts, a digestion buffer containing 2 mg/mL collagenase type IV (Worthington) and 1.2 U/mL Dispase II (Sigma-Aldrich) in DMEM was used (66).

### Adenoviral constructions, infection, and transfection of cells.

We utilized mouse cDNA as a template to amplify the full-length AI662270 via PCR. The forward primer sequence used was 5′-AAAGTCGCCTGGGGCCTCGGAGATG-3′ and the reverse primer sequence was 5′-AATGAGCAAGATCCAACAACTTTATT-3′. Mouse *GRP94* cDNA was procured from Origene. Adenoviruses carrying AI662270, Oip5os1, *GRP94*, and β-galactosidase (*Glb1*) were constructed using the Adeno-X expression system from Clontech. For silencing mouse GRP94 expression in vitro, the siRNA target sequence used was 5′-GCTATTCAGTTGGATGGGTTA-3′, with a scramble sequence 5′-CTCAGACTGCAGTACGACT-3′ serving as a control. The AdEasy Adenoviral Vector System (Hanbio) was employed to generate AAVs containing cTnT promoter–driven GRP94 siRNA, and their respective scramble controls as per the kit’s instructions. HEK293 cells were utilized for amplifying all constructs.

The siRNAs were purchased from Shanghai Genepharma Co., Ltd. *GRP94* siRNA sequences were 5′-GGGCUAUGAAGUCAUUUAUUU-3′ and 5′-AUAAAUGACUUCAUAGCCCUU-3′; *Cebpb* siRNA sequences were 5′-GAAGUGGCCAACUUCUACUUU-3′ and 5′-AGUAGAAGUUGGCCACUUCUU-3′; *c-Jun* siRNA sequences were 5′-GGCACAGCUUAAGCAGAAAUU-3′ and 5′-UUUCUGCUUAAGCUGUGCCUU-3′; *Cebpa* siRNA sequences were 5′-GCCGAGAUAAAGCCAAACAUU-3′ and 5′-UGUUUGGCUUUAUCUCGGCUU-3′; *Yy1* siRNA sequences were 5′-GCCAGAAUGAAGCCAAGAAUU-3′ and 5′-UUCUUGGCUUCAUUCUGGCUU-3′; *Mff* siRNA sequences were 5′-CGUGGUUACAGGAAAUAAUUU-3′ and 5′-AUUAUUUCCUGUAACCACGUU-3′; *Nedd4* siRNA sequences were 5′-GGCGAGUCUUCUUCAUAAAUU-3′ and 5′-UUUAUGAAGAAGACUCGCCUU -3′; *Anxa2* siRNA sequences were 5′-GCUGAAGUCAGCCUUAUCUUU-3′ and 5′-AGAUAAGGCUGACUUCAGCUU-3′. Lipofectamine 3000 (Invitrogen) was used for cell transfection.

The ASO sequence targeting AI662270 was 5′-GGTATCAAGAATAGTGATTGC-3′, with a scramble control sequence of 5′-AGTAAGTACGTAGTCATGGTA-3′; Oip5os1 ASO sequence used was 5′-GACCCACTGAAGTGACTAA-3′, with a scramble control sequence of 5′-AGTACGATCAGTACAGCAC-3′.

### RIP assay.

We conducted RIP using a Magna RIP RNA-Binding Protein Immunoprecipitation Kit (Merck) following the manufacturer’s protocol. Briefly, cardiomyocytes were lysed using RIP lysis buffer and subsequently incubated with protein A/G magnetic beads preconjugated with anti-Yy1 antibody overnight at 4°C. After thorough washing to remove nonspecifically bound materials, coprecipitated RNAs were extracted and quantified. qRT-PCR was employed to analyze specific RNAs coimmunoprecipitated with the anti-Yy1 antibody.

### RNA pull-down assay.

We employed the AmpliScribe T7-Flash Biotin-RNA Transcription Kit (Lucigen) for in vitro transcription and purification of biotin-labeled AI662270 and Oip5os1 probes. The biotin-labeled probes were incubated with cellular extracts for 2 hours. Subsequently, the mixture was incubated with streptavidin magnetic beads (Roche) at room temperature for 1 hour. After washing with the wash/binding buffer, the captured proteins were analyzed by Western blot.

### ChIP assay.

The ChIP assay was conducted as follows: Cells were washed with PBS and fixed for 10 minutes at room temperature with 1% formaldehyde. Cross-linking was quenched using 0.1 M glycine for 5 minutes. After 2 washes with PBS, cells were lysed in lysis buffer and incubated for 1 hour at 4°C. Chromatin was sonicated to yield fragments averaging 500 to 800 bp. Before immunoprecipitation, samples were precleared with Protein A–agarose (MedChemExpress) for 1 hour at 4°C on a rocking platform. Subsequently, 5 μg of specific antibodies were added and rocked overnight at 4°C. Immunoprecipitates were captured using Protein A–agarose blocked with salmon sperm DNA. DNA fragments were purified using the QIAquick Spin Kit (Qiagen). The purified DNA served as a template and was amplified using the following primer sequences: forward primer, 5′-ACAAGCCCAATCGCAAGGA-3′; reverse primer, 5′-ACTCAAGCACAAAGGGCAG-3′, for analyzing Yy1 binding to the GRP94 promoter region.

### Construction of mouse GRP94 promoter.

The GRP94 promoter was PCR amplified from mouse genomic DNA. The forward primer sequence for the full GRP94 promoter was 5′-GACTGACCATCTTCTGCCA-3′, and the reverse primer sequence was 5′-CCGTGCTAACCACTCAAGC-3′. Segments of the GRP94 promoter spanning –500 to +1 bp, –1000 to –501 bp, –1500 to –1001 bp, and –2000 to –1501 bp were synthesized separately by Shanghai Huagene Biotechnology Co., Ltd. The GRP94 promoter was cloned into the pGL4.17 vector (NovoPro) to generate the GRP94 promoter luciferase reporter plasmid, designated as pGL-GRP94. Site-directed mutagenesis of the putative binding site was performed using the QuikChange II XL Site-Directed Mutagenesis Kit (Stratagene), and the resulting constructs were sequenced to confirm the presence of desired mutations.

### Luciferase activity assay.

The Dual-Luciferase Reporter Assay System (Promega) was employed to conduct luciferase activity assays following the manufacturer’s instructions. Cells were transfected with 140 ng of either pGL-GRP94 or pGL-GRP94-mut plasmid using Lipofectamine 3000 (Invitrogen). Subsequently, the cells were infected with specified adenoviruses under normoxic or hypoxic conditions. Luciferase activity was measured 36 hours after infection.

### Western blot analysis.

Proteins were separated using 10% or 12% SDS-PAGE gels (Beyotime Institute of Biotechnology) and transferred onto PVDF membranes (Millipore). Total protein bands were visualized under UV light using the ChemiDoc Imaging System (Bio-Rad). The membranes were blocked with 5% (w/v) nonfat dry milk (Bio-Rad) in Tris-buffered saline with 0.1% Tween 20 (TBST; 10 mM Tris pH 7.5, 150 mM NaCl, 0.1% Tween 20) for 1 hour at room temperature, followed by overnight incubation at 4°C with the following primary antibodies: anti-GRP94 (Abcam, ab52031), anti-Mfn2 (Abcam, ab56889), anti–p-DRP1 (Abcam, ab314755), anti-DRP1 (Abcam, ab184247), anti-Opa1 (Cell Signaling Technology, 80471), anti-CHOP (Cell Signaling Technology, ER Stress Antibody Sampler Kit, 9956), anti-FIS1 (Abcam, ab229969), anti-IRE1a (Cell Signaling Technology, ER Stress Antibody Sampler Kit, 9956), anti-PDI (Cell Signaling Technology, ER Stress Antibody Sampler Kit, 9956), anti-Grp78 (Cell Signaling Technology, ER Stress Antibody Sampler Kit, 9956), anti-Yy1 (Abcam, ab109237), anti-Axna2 (Abcam, ab41803), and anti–p-Akt (Ser473) (Cell Signaling Technology, 4058). After washing, membranes were incubated for 1 hour at room temperature with an HRP-conjugated anti-mouse or anti-rabbit IgG secondary antibody (Cell Signaling Technology) diluted 1:5000. Protein bands were visualized using ECL luminol reagent (Millipore) and the ChemiDoc Imaging System. Band intensities were quantified using ImageJ software (NIH).

Through optimization of immunoblotting conditions, it was found that 6% SDS-PAGE with extended electrophoresis resolved 5 Opa1 bands in cardiomyocytes (Supplemental Figure 2I), consistent with Wai et al. (29). Instead, 10%–12% gels selectively detected 2 bands reflecting L-Opa1/S-Opa1 dynamics (Supplemental Figure 2I), aligning with recent cardiac studies (67, 68), ensuring reproducible quantification of functionally relevant proteolytic isoforms.

### RNA isolation and RT-PCR.

RNA extraction from cultured cells was performed using TRIzol following the manufacturer’s protocol. Total RNA (1 μg) was reverse transcribed using the miScript Reverse Transcription Kit (Qiagen). The resulting cDNA was amplified using the SsoFast EvaGreen Supermix (Bio-Rad) in a 2-step PCR protocol: initial denaturation at 95°C for 30 seconds, followed by 40 cycles of denaturation at 95°C for 2 seconds and annealing/extension at 60°C for 5 seconds. The specific PCR primers used for Ddit3 were 5′-TCACTACTCTTGACCCTGCG-3′ and 5′-ACTGACCACTCTGTTTCCGT-3′; for Ppp1r15a were 5′-CCTCCTGAAACTTGGGGACT-3′ and 5′-GCTGTGATGTGGGATAAGCG-3′; for Atf6 5′-AACCGAGAGTCTGCTTGTCA-3′ and 5′-AGCCTCTGGTTCTCTGACAC-3′; for Atf4 were 5′-GACCGAGATGAGCTTCCTGA-3′ and 5′-GCCAAGCCATCATCCATAGC-3′; for Atf3 were 5′-GACCCCTGGAGATGTCAGTC-3′ and 5′-CTCCTCAATCTGGGCCTTCA-3′; for Ern2 were 5′-CTGCAGGAAGAGACCCATGA-3′ and 5′-GGACCACCTTGTAGCTTCCT-3′; for Hspa5 were 5′-CTTTGATCAGCGGGTCATGG-3′ and 5′-AGCTCTTCAAATTTGGCCCG-3′; for Canx were 5′-AAGACCCAGAAGACCGGAAG-3′ and 5′-ACTCAGGTTCGTCGTCTAGC-3′; for Ero1l were 5′-TTAACCCTGAGCGCTACACA-3′ and 5′-GCGTGCAGGCCAGATATAAG-3′; for Xbp1 were 5′-TGTCACCTCCCCAGAACATC-3′ and 5′-AAGGGAGGCTGGTAAGGAAC-3′; for Erp44 were 5′-TCAGAAGACACCTGCCGATT-3′ and 5′-TTCTCCTCTTGCTGTCCTGG-3′; for Hsp90b1 were 5′-AGTCGGGAAGCAACAGAGAA-3′ and 5′-TCTCCATGTTGCCAGACCAT-3′; for Calr were 5′-AGCAGTTCTTGGACGGAGAT-3′ and 5′-AGTGTCTGGCCCTTATTGCT-3′; for Edem1 were 5′-GAACACCTGGATTGACTCGC-3′ and 5′-GAAGAGAACATCAGGGGCCT-3′; for Dnajc3 were 5′-TAAGGATGAGAAGCCCGTGG-3′ and 5′-TCCCGAATCTGCTGGTCATT-3′; for Pdia3 were 5′-CGATGTGTTGGAACTGACGG-3′ and 5′-AGAGTTGGGTAGCCACTGAC-3′; for Pdia4 were 5′-GACACCTCCACCTGAAGTCA-3′ and 5′-AGAGGCCTGTGTGTGTAGAC-3′; for AI662270 were 5′-TGATTCAACCACCACCCTCA-3′ and 5′-CCAATTGTTCCCCACGGTTT-3′; for Gm12840 were 5′-TTGGTTTGGTTTTAGGCCGG-3′ and 5′-TCAGTCCAAGTCCGAACACA-3′; for Gm20186 were 5′-TGGCTGACTAACGCTCTTGA-3′ and 5′-TCCGTGTACCAGCTCAAACT-3′; for Snhg18 were 5′-ATTTAGGGTCTGGCTTGGCT-3′ and 5′-TCCTTCTTTGGATGTGGCCT-3′; for Lppos were 5′-GCAAAGAGATCCTGCCATGG-3′ and 5′-AATACTGGCTCCTCTCCACG-3′; for Oip5os1 were 5′-GCAAAGAGATCCTGCCATGG-3′ and 5′-AATACTGGCTCCTCTCCACG-3′; for 1810058I24Rik were 5′-GGTTCCTGTGTGCCTTTGAG-3′ and 5′-GAGGCAGGAGGGTCAGAAAT-3′; for 2010320M18Rik were 5′-CAGAAGTTGCCGAGCTTGTT-3′ and 5′-AACCTGGATCCTGTGCAAGA-3′; for A430046D13Rik were 5′-GCCCAGGAGAGGTTAAAGGT-3′ and 5′-GGTCACTTCTCTGGCTCTGT-3′; for Gm5532 were 5′-CCTCTGTTGTTGTTGCTGCT-3′ and 5′-TCATCCTGCCCTCTATGCTG-3′; for AW112010 were 5′-GTGTGCTCATCATCTGCCTG-3′ and 5′-GATCTCTCCTGAACGACCCA-3′. Quantification of gene expression levels was conducted using the ΔΔCt method, where ΔΔCt = ΔCt (target gene) − ΔCt (reference gene), and normalized to HPRT1 mRNA levels within the same cells.

Absolute quantification for AI662270 was conducted following established methodologies (69, 70). Standard curve construction involved molecular cloning of the AI662270 sequence into the T7 promoter–containing pcDNA3.1 vector, followed by in vitro transcription using the TranscriptAid T7 High Yield Transcription Kit (Thermo Fisher Scientific). Purified RNA transcripts (478.72 kDa molecular weight) were serially diluted to generate a calibration curve spanning 6 orders of magnitude (10²–10^8^ copies/reaction). All reactions were performed on an ABI QuantStudio 5 Flex system.

### Immunoprecipitation.

Total GRP94, Anxa2, and rabbit IgG control antibodies were conjugated overnight with Protein A/G Magnetic Beads (MedChemExpress), which were first blocked by incubating in 2% fatty acid–free BSA (Beyotime) on a rotator. Conjugated beads were washed with phosphate lysis buffer 3 times and incubated with cardiomyocyte lysate overnight on a rotator. The next day, beads were washed with PLB and boiled upon adding 5× SDS loading buffer for 5 minutes at 95°C. Proteins were then separated by SDS-PAGE and stained with Coomassie blue. The bands were cut from the gel and subjected to in-gel digestion with trypsin. Peptides were extracted from the gel pieces by sonication for 15 minutes, followed by centrifugation and supernatant collection. The samples were analyzed by LC-MS/MS by Luming Biotechnology Co., Ltd.

### TEM.

Left ventricular papillary muscles were isolated from mouse hearts and fixed in 2.5% glutaraldehyde in cacodylate buffer (150 mM Na-cacodylate, 2 mM CaCl_2_, pH 7.3) for 1 hour. The specimens were then postfixed in 1% osmium tetroxide in cacodylate buffer for 30 minutes and stained with 1% aqueous uranyl acetate solution. After dehydration in a series of graded ethanol and acetone solutions, the tissue was embedded in Durcupan (ACM Fluka). Longitudinal ultrathin sections (58–60 nm) were cut using an ultramicrotome (Power-Tome MT-XL, RMC/Sorvall). These sections were mounted on formvar-coated copper grids, stained with lead citrate, and observed under a JEM 1200 electron microscope (JEOL) at 80 kV. Random images of cardiomyocytes were captured using a CCD camera (Gatan DualVision 300W) at a magnification of ×15,000.

### Animals and treatments.

We obtained C57BL/6 mice from the Department of Laboratory Animal Science, Fudan University. All mice were housed under specific pathogen–free conditions in an animal room with a 12-hour day/night cycle with free access to water and food. The myocardial ischemic model was established by ligating the left anterior descending coronary artery to cause myocardial ischemia as we described previously (71). Briefly, mice were anesthetized by inhalation of isoflurane, intubated with a 22-gauge intravenous catheter, and then were fully anesthetized with 1.0%–2.0% isoflurane gas while being mechanically ventilated on a positive pressure ventilator. Left thoracotomy was performed at the fourth intercostal space, and the pericardium was stripped to expose the heart. The left descending coronary artery was identified and occluded with an 8-0 silk ligature that was placed around it. The success of the ligation was confirmed when the anterior wall of the left ventricle turned pale. The chest cavity was closed, and the animal was placed in a cage on a heating pad. Sham-operated mice underwent the same surgical procedures except that the suture placed under the left anterior descending artery was not tied. AAVs expressing *GRP94*, *GRP94*-siRNA, *Glb1*, and AI662270 were injected immediately after left anterior descending ligation into the myocardium bordering the infarct zone at a dose of 1 × 10^11^ viral genome particles per animal using an insulin syringe with a small gauge needle. Similarly, AI662270 ASO and negative control were injected after left anterior descending ligation into the myocardium bordering the infarct zone at a dose of 10 nmol per animal using an insulin syringe with a small-gauge needle. The chest was closed, and the animals were moved back to cages after the occurrence of spontaneous breathing. Cardiac function of these groups of animals was evaluated by echocardiographic analysis 7 days after the surgery.

Cardiac tissues from the liver tumor–bearing mouse model were provided by Lin HanChao at Fudan University Affiliated Minhang Hospital in Shanghai.

### Echocardiography.

On day 7 after ligation, parasternal long-axis echocardiography was conducted using the Vevo 2100 system. Mice were anesthetized with isoflurane and placed on a heated pad to maintain body temperature. Parasternal long-axis M-mode echocardiographic images were acquired using the Vevo 2100. Parameters, including heart rate, LVEF, and LVFS, were analyzed using Vevo Lab Software.

### Measurement of infarct size.

Mice were euthanized with an overdose of sodium pentobarbital, and their hearts were subsequently excised. Each heart was transversely sectioned into 5 slices, which were then incubated in 0.5% TTC (Sigma-Aldrich) at 37°C for 15 minutes. The noninfarcted tissue stained red, whereas the infarcted tissue remained unstained (white). Infarct size (%) was calculated by dividing the area of the infarct by the total area of the slice.

### Statistics.

All data are expressed as mean ± standard deviation (SD). Statistical analyses were performed using GraphPad Prism 7.0. Parametric statistics were assessed using a 2-tailed Student’s *t* test for 2 groups. One-way ANOVA with Tukey’s post hoc test or 2-way ANOVA with Bonferroni’s multiple-comparison test was performed for multiple-group analysis. Nonparametric data were evaluated by the Kruskal-Wallis test with Dunn’s correction. A *P* value of less than 0.05 was considered statistically significant.

### Study approval.

This study was performed in strict accordance with the recommendations from the Guide for Animal Management Rules from the Ministry of Health of the People’s Republic of China. All animal experiments were reviewed and approved by the Animal Ethics Committee of Zhongshan Hospital of Fudan University.

### Data availability.

scRNA-seq raw data are accessible through the NCBI Gene Expression Omnibus (GEO GSE304427). All graphical data points are provided in the Supporting Data Values file, with additional processed data and images available upon request.

## Author contributions

SD and WL conceptualized the study and developed methodology. SD and ZZ curated data, and wrote the original draft of the manuscript. Xiyang Yang analyzed data. DS and XZ generated figures and conducted experiments. JZ provided software, supervision, and study validation. JG and Xiangdong Yang reviewed and edited the manuscript. All authors discussed the results and commented on the manuscript.

## Supplementary Material

Supplemental data

Unedited blot and gel images

Supporting data values

## Version 1 10/08/2025

Electronic publication

## Version 2 10/16/2025

Junbo Ge's affiliation was updated
