# Supplementary material for: AI662270/GRP94 axis couples the unfolded protein response to mitochondrial dynamics during acute myocardial infarction
Source: JCI Insight. 2025 Oct 8;10(19):e188904. doi: 10.1172/jci.insight.188904 (PMC12513489; doi:10.1172/jci.insight.188904)
Supplement: Supplemental data [file jciinsight-10-188904-s009.pdf]

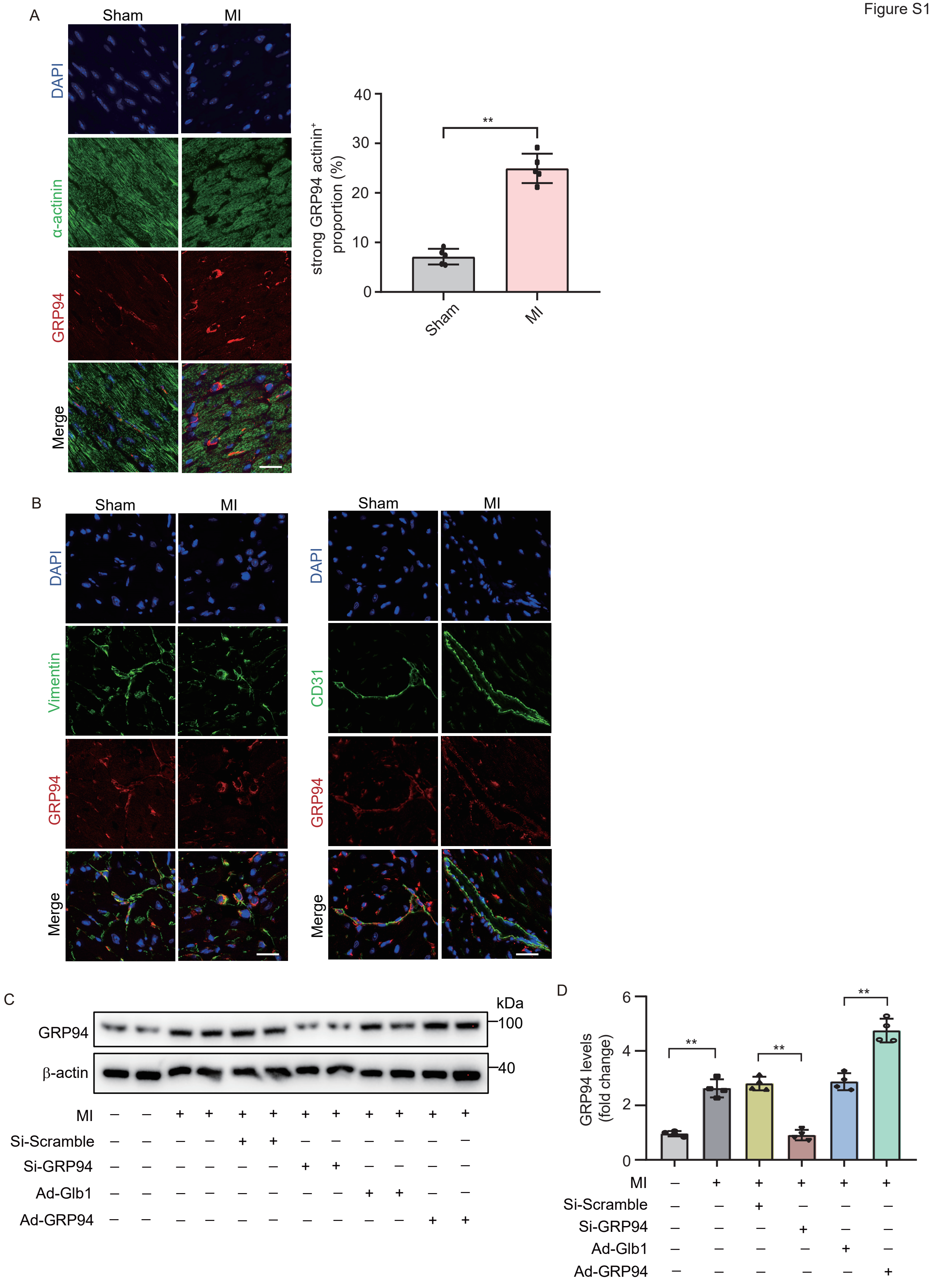

A
Sham
MI
DAPI
α-actinin
GRP94
Merge
strong GRP94 actinin+ proportion (%)
40
30
20
10
0
**
Sham
MI
B
Sham
MI
DAPI
Vimentin
GRP94
Merge
Sham
MI
DAPI
CD31
GRP94
Merge
C
kDa
GRP94
100
β-actin
40
MI – – + + + + + + + + + +
Si-Scramble – – – – + + – – – – – –
Si-GRP94 – – – – – – + + – – – –
Ad-Glb1 – – – – – – – – + + – –
Ad-GRP94 – – – – – – – – – – + +
D
GRP94 levels (fold change)
6
4
2
0
**
**
**
MI – + + + + +
Si-Scramble – – + – – –
Si-GRP94 – – – + – –
Ad-Glb1 – – – – + –
Ad-GRP94 – – – – – +

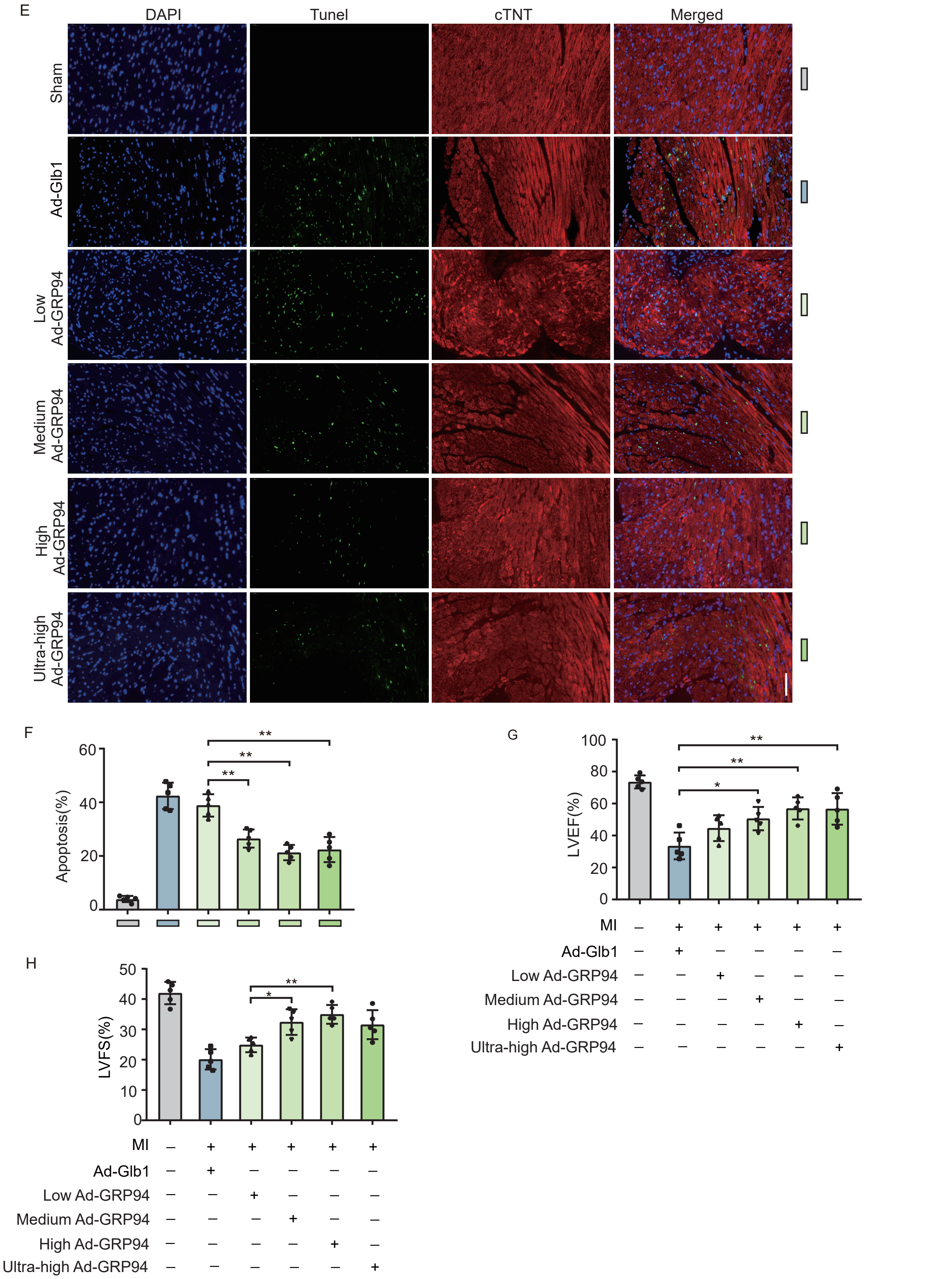

E
DAPI
Tunel
cTNT
Merged
Sham
Ad-Glb1
Low Ad-GRP94
Medium Ad-GRP94
High Ad-GRP94
Ultra-high Ad-GRP94
F
Apoptosis(%)
60
40
20
0
**
**
**
G
LVEF(%)
100
80
60
40
20
0
**
**
*
MI – + + + + +
Ad-Glb1 – + – – – –
Low Ad-GRP94 – – + – – –
Medium Ad-GRP94 – – – + – –
High Ad-GRP94 – – – – + –
Ultra-high Ad-GRP94 – – – – – +
H
LVFS(%)
50
40
30
20
10
0
**
*
MI – + + + + +
Ad-Glb1 – + – – – –
Low Ad-GRP94 – – + – – –
Medium Ad-GRP94 – – – + – –
High Ad-GRP94 – – – – + –
Ultra-high Ad-GRP94 – – – – – +

Supplementary Figure 1. Effective modulation of GRP94 levels in vivo. (**A)** Immunofluorescence co-staining analysis of GRP94 with cardiomyocyte marker α-actinin in mouse heart sections (scale bar: 40 μm). The proportion of GRP94 strong signaling cells co-located with cTNT was statistically analyzed. Analysis is performed with unpaired two-tailed Student's t-test (*P<0.05, **P<0.01). (**B)** Immunofluorescence co-staining analysis of GRP94 with fibroblast marker Vimentin, and endothelial marker CD31 in mouse heart sections (scale bar: 40 μm). (**C-D)** Adult male C57 mice (8–10 weeks old) were injected with Adeno-Associated Virus2/9-GRP94, Glb1, GRP94 siRNA, or siRNA scramble immediately after permanent left anterior descending coronary artery ligation. GRP94 levels were detected by immunoblot (n=4). *P<0.05 and **P<0.01 versus untreated group in one-way ANOVA followed by Tukey–Kramer post hoc analysis. (**E-F)** GRP94 dose-dependently lowered cardiomyocyte apoptosis at 1 day post-MI. C57BL/6 mice (8-10 weeks) received intramyocardial injections of graded AAV2/9-GRP94 doses (Low: 2×10^{10}; Medium: 1×10^{11}; High: 2×10^{11}; Ultra-high: 4×10^{11} viral genomes) or Ad-Glb1 control (1×10^{11} genomes) into the peri-infarct myocardium immediately after left anterior descending coronary artery ligation. Representative images show TUNEL-positive nuclei (green), DAPI counterstain (blue), and α-actinin-labeled cardiomyocytes (red; scale bar: 40 μm; n=5). Statistical analysis used one-way ANOVA with Tukey-Kramer post hoc test (*P<0.05, **P<0.01). **(G-H)** GRP94 markedly improved cardiac function post-MI in a dose-dependent manner. Mice were treated as in (E), with transthoracic echocardiography performed 1 week post-MI (n=5). Left ventricular ejection fraction (LVEF) and fractional shortening (LVFS) were analyzed by one-way ANOVA with Tukey-Kramer test (*P<0.05, **P<0.01). Data are shown as mean ± SD.

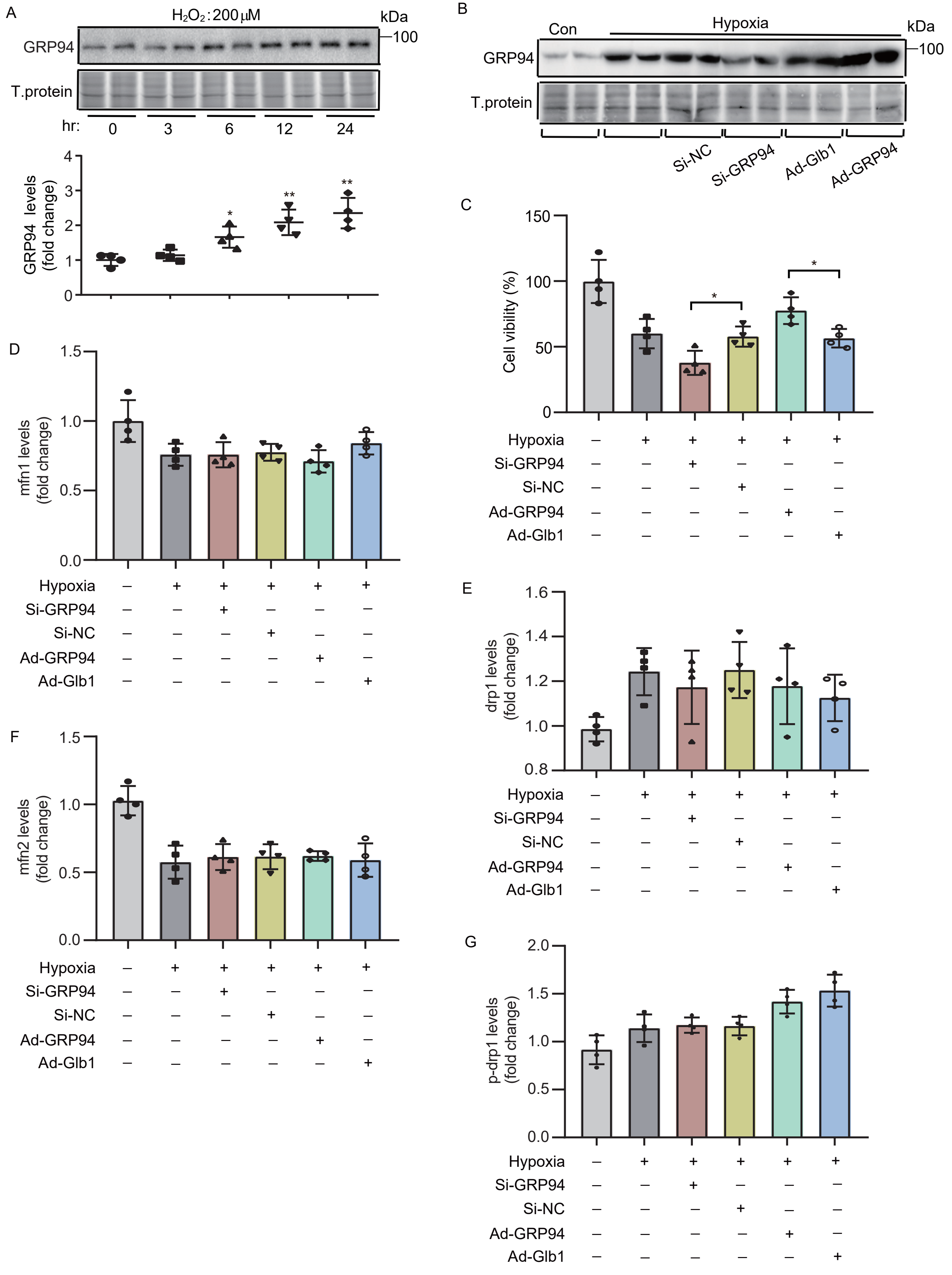
A
H_2O_2: 200 μM
kDa
GRP94
100
T.protein
hr: 0 3 6 12 24
GRP94 levels (fold change)
B
Con
Hypoxia
kDa
GRP94
100
T.protein
Si-NC
Si-GRP94
Ad-Glb1
Ad-GRP94
C
Cell vibility (%)
Hypoxia
Si-GRP94
Si-NC
Ad-GRP94
Ad-Glb1
D
mfn1 levels (fold change)
Hypoxia
Si-GRP94
Si-NC
Ad-GRP94
Ad-Glb1
E
drp1 levels (fold change)
Hypoxia
Si-GRP94
Si-NC
Ad-GRP94
Ad-Glb1
F
mfn2 levels (fold change)
Hypoxia
Si-GRP94
Si-NC
Ad-GRP94
Ad-Glb1
G
p-drp1 levels (fold change)
Hypoxia
Si-GRP94
Si-NC
Ad-GRP94
Ad-Glb1

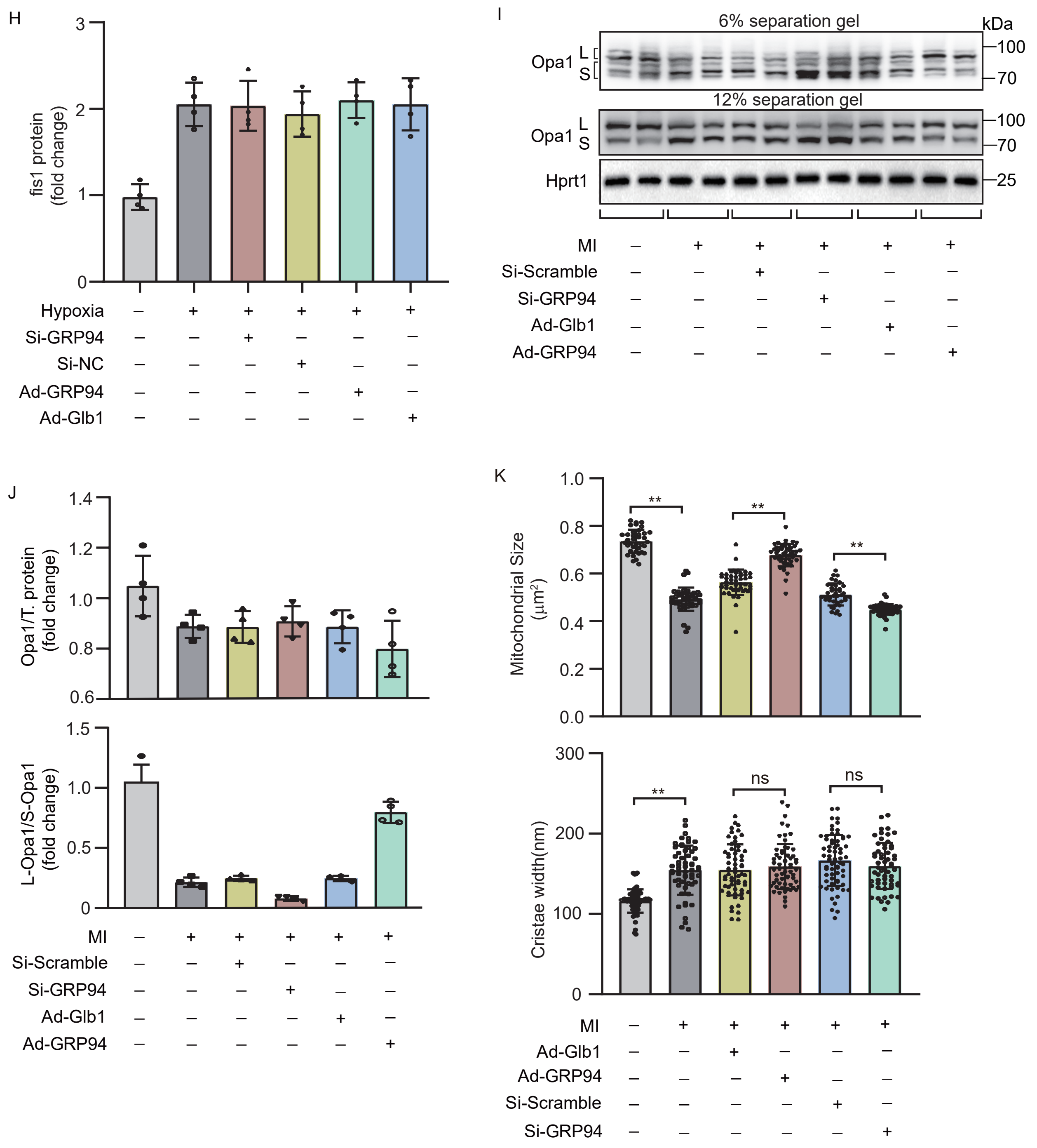

H
fis1 protein (fold change)
Hypoxia
Si-GRP94
Si-NC
Ad-GRP94
Ad-Glb1
I
6% separation gel
12% separation gel
kDa
Opa1
L
S
Hprt1
100
70
25
MI
Si-Scramble
Si-GRP94
Ad-Glb1
Ad-GRP94
J
Opa1/T. protein (fold change)
L-Opa1/S-Opa1 (fold change)
MI
Si-Scramble
Si-GRP94
Ad-Glb1
Ad-GRP94
K
Mitochondrial Size (μm²)
Cristae width(nm)
**
ns
MI
Ad-Glb1
Ad-GRP94
Si-Scramble
Si-GRP94

Supplementary Figure 2. GRP94 attenuates cardiomyocyte apoptosis and mitochondrial fragmentation caused by hypoxia stress. (**A)** Time-dependent GRP94 degradation under oxidative stress. Cardiomyocytes treated with 200 μM H_2O_2 for 0-24 hr. GRP94 levels analyzed by immunoblotting (n=4). *P<0.05 and **P<0.01 versus untreated group in one-way ANOVA followed by Tukey–Kramer post hoc analysis. (**B)** Efficient GRP94 modulation. Cardiomyocytes infected with Ad-GRP94 or Ad-Glb1 (MOI=50, 36 hr), or transfected with GRP94 siRNA/si-NC (60 nM, 36 hr), followed by hypoxia (12 hr). GRP94 expression confirmed by immunoblotting (n=4). (**C)** GRP94 rescues hypoxia-induced viability loss. Cell viability assessed by CCK-8 assay under conditions in (B). *P<0.05 and **P<0.01 versus untreated group in one-way ANOVA followed by Tukey–Kramer post hoc analysis (n=4). (**D-H)** GRP94 attenuates the cleavage of Opa1 to short (S)-Opa1 caused by hypoxia stress. Cardiomyocytes were treated as in (B). The levels of Mfn1, Mfn2, Drp1, phosphoDrp1 and Fis1 were analyzed by immunoblot. *P<0.05 and **P<0.01 versus the indicated group in one-way ANOVA followed by Tukey–Kramer post hoc analysis (n=4). All data are shown as mean ± SD. (**I-J)** GRP94 attenuates the cleavage of Opa1 to short (S)-Opa1 caused by MI. Adult male C57 mice (8–10 weeks old) were injected with Adeno-Associated Virus2/9-GRP94, Glb1, GRP94 siRNA or siRNA scramble and subjected to permanent left anterior descending coronary artery ligation. The levels of Opa1 were analyzed by immunoblot with Hprt1 as internal control (n=4). *P<0.05 and **P<0.01 in one-way ANOVA followed by Tukey–Kramer post hoc analysis. (**K**), Quantification of mean mitochondrial size (the upper). n=37 for sham group, 43 for MI, 47 for Ad-Glb1, 49 for Ad-GRP94, 41 for Si-Scramble, and 53 for Si-GRP94. Quantification of cristae width (below). n=70 for sham group, 58 for MI, 60 for Ad-Glb1, 63 for Ad-GRP94, 66 for Si-Scramble and 58 for Si-GRP94. Analysis was performed with one-way ANOVA followed by Tukey–Kramer post hoc analysis. *P<0.05; **P<0.01. ns, no significance.

Figure S3

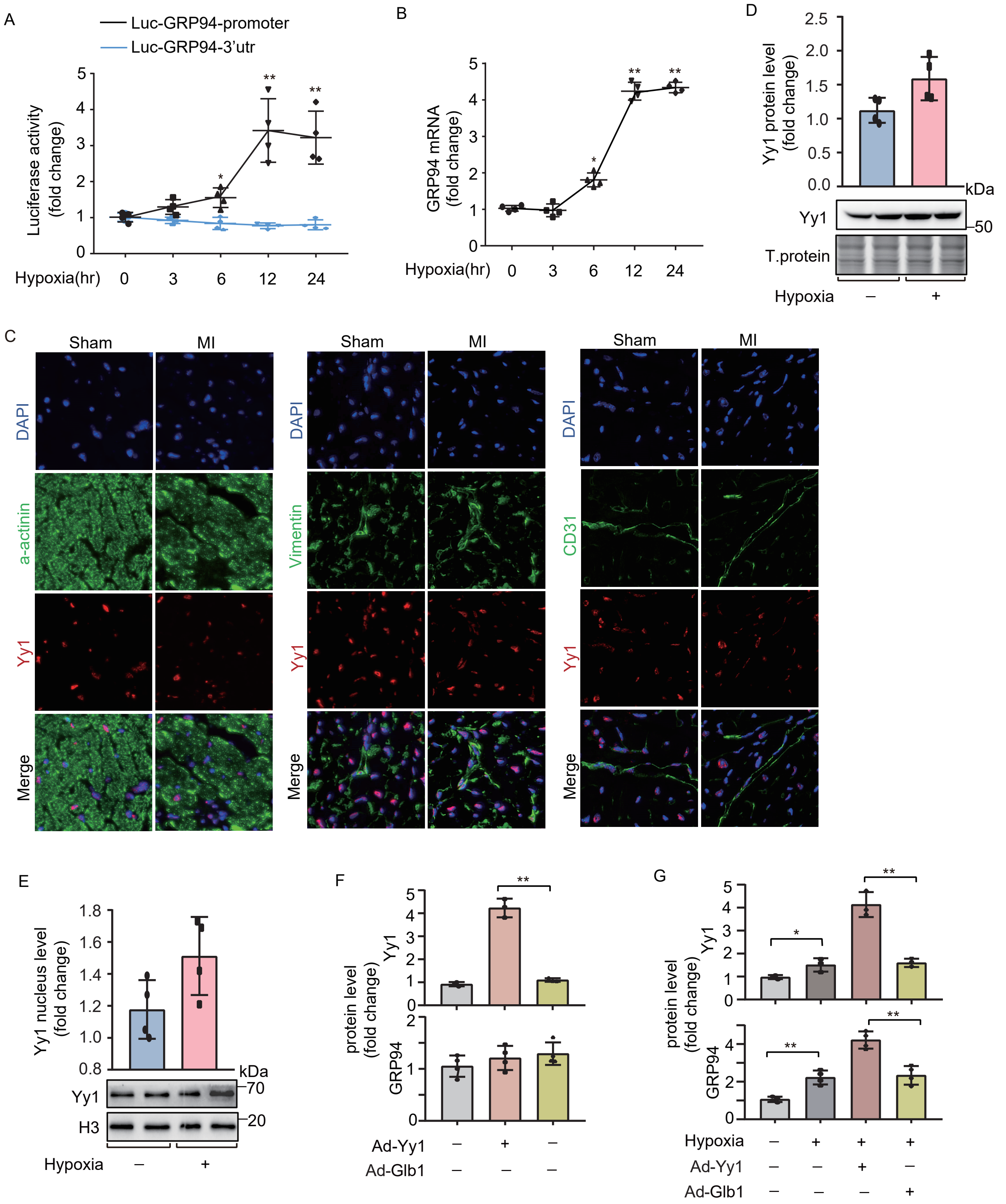
A
Luc-GRP94-promoter
Luc-GRP94-3'utr
Luciferase activity
(fold change)
Hypoxia(hr) 0 3 6 12 24
B
GRP94 mRNA
(fold change)
Hypoxia(hr) 0 3 6 12 24
D
Yy1 protein level
(fold change)
kDa
Yy1
50
T.protein
Hypoxia – +
C
Sham MI
DAPI
a-actinin
Vimentin
CD31
Yy1
Merge
E
Yy1 nucleus level
(fold change)
kDa
Yy1
70
H3
20
Hypoxia – +
F
protein level
(fold change)
Yy1
GRP94
Ad-Yy1 – + –
Ad-Glb1 – – –
G
protein level
(fold change)
Yy1
GRP94
Hypoxia – + + +
Ad-Yy1 – – + –
Ad-Glb1 – – – +

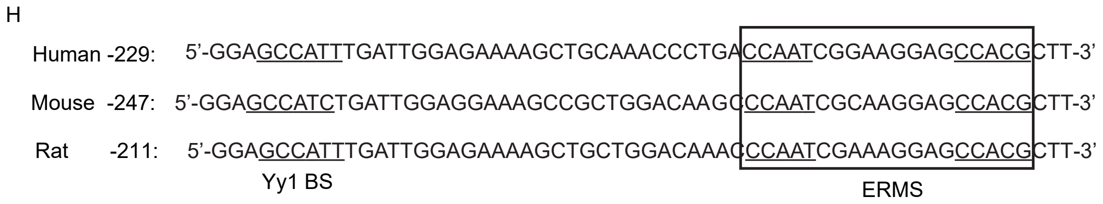
H
Human -229: 5'-GGAGCCATTTGATTGGAGAAAAGCTGCAAACCCTGACCAATCGGAAGGAGCCACGCTT-3'
Mouse -247: 5'-GGAGCCATCTGATTGGAGGAAAGCCGCTGGACAAGCCCAATCGCAAGGAGCCACGCTT-3'
Rat -211: 5'-GGAGCCATTTGATTGGAGAAAAGCTGCTGGACAAACCCAATCGAAAGGAGCCACGCTT-3'
Yy1 BS
ERMS

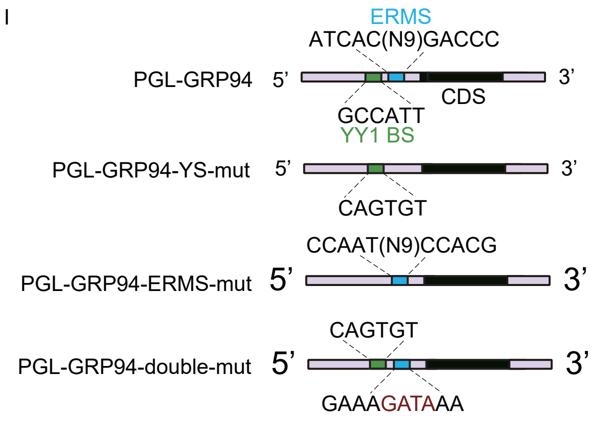
I
ERMS
ATCAC(N9)GACCC
PGL-GRP94 5' 3'
CDS
GCCATT
YY1 BS
PGL-GRP94-YS-mut 5' 3'
CAGTGT
CCAAT(N9)CCACG
PGL-GRP94-ERMS-mut 5' 3'
CAGTGT
PGL-GRP94-double-mut 5' 3'
GAAAGATAAA

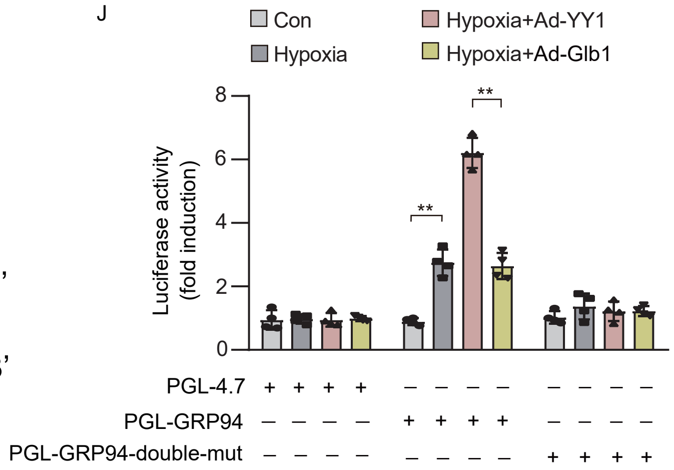
J
Con
Hypoxia
Hypoxia+Ad-YY1
Hypoxia+Ad-Glb1
Luciferase activity (fold induction)
0
2
4
6
8
**
**
PGL-4.7 + + + + – – – – – – – –
PGL-GRP94 – – – – + + + + – – – –
PGL-GRP94-double-mut – – – – – – – – + + + +

Supplementary Figure 3. Yy1 transcriptionally regulates GRP94 under hypoxia through conserved promoter elements. (**A**) Hypoxia enhances GRP94 promoter activity. MCM cell lines were transfected with the constructs of the pGL-4.17 empty vector (pGL-4.17) or the pGL-4.17 vector harboring GRP94 promoter (pGL-GRP94) at 1ng/ml. After 24h, cells were cultivated for another 12h under hypoxic conditions. Luciferase activity was assayed. *P<0.05 and **P<0.01 versus the indicated group in one-way ANOVA followed by Tukey–Kramer post hoc analysis (n=4). (**B**) Hypoxia induces an increase of GRP94 mRNA. Cardiomyocytes under hypoxia (0-24 hr). GRP94 mRNA quantified by qRT-PCR. *P<0.05 and **P<0.01 versus the indicated group in one-way ANOVA followed by Tukey–Kramer post hoc analysis (n=4). (**C**) Immunofluorescence co-staining analysis of Yy1 with cardiomyocyte marker α-actinin, fibroblast marker Vimentin, and endothelial marker CD31 in mouse heart sections (scale bar: 40 μm). (**D**) Hypoxia has no significant effect on Yy1 protein level. Cardiomyocytes were cultivated under hypoxia for 12 hr. Yy1 protein was then analyzed by immunoblot (n=4). Analysis is performed with an unpaired two-tailed Student's t-test. (**E**) Hypoxia has no significant effect on Yy1 nuclear localization. Cardiomyocytes were cultivated under hypoxia for 12 hr. Nucleuses were isolated, and Yy1 protein was then analyzed by immunoblot (n=4). Analysis is performed with an unpaired two-tailed Student's t-test. (**F-G**) AI662270 or Oip5os1 does not affect Yy1 nuclear content. Cardiomyocytes were infected with Ad-Oip5os1 (MOI=40) or transfected with ASO-AI662270 (40 nM, 36 hr) prior to hypoxia. Nuclear fractionation was isolated, and Yy1 protein was then analyzed by immunoblot. *P<0.05 and **P<0.01 versus the indicated group in one-way ANOVA followed by Tukey–Kramer post hoc analysis (n=4). (**H**) Alignment analysis of the mouse, rat and human GRP94 promoter pairs. The conserved ERSE motif (boxed) and adjacent Yy1 binding site (underlined) are indicated in bold letters with an underline. (**I**) Mutated GRP94 promoter in the Yy1 binding site and/or ERMS are shown. (**J**) Dual mutation of ERMS/Yy1 sites abolishes Yy1-mediated promoter activation. MCM were transfected with the constructs of the pGL-4.17 empty vector (pGL-4.17), the pGL-4.17 vector harboring GRP94 promoter (pGL-GRP94) or the promoter with dual mutations in the ERMS and Yy1 binding site (pGL-GRP94-double-mut) respectively at 1ng/ml and then infected with the adenoviral Glb1 or Yy1 at 40 MOIs. After 24h, cells were cultivated for 12h under hypoxic conditions. Luciferase activity was assayed. *P<0.05 and **P<0.01 versus the indicated group in one-way ANOVA followed by Tukey–Kramer post hoc analysis (n=4). All data are shown as mean ± SD.

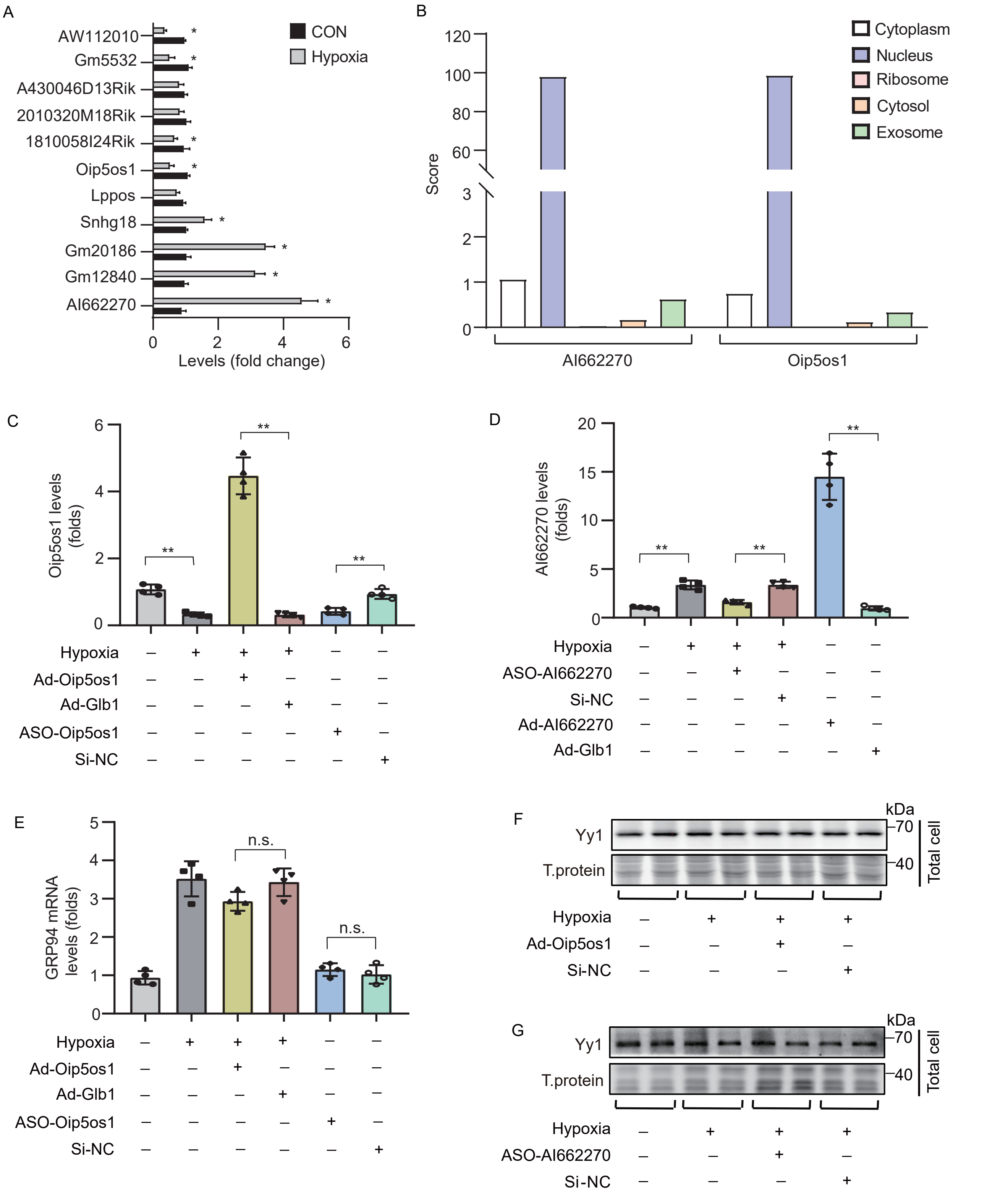

A
AW112010
Gm5532
A430046D13Rik
2010320M18Rik
1810058I24Rik
Oip5os1
Lppos
Snhg18
Gm20186
Gm12840
AI662270
CON
Hypoxia
Levels (fold change)
B
Score
Cytoplasm
Nucleus
Ribosome
Cytosol
Exosome
AI662270
Oip5os1
C
Oip5os1 levels (folds)
Hypoxia
Ad-Oip5os1
Ad-Glb1
ASO-Oip5os1
Si-NC
D
AI662270 levels (folds)
Hypoxia
ASO-AI662270
Si-NC
Ad-AI662270
Ad-Glb1
E
GRP94 mRNA levels (folds)
n.s.
Hypoxia
Ad-Oip5os1
Ad-Glb1
ASO-Oip5os1
Si-NC
F
Yy1
T.protein
kDa
70
40
Total cell
Hypoxia
Ad-Oip5os1
Si-NC
G
Yy1
T.protein
kDa
70
40
Total cell
Hypoxia
ASO-AI662270
Si-NC

Supplementary Figure 4. LncRNA-AI662270 interacts with Yy1 to promote GRP94 transcription. (**A)** The differentially expressed LncRNAs in the mouse model post-MI were confirmed by qRT-PCR. Cardiomyocytes were cultivated for 12h under hypoxic conditions. *P<0.05 and **P<0.01 versus the control group in Student's t-test (n=3). (**B)** Subcellular localization prediction of AI662270 and Oip5os1. (**C**) Oip5os1 modulation in cardiomyocytes. Adenoviral overexpression (Ad-Oip5os1 vs. Ad-Glb1 at MOI 40) or ASO-mediated knockdown (40 nM ASO-Oip5os1 vs. ASO-NC) followed by 12 h hypoxia. qRT-PCR quantification (n=4). *P<0.05 and **P<0.01 versus the indicated group in one-way ANOVA followed by Tukey–Kramer post hoc analysis (n=4). (**D)** AI662270 modulation using adenoviral overexpression (Ad-AI662270) or ASO knockdown under identical experimental conditions as panel C. (**E)** Lnc-Oip5os1 shows no effect on the mRNA level of GRP94. Experimental setup as panel C. The mRNA of GRP94 was then analyzed by qRT-PCR. *P<0.05 and **P<0.01 versus the indicated group in one-way ANOVA followed by Tukey–Kramer post hoc analysis (n=4). (**F-G)** AI662270 or Oip5os1 does not affect the total Yy1 content. Cardiomyocytes were infected with adenoviruses overexpressing Oip5os1 (Ad-Oip5os1) or Glb1 (Ad-Glb1) at MOIs of 40, or transfected with 40nM ASO-AI662270 or ASO-NC. After 36h, cells were cultivated for 12h under hypoxic conditions. Yy1 protein was then analyzed by immunoblot. All data are shown as mean ± SD.

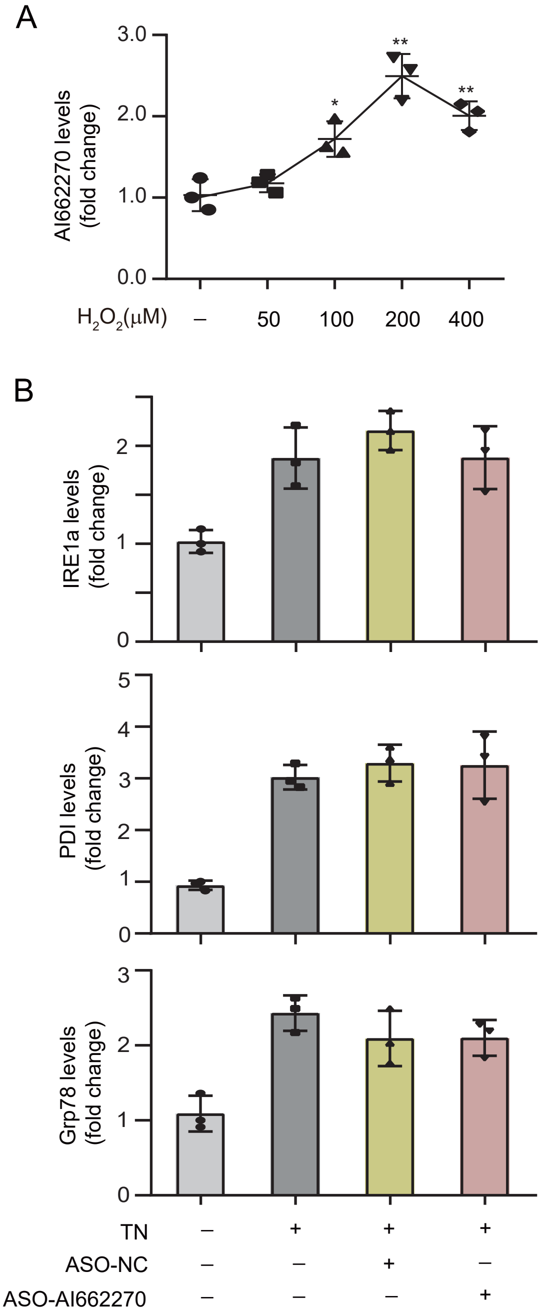
A
3.0
2.0
1.0
0.0
AI662270 levels
(fold change)
*
**
**
H_2O_2(μM)
–
50
100
200
400
B
IRE1a levels
(fold change)
PDI levels
(fold change)
Grp78 levels
(fold change)
TN
ASO-NC
ASO-AI662270

Supplementary Figure 5. ER stress-induced LncRNA-AI662270 exerts GRP94-like functional effects in vitro. (**A)** H_2O_2 treatment induces an increase in AI662270 levels. Cardiomyocytes were treated with the indicated dose of H_2O_2 for 12h. AI662270 levels were analyzed by qRT-PCR (n=3). Analysis was performed with one-way ANOVA followed by Tukey–Kramer post hoc analysis. *P<0.05, **P<0.01. (**B**) AI662270 knockdown attenuates ER stress signaling. Cardiomyocytes transfected with 40 nM ASO-AI662270 or ASO-NC for 24 h, followed by tunicamycin (TN, 15 mg/L) challenge for 12 h. Immunoblot analysis of ER stress markers (IRE1α, PDI, GRP78). Analysis was performed with one-way ANOVA followed by Tukey–Kramer post hoc analysis (n=3). All data are shown as mean ± SD.

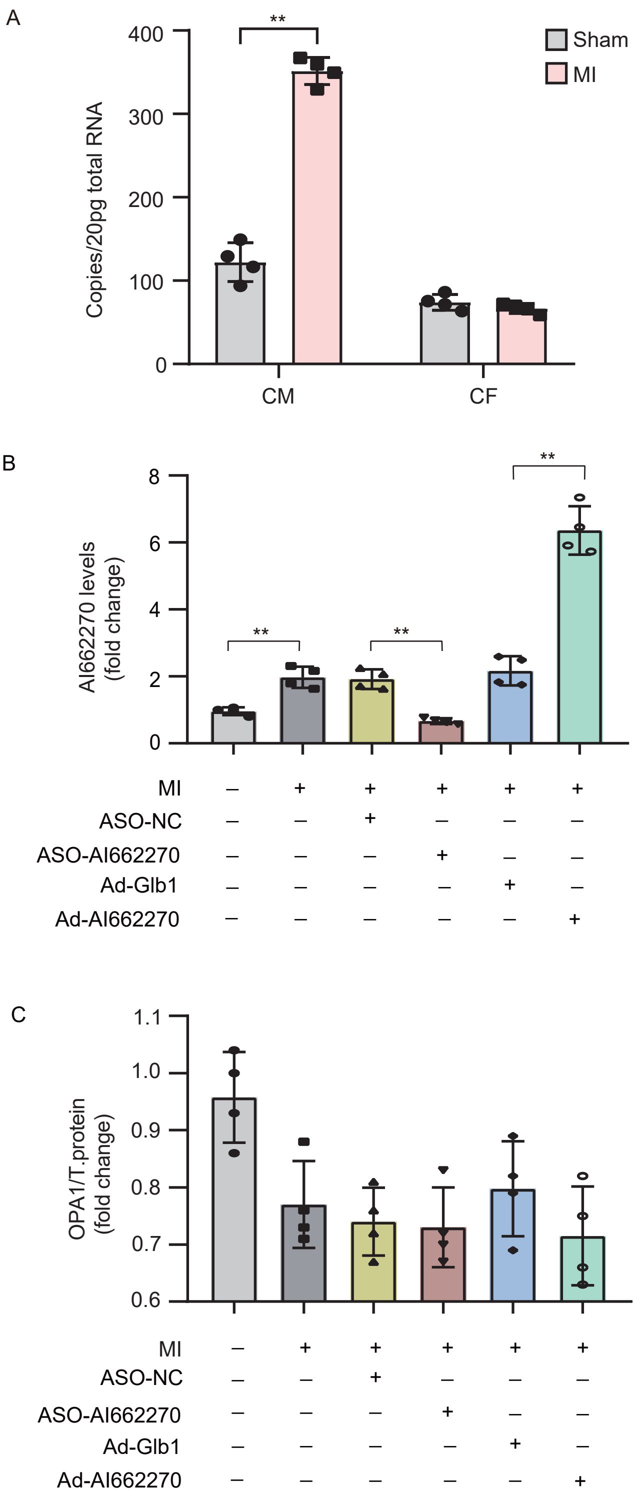
A
**
Sham
MI
Copies/20pg total RNA
400
300
200
100
0
CM
CF
B
**
**
**
AI662270 levels
(fold change)
8
6
4
2
0
MI – + + + + +
ASO-NC – – + – – –
ASO-AI662270 – – – + – –
Ad-Glb1 – – – – + –
Ad-AI662270 – – – – – +
C
OPA1/T.protein
(fold change)
1.1
1.0
0.9
0.8
0.7
0.6
MI – + + + + +
ASO-NC – – + – – –
ASO-AI662270 – – – + – –
Ad-Glb1 – – – – + –
Ad-AI662270 – – – – – +

Supplementary Figure 6. Lnc-AI662270 exerts cardioprotective effects post-MI. (**A**) Absolute quantitative real-time PCR analysis of AI662270 expressions in cardiomyocytes and cardiac fibroblasts isolated from Sham and MI groups (n=4). *P<0.05 and **P<0.01 versus the indicated group in one-way ANOVA followed by Tukey–Kramer post hoc analysis. (**B)** Manipulation of AI662270 levels in the mouse model. Adult male C57 mice (8–10 weeks old) were injected with Adeno-Associated Virus2/9-AI662270, Glb1, ASO-AI662270, or ASO-NC immediately after permanent left anterior descending coronary artery ligation. AI662270 levels were analyzed by qRT-PCR (n=4). (**C)** AI662270 does not influence total Opa1 protein levels. Mice were treated as in (A). Opa1 protein was analyzed by immunoblot. All data are shown as mean ± SD. *P<0.05 and **P<0.01 versus the indicated group in one-way ANOVA followed by Tukey–Kramer post hoc analysis.

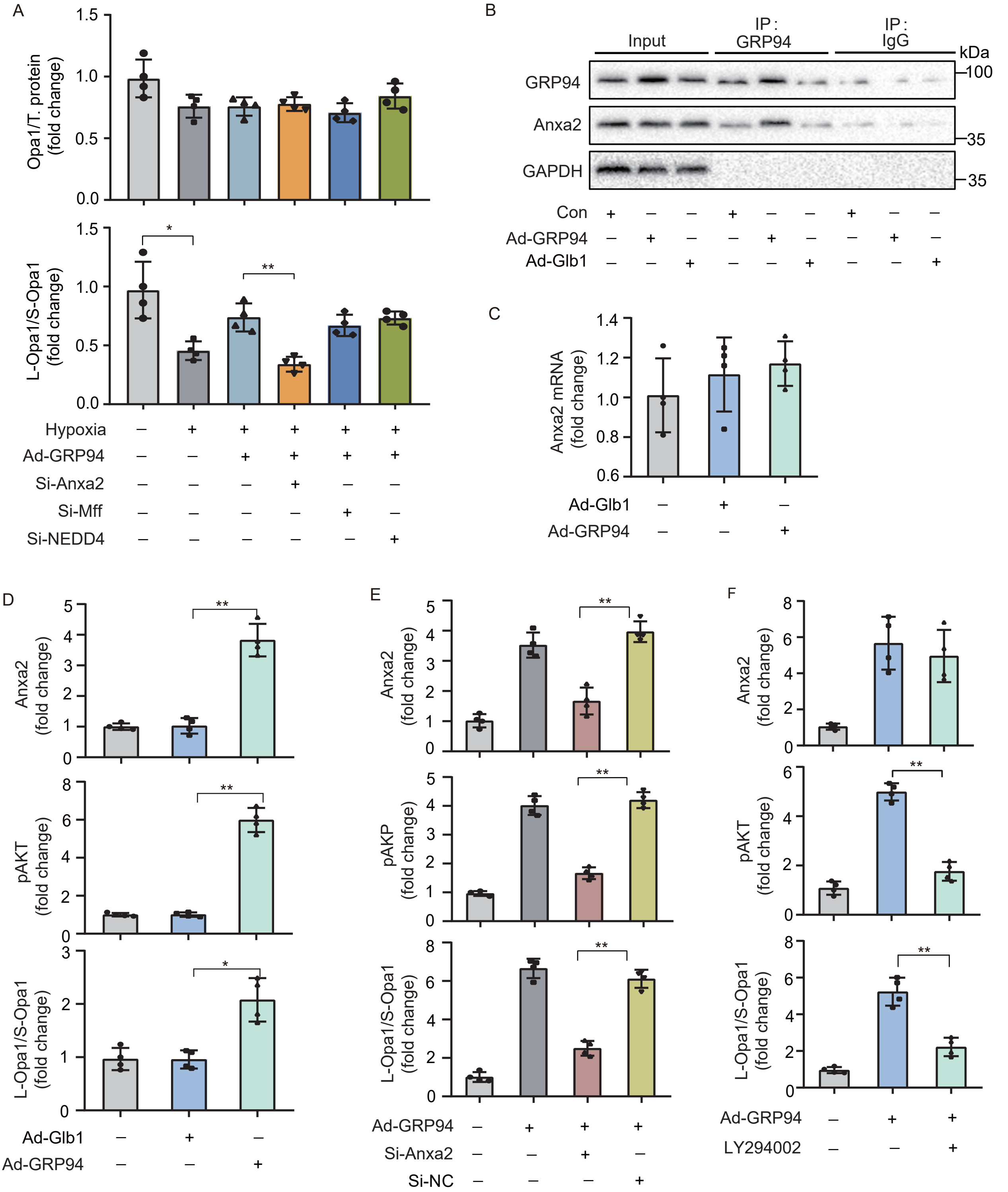
A
Opa1/T. protein (fold change)
L-Opa1/S-Opa1 (fold change)
Hypoxia
Ad-GRP94
Si-Anxa2
Si-Mff
Si-NEDD4
B
Input
IP : GRP94
IP : IgG
kDa
GRP94
Anxa2
GAPDH
100
35
35
Con
Ad-GRP94
Ad-Glb1
C
Anxa2 mRNA (fold change)
Ad-Glb1
Ad-GRP94
D
Anxa2 (fold change)
pAKT (fold change)
L-Opa1/S-Opa1 (fold change)
Ad-Glb1
Ad-GRP94
E
Anxa2 (fold change)
pAKP (fold change)
L-Opa1/S-Opa1 (fold change)
Ad-GRP94
Si-Anxa2
Si-NC
F
Anxa2 (fold change)
pAKT (fold change)
L-Opa1/S-Opa1 (fold change)
Ad-GRP94
LY294002

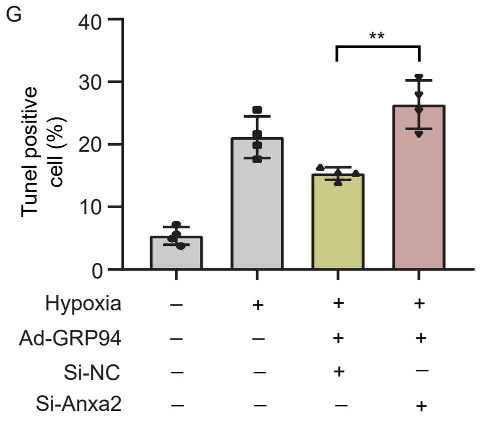
G
**
Tunel positive cell (%)
40
30
20
10
0
Hypoxia – + + +
Ad-GRP94 – – + +
Si-NC – – + –
Si-Anxa2 – – – +

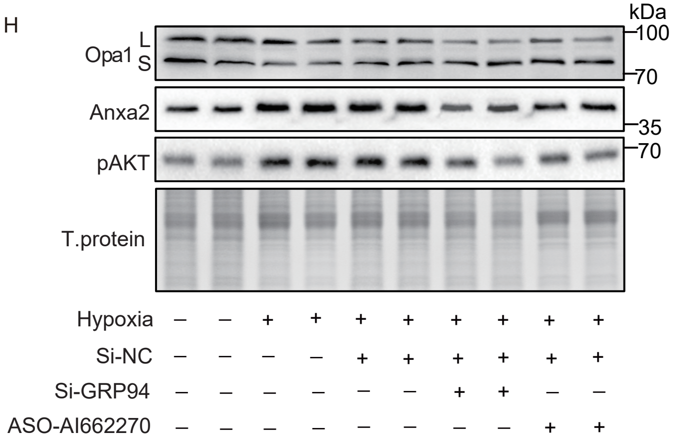
H
kDa
Opa1 L S
100
70
Anxa2
35
pAKT
70
T.protein
Hypoxia – – + + + + + + + +
Si-NC – – – – + + + + + +
Si-GRP94 – – – – – – + + – –
ASO-AI662270 – – – – – – – – + +

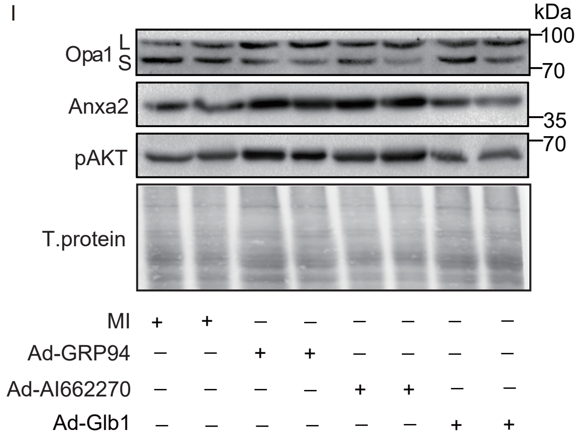
I
kDa
Opa1 L S
100
70
Anxa2
35
pAKT
70
T.protein
MI + + – – – – – –
Ad-GRP94 – – + + – – – –
Ad-AI662270 – – – – + + – –
Ad-Glb1 – – – – – – + +

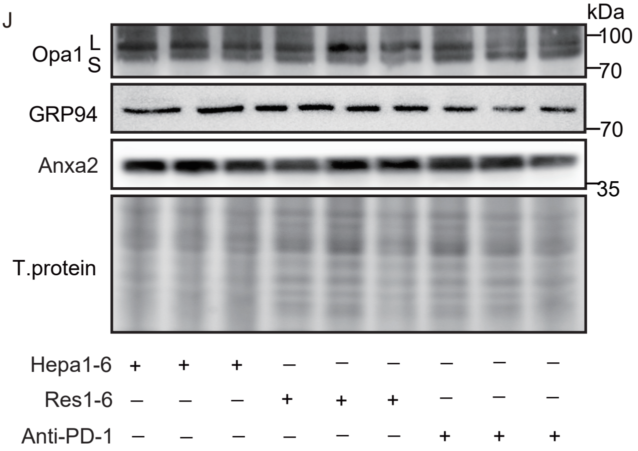
J
kDa
Opa1 L S
100
70
GRP94
70
Anxa2
35
T.protein
Hepa1-6 + + + – – – – – –
Res1-6 – – – + + + – – –
Anti-PD-1 – – – – – – + + +

Supplementary Figure 7. GRP94 interacts with Anxa2 to modulate Opa1 proteolytic processing. (**A**) Anxa2 ablation abrogates GRP94-mediated effects on Opa1 processing. Cardiomyocytes were co-transfected with 40 nM siRNA targeting Anxa2 (Si-Anxa2), Mff (Si-Mff), NEDD4 (Si-Nedd4), or siRNA scramble (Si-NC), followed by adenoviral infection (MOI=50) expressing GRP94 (Ad-GRP94) or β-galactosidase (Ad-Glb1). After 36 h, cells were exposed to hypoxia (1% O2) for 12 h. Opa1 protein was analyzed by immunoblot. (**B**) Endogenous GRP94-Anxa2 interaction. Co-immunoprecipitation (Co-IP) using GRP94 antibody or IgG control in cardiomyocytes treated with 15 mg/L tunicamycin for 12 h. (**C**) GRP94 overexpression does not alter Anxa2 transcript levels. qRT-PCR analysis 48 h post-Ad-GRP94/Ad-Glb1 infection (n=4). (**D**) GRP94 enhances Anxa2 protein stabilization. Western blot analysis of Anxa2, p-AKT (Ser473), and Opa1 isoforms after 48 hr adenovirus overexpressing GRP94 (Ad-GRP94) or Glb1 (Ad-Glb1) at MOIs of 50 infection (n=4). (**E**) Anxa2 knockdown reverses GRP94-induced AKT phosphorylation and Opa1 processing. Immunoblot analysis following Ad-GRP94 infection (MOI=50) with Si-Anxa2/Si-NC (40nM). (**F**) AKT inhibition blocks GRP94-mediated effects. LY294002 (50 μM, 48h) treatment with Ad-GRP94 infection (MOI=50). (**G**) Anxa2 deletion abolishes the protective effect of GRP94 on hypoxia-induced apoptosis. TUNEL assay quantification (n=4). (**H**) AI662270 knockdown represses hypoxia-induced Anxa2 as GRP94 deletion did. Western blot analysis after 60 nM Si-GRP94 or 40 nM ASO-AI662270 transfection with 12h hypoxia (n=3). (**I**) AI662270 augments the levels of L-Opa1, Anxa2, and phosphorylated AKT in the mouse model as GRP94 does. C57BL/6 mice (8-10 weeks) received AAV2/9-GRP94, AAV2/9-AI662270, or AAV2/9-Glb1 immediately after permanent LAD ligation. Opa1, Anxa2, and phosphorylated AKT protein were analyzed by immunoblot (n=3). (**J**) Anti-PD-1 treatment decreases the cardiac level of GRP94/L-Opa1 signal. For metastasis challenge experiments, mice were injected with Hepa1-6 cells or drug-resistant Hepa1-6 cells (termed as Res1-6). After tumor formation, mice were treated with 200 μg of PD-1 antibody (anti-PD-1) three times a week for two weeks. Cardiac protein levels analyzed by western blot (n=3). $^*P<0.05$ and $^{**}P<0.01$ versus the indicated group in one-way ANOVA followed by Tukey–Kramer post hoc analysis. All data are shown as mean ± SD.

Supplementary Table 1: RNA deep sequencing results revealed the extensive alteration of cardiac ER stress-response genes at 1d and 7d post MI in mice model.

Official_Symbol	Gene type	Mean_Sham	Mean_MI1d	Mean_MI7d	Folds (MI1d/Sham)	Folds (MI7d/Sham)	pValue (MI1d/Sham)	pValue (MI1d/Sham)	pValue (MI7d/MI1d)
**Ddit3**	protein_coding	43.82	14.20	16.43	0.32	0.38	0.08	0.09	0.02
**Ppp1r15a**	protein_coding	14.80	10.27	10.06	0.69	0.68	0.03	0.05	0.91
**Atf6**	protein_coding	23.43	22.54	16.69	0.96	0.71	0.62	0.02	0.01
**Atf4**	protein_coding	40.59	40.88	35.40	1.01	0.87	0.97	0.44	0.40
**Atf3**	protein_coding	6.69	7.83	4.25	1.17	0.63	0.65	0.11	0.14
**Ern2**	protein_coding	4.13	4.33	4.35	1.05	1.05	0.49	0.48	0.97
**Hspa5**	protein_coding	23.15	35.98	25.88	1.55	1.12	0.01	0.23	0.03
**Canx**	protein_coding	3.26	3.39	3.02	1.04	0.93	0.78	0.55	0.44
**Ero1l**	protein_coding	7.21	9.42	6.70	1.31	0.93	0.12	0.42	0.06
**Xbp1**	protein_coding	1.88	2.67	2.02	1.42	1.08	0.03	0.54	0.01
**Erp44**	protein_coding	17.38	21.68	17.21	1.25	0.99	0.00	0.86	0.01
**Hsp90b1**	protein_coding	231.31	384.54	228.20	1.66	0.99	0.00	0.76	0.00
**Calr**	protein_coding	201.44	358.78	213.59	1.78	1.06	0.00	0.44	0.00
**Edem1**	protein_coding	6.07	12.28	11.00	2.02	1.81	0.00	0.03	0.47
**Dnajc3**	protein_coding	17.54	28.02	22.40	1.60	1.28	0.00	0.09	0.08
**Pdia3**	protein_coding	89.04	188.07	126.44	2.11	1.42	0.00	0.02	0.01
**Pdia4**	protein_coding	24.59	64.15	39.15	2.61	1.59	0.00	0.00	0.00

Supplementary Table 2: The differently expressed LncRNAs in left ventricular myocardium, with the multiple of expression level change less than 0.7 or more than 1.5 and p value less than 0.05, at 1d and 7d post MI in mice model.

Official_Symbol	Gene type	Mean_Sham	Mean_MI1d	Mean_MI7d	Folds (MI1d/Sham)	Folds (MI7d/Sham)	pValue (MI1d/Sham)	pValue (MI1d/Sham)2	pValue (MI7d/MI1d)
**AI662270**	lincRNA	3.21	11.74	5.00	3.66	1.56	0.00	0.26	0.02
**Gm12840**	lincRNA	131.93	319.09	264.96	2.42	2.01	0.00	0.00	0.11
**Gm20186**	lincRNA	17.22	36.27	36.79	2.11	2.14	0.03	0.00	0.93
**Hist2h2bb**	lincRNA	3.38	6.83	6.69	2.02	1.98	0.00	0.08	0.92
**Gm26857**	lincRNA	2.58	4.89	3.17	1.90	1.23	0.01	0.07	0.03
**Gm26882**	lincRNA	1.43	2.46	1.89	1.72	1.33	0.00	0.16	0.14
**Lppos**	lincRNA	4.59	3.17	3.79	0.69	0.83	0.01	0.08	0.06
**1700020I14Rik**	lincRNA	55.12	37.36	37.96	0.68	0.69	0.02	0.02	0.84
**1810058I24Rik**	lincRNA	10.71	7.22	8.82	0.67	0.82	0.00	0.03	0.03
**Gm26899**	lincRNA	117.34	78.62	66.75	0.67	0.57	0.05	0.03	0.31
**2010320M18Rik**	lincRNA	8.60	5.64	7.20	0.66	0.84	0.01	0.15	0.04
**Gm14290**	lincRNA	7.81	4.97	5.09	0.64	0.65	0.03	0.06	0.88
**Gdap10**	lincRNA	3.75	2.37	2.00	0.63	0.53	0.05	0.03	0.34
**A430046D13Rik**	lincRNA	5.64	3.55	3.06	0.63	0.54	0.02	0.01	0.49
**Gm6712**	lincRNA	4.83	2.55	4.45	0.53	0.92	0.02	0.38	0.02
**Yam1**	lincRNA	35.11	17.51	107.25	0.50	3.05	0.00	0.51	0.42
**RP23-281H4.12**	lincRNA	180.51	89.32	88.44	0.49	0.49	0.01	0.01	0.96
**Gm5532**	lincRNA	40.21	19.88	23.44	0.49	0.58	0.00	0.02	0.43
**AW112010**	lincRNA	40.89	19.48	24.24	0.48	0.59	0.00	0.00	0.20

Supplementary Table 3: The proteins potentially interacts with GRP94 in cardiomyocytes under hypoxia treatment.

Protein_ID	GeneName	log2fc	Regulation	HYP-GRP94	HYP-IgG
P07356	Anxa2	5.436189495	up	9.74128123	4.305091738
A0A1B0GSX0	Ldha	5.331487538	up	9.63657928	4.305091738
P68033	Actc1	4.992360727	up	9.29745246	4.305091738
E9Q8K5	Ttn	4.929759929	up	9.23485167	4.305091738
A0A1W2P6F6	Myl6	4.922165998	up	9.22725774	4.305091738
A0A1B0GQU8	Rpl18	4.811368491	up	9.11646023	4.305091738
P08249	Mdh2	4.771863326	up	9.07695506	4.305091738
P63028	Tpt1	4.747389906	up	9.05248164	4.305091738
P11276	Fn1	4.662475835	up	8.96756757	4.305091738
Q91YL2	Rnf126	4.637627737	up	8.94271948	4.305091738
A0A1B0GS70	Psma1	4.615023121	up	8.92011486	4.305091738
F8WIV2	Serpinb6a	4.587915421	up	8.89300716	4.305091738
E9PZF5	Anp32e	4.560385834	up	8.86547757	4.305091738
Q6ZWQ9	Myl12a	4.50265482	up	8.80774656	4.305091738
P40124	Cap1	4.480871907	up	8.78596365	4.305091738
Q6ZQ38	Cand1	4.446803336	up	8.75189507	4.305091738
Q9R0P3	Esd	4.445703302	up	8.75079504	4.305091738
B1ARA3	Rpl26	4.399292415	up	8.70438415	4.305091738
F6ZFU0	Eef1d	4.383388285	up	8.68848002	4.305091738
P63158	Hmgb1	4.363272231	up	8.66836397	4.305091738
D3Z1S8	Rps5	4.352111215	up	8.65720295	4.305091738
A0A0R4J0Z1	Pdia4	4.339774163	up	8.6448659	4.305091738
P08228	Sod1	4.316476221	up	8.62156796	4.305091738
P47738	Aldh2	4.279694908	up	8.58478665	4.305091738
Q61171	Prdx2	4.238221949	up	8.54331369	4.305091738
P80317	Cct6a	4.222725686	up	8.52781742	4.305091738
P61205	Arf3	4.212936355	up	8.51802809	4.305091738
P08207	S100a10	4.200380859	up	8.5054726	4.305091738
P24472	Gsta4	4.16561957	up	8.47071131	4.305091738
Q93092	Taldo1	4.147645236	up	8.45273697	4.305091738
Q99K85	Psat1	4.143443361	up	8.4485351	4.305091738
P10639	Txn	4.137247746	up	8.44233948	4.305091738
Q8K310	Matr3	4.133595344	up	8.43868708	4.305091738
P97429	Anxa4	4.129184537	up	8.43427628	4.305091738
A0A1W2P8C6	Cd63	4.122567091	up	8.42765883	4.305091738
E9Q905	Ctnnd1	4.11968665	up	8.42477839	4.305091738
Q8BMD8	Slc25a24	4.113473925	up	8.41856566	4.305091738
P51410	Rpl9	4.088021194	up	8.39311293	4.305091738
Q80X68	Csl	4.065420076	up	8.37051181	4.305091738
Q8C483	Sars	4.062937154	up	8.36802889	4.305091738

O09131	Gsto1	4.057623752	up	8.36271549	4.305091738
Q7TMK9	Syncrip	4.025631797	up	8.33072354	4.305091738
Q9CWJ9	Atic	4.012488517	up	8.31758025	4.305091738
Q9CZN7	Shmt2	3.997597222	up	8.30268896	4.305091738
Q61990	Pcbp2	3.995372603	up	8.30046434	4.305091738
P50247	Ahcy	3.986224333	up	8.29131607	4.305091738
Q6PCP5	Mff	3.977588752	up	8.28268049	4.305091738
Q9CR16	Ppid	3.961938234	up	8.26702997	4.305091738
K3W4Q8	Bsg	3.943551077	up	8.24864282	4.305091738
Q61598	Gdi2	3.938508686	up	8.24360042	4.305091738
F6QA74	Apex1	3.913736582	up	8.21882832	4.305091738
F8WJK8	St13	3.893325738	up	8.19841748	4.305091738
P23927	Cryab	3.892335015	up	8.19742675	4.305091738
P45376	Akr1b1	3.882427288	up	8.18751903	4.305091738
Q61553	Fscn1	3.874950199	up	8.18004194	4.305091738
A2AMW0	Capzb	3.866268735	up	8.17136047	4.305091738
V9GX81	Mroh6	3.843368817	up	8.14846055	4.305091738
B2RY53	Gm6133	3.829561492	up	8.13465323	4.305091738
P97315	Csrp1	3.825566599	up	8.13065834	4.305091738
Q9DBG3	Ap2b1	3.819205301	up	8.12429704	4.305091738
Q6IFZ6	Krt77	3.81042367	up	8.11551541	4.305091738
P70349	Hint1	3.802243051	up	8.10733479	4.305091738
P80316	Cct5	3.794591548	up	8.09968329	4.305091738
Q61792	Lasp1	3.787584146	up	8.09267588	4.305091738
P10605	Ctsb	3.784783049	up	8.08987479	4.305091738
B9EKI3	Tmf1	3.775989476	up	8.08108121	4.305091738
Q9QYB1	Clic4	3.768949105	up	8.07404084	4.305091738
O35129	Phb2	3.765745817	up	8.07083756	4.305091738
Q9JJI8	Rpl38	3.761700973	up	8.06679271	4.305091738
A0A498WFS2	Ubxn1	3.757121132	up	8.06221287	4.305091738
Q9D0R2	Tars1	3.755288191	up	8.06037993	4.305091738
Q3V3R1	Mthfd1l	3.742138137	up	8.04722987	4.305091738
P62334	Psmc6	3.74065446	up	8.0457462	4.305091738
P26443	Glud1	3.737832746	up	8.04292448	4.305091738
Q3UZG4	Aimp1	3.730994501	up	8.03608624	4.305091738
P68510	Ywhah	3.726293486	up	8.03138522	4.305091738
Q9R0N0	Galk1	3.715391889	up	8.02048363	4.305091738
P19157	Gstp1	3.706015202	up	8.01110694	4.305091738
P35564	Canx	3.699229301	up	8.00432104	4.305091738
A0A338P7H5	Ahsg	3.698068617	up	8.00316036	4.305091738
Q99LC5	Etfa	3.68930585	up	7.99439759	4.305091738
Q8VDM4	Psmd2	3.677461319	up	7.98255306	4.305091738
Q3TLP8	Rac1	3.67081021	up	7.97590195	4.305091738
Q91V41	Rab14	3.660687057	up	7.9657788	4.305091738

Q9Z2U0	Psma7	3.63419426	up	7.939286	4.305091738
Q9D3D9	Atp5f1d	3.62582762	up	7.93091936	4.305091738
Q9D0F9	Pgm1	3.625353744	up	7.93044548	4.305091738
Q8BKC5	Ipo5	3.6005204	up	7.90561214	4.305091738
P05202	Got2	3.59324538	up	7.89833712	4.305091738
D3Z2H9	Tpm3-rs7	3.589956565	up	7.8950483	4.305091738
Q9Z0J0	Npc2	3.572031281	up	7.87712302	4.305091738
B9EHJ3	Tjp1	3.567004236	up	7.87209597	4.305091738
Q91VB8	Hba-a1	3.565853694	up	7.87094543	4.305091738
A0A0G2JGY8	Rpl34	3.560374384	up	7.86546612	4.305091738
P30416	Fkbp4	3.540267086	up	7.84535882	4.305091738
P26645	Marcks	3.533377424	up	7.83846916	4.305091738
B7ZNL2	Nap1l4	3.529175206	up	7.83426694	4.305091738
K3W4L3	Psap	3.506472998	up	7.81156474	4.305091738
A0A384DV79	Hmga1	3.488140243	up	7.79323198	4.305091738
Q6GT24	Prdx6	3.481423445	up	7.78651518	4.305091738
P17427	Ap2a2	3.466925094	up	7.77201683	4.305091738
Q6PFA2	Clta	3.464678711	up	7.76977045	4.305091738
O55135	Eif6	3.462639692	up	7.76773143	4.305091738
Q9WV55	Vapa	3.456330995	up	7.76142273	4.305091738
P30681	Hmgb2	3.452690788	up	7.75778253	4.305091738
Q3TPM3	Cul1	3.44528768	up	7.75037942	4.305091738
Q00612	G6pdx	3.433806653	up	7.73889839	4.305091738
A0A0R4J083	Acadl	3.430762894	up	7.73585463	4.305091738
Q3TW96	Uap1l1	3.398776148	up	7.70386789	4.305091738
A2A816	Park7	3.365692114	up	7.67078385	4.305091738
Q05816	Fabp5	3.363254299	up	7.66834604	4.305091738
G3UVV4	Hk1	3.342141565	up	7.6472333	4.305091738
Q5NC80	Nme1	3.340612646	up	7.64570438	4.305091738
Q91YQ5	Rpn1	3.334541029	up	7.63963277	4.305091738
A0A1W2P7Z3	Ube2n	3.317890471	up	7.62298221	4.305091738
P47226	Tes	3.304624824	up	7.60971656	4.305091738
A2AAW9	Eif2s3x	3.296719639	up	7.60181138	4.305091738
Q9Z2U1	Psma5	3.286808724	up	7.59190046	4.305091738
A0A1D5RLV0	Iws1	3.284289248	up	7.58938099	4.305091738
Q9DBG5	Plin3	3.280608907	up	7.58570064	4.305091738
P99029	Prdx5	3.270450146	up	7.57554188	4.305091738
A0A286YCD7	Adk	3.25790438	up	7.56299612	4.305091738
P17047	Lamp2	3.248369264	up	7.553461	4.305091738
A2AEX6	Fhl1	3.235342103	up	7.54043384	4.305091738
P58774	Tpm2	3.219518965	up	7.5246107	4.305091738
P47739	Aldh3a1	3.190125876	up	7.49521761	4.305091738
O88685	Psmc3	3.188530344	up	7.49362208	4.305091738
Q9CZ13	Uqcrc1	3.18188277	up	7.48697451	4.305091738

Q9ERD7	Tubb3	3.17506693	up	7.48015867	4.305091738
Q9D8B3	Chmp4b	3.088436971	up	7.39352871	4.305091738
O08579	Emd	3.082922166	up	7.3880139	4.305091738
P09055	Itgb1	3.064727143	up	7.36981888	4.305091738
Q8R5L1	C1qbp	3.014044158	up	7.3191359	4.305091738
P34022	Ranbp1	1.530795502	up	9.66544873	8.13465323
Q68FD5	Cltc	1.50820031	up	9.20371017	7.695509864
P17742	Ppia	1.210432408	up	9.43751662	8.227084211
Q9D8N0	Eef1g	1.205117283	up	9.13182836	7.926711075
Q61316	Hspa4	1.174333723	up	8.69894443	7.524610703
P14069	S100a6	1.171042745	up	9.32691715	8.155874402
P46935	Nedd4	1.117775008	up	9.75464308	8.63686807
P70168	Kpnb1	1.06905163	up	9.10513787	8.036086239
Q64727	Vcl	1.021969327	up	9.06771552	8.045746198
P26039	Tln1	1.012865913	up	9.07965862	8.066792711

Supplementary Table 4: The proteins potentially interacts with GRP94 in cardiomyocytes with TN treatment.

Protein_id	Genename	log2fc	Regulation	TN-GRP94	TN-IgG
Q6PFC0	Tbc1d16	5.33642663	up	9.35246176	4.016035134
B1ARA3	Rpl26	4.755611665	up	8.7716468	4.016035134
P09411	Pgk1	4.754569958	up	8.77060509	4.016035134
Q8K310	Matr3	4.733013627	up	8.74904876	4.016035134
F6T4M4	Srrm1	4.716784851	up	8.73281999	4.016035134
Q6IFZ6	Krt77	4.629394265	up	8.6454294	4.016035134
B9EKI3	Tmf1	4.517290356	up	8.53332549	4.016035134
P26645	Marcks	4.485012561	up	8.5010477	4.016035134
Q8BHC4	Dcakd	4.466255396	up	8.48229053	4.016035134
P07356	Anxa2	4.442867108	up	8.45890224	4.016035134
E9Q8D0	Dnajc21	4.437686785	up	8.45372192	4.016035134
E9PZF5	Anp32e	4.403290262	up	8.4193254	4.016035134
P63101	Ywhaz	4.39309955	up	8.40913468	4.016035134
Q91YL2	Rnf126	4.377332283	up	8.39336742	4.016035134
D3Z2H2	Ctnnd1	4.285636001	up	8.30167114	4.016035134
O89086	Rbm3	4.266118831	up	8.28215397	4.016035134
P06327	Gm5629	4.233707314	up	8.24974245	4.016035134
A6ZI44	Aldoa	4.230316084	up	8.24635122	4.016035134
Q7SIG6	Asap2	4.122955728	up	8.13899086	4.016035134
Q3TML0	Pdia6	4.066390077	up	8.08242521	4.016035134
Q6PCP5	Mff	4.028734267	up	8.0447694	4.016035134
P62259	Ywhae	3.986889389	up	8.00292452	4.016035134
Q9D1E6	Tbcb	3.904700852	up	7.92073599	4.016035134
P28033	Cebpb	3.891917832	up	7.90795297	4.016035134
Q3UMU9	Hdgfl2	3.879585165	up	7.8956203	4.016035134
P04104	Krt1	3.82970624	up	7.84574137	4.016035134
Q5NCC2	Trim41	3.814175288	up	7.83021042	4.016035134
Q8VE37	Rcc1	3.807877273	up	7.82391241	4.016035134
Q64523	H2ac20	3.774588941	up	7.79062408	4.016035134
Q9D0E1	Hnrnpm	3.767483985	up	7.78351912	4.016035134
P63323	Rps12	3.759163421	up	7.77519855	4.016035134
Q61696	Hspa1a	3.750734703	up	7.76676984	4.016035134
A0A0G2JF72	Csde1	3.585864744	up	7.60189988	4.016035134
Q8BX02	Kank2	3.570638305	up	7.58667344	4.016035134
Q9D024	Ccdc47	3.554854187	up	7.57088932	4.016035134
Q1HFZ0	Nsun2	3.53309656	up	7.54913169	4.016035134
A2AWT7	Ubtf	3.455826932	up	7.47186207	4.016035134
Q6IFX2	Krt42	3.447533533	up	7.46356867	4.016035134
O70503	Hsd17b12	3.432714332	up	7.44874947	4.016035134
Q9Z110	Aldh18a1	3.402928393	up	7.41896353	4.016035134

A0A1B0GR19	Rcn3	3.358651263	up	7.3746864	4.016035134
Q6PFA2	Clta	3.356891914	up	7.37292705	4.016035134
P63271	Supt4h1a	3.355124565	up	7.3711597	4.016035134
E9QA50	Ifrd1	3.336754703	up	7.35278984	4.016035134
A0A0A0MQM2	Plch1	3.309517631	up	7.32555277	4.016035134
Q91VB8	Hba-a1	3.306809081	up	7.32284422	4.016035134
Q6ZQ38	Cand1	3.293128981	up	7.30916412	4.016035134
P80316	Cct5	3.287243319	up	7.30327845	4.016035134
A0A0G2JG10	Dhx15	3.282187728	up	7.29822286	4.016035134
A0A140LI98	Uqcrc2	3.275455337	up	7.29149047	4.016035134
D3YZJ1	Sqstm1	3.258015937	up	7.27405107	4.016035134
A0A2R8W6S4	Pacsin2	3.228761544	up	7.24479668	4.016035134
Q9D8L3	Ssr4	3.219148379	up	7.23518351	4.016035134
Q9R0I7	Ylpm1	3.16206824	up	7.17810337	4.016035134
F6ZQL0	Mtdh	3.117500771	up	7.13353591	4.016035134
Q00623	Apoa1	3.096860077	up	7.11289521	4.016035134
A0A087WQ14	Actr3	3.096277995	up	7.11231313	4.016035134
Q6P5B5	Fxr2	3.089008427	up	7.10504356	4.016035134
A0A0G2JDU0	Igkv8-34	3.087304987	up	7.10334012	4.016035134
Q9D823	Rpl37	2.973376371	up	6.98941151	4.016035134
P61620	Sec61a1	2.915647099	up	6.93168223	4.016035134
Q61048	Wbp4	2.892928138	up	6.90896327	4.016035134
A0A0R4J0Z1	Pdia4	2.208682463	up	9.50017294	7.291490472
P34022	Ranbp1	2.149226649	up	9.46042562	7.311198973
O08796	Eef2k	2.080534191	up	9.15431662	7.073782425
P46935	Nedd4	1.53452678	up	9.81668075	8.282153966
Q68FD5	Cltc	1.479129255	up	8.74338243	7.264253177
P68372	Tubb4b	1.404287877	up	10.0084828	8.60419496
Q8C2Q7	Hnrnph1	1.133161008	up	9.05289305	7.919732044
G3X956	Supt16	1.046948886	up	8.89161797	7.844669084
Q52KR3	Prune2	1.015647044	up	9.12917088	8.113523835
