# Supplementary material for: AI662270/GRP94 axis couples the unfolded protein response to mitochondrial dynamics during acute myocardial infarction
Source: JCI Insight. 2025 Oct 8;10(19):e188904. doi: 10.1172/jci.insight.188904 (PMC12513489; doi:10.1172/jci.insight.188904)
Supplement: Unedited blot and gel images [file jciinsight-10-188904-s010.pdf]

Figure 1D

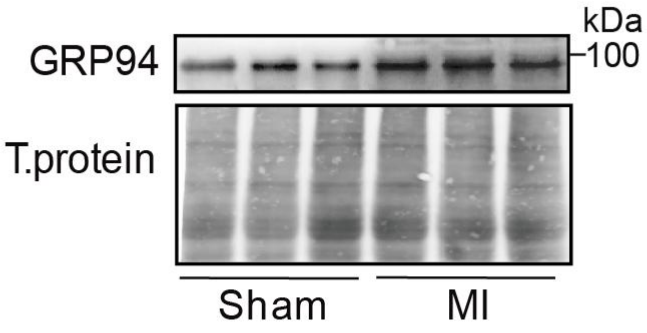

unedited blot

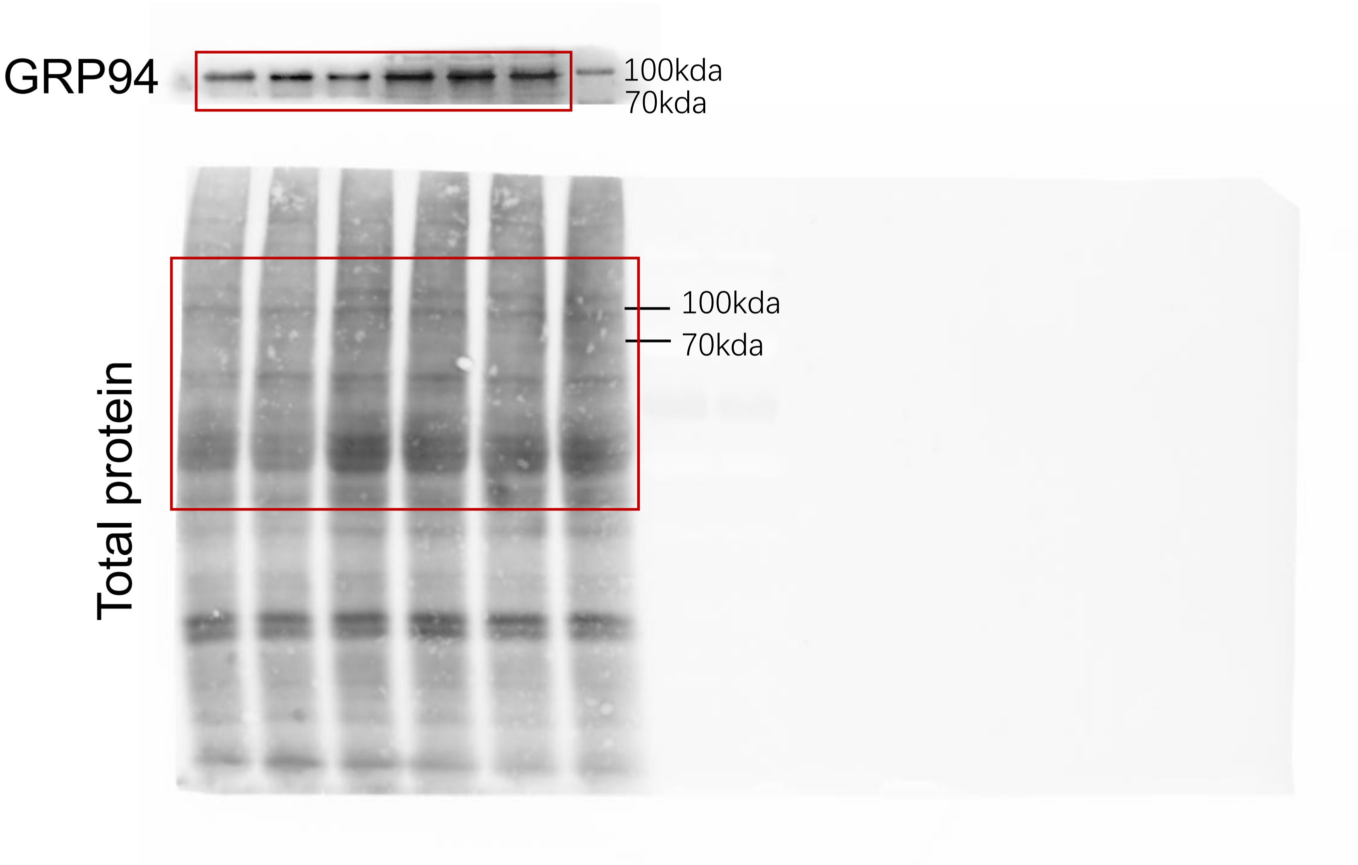

Figure 2A

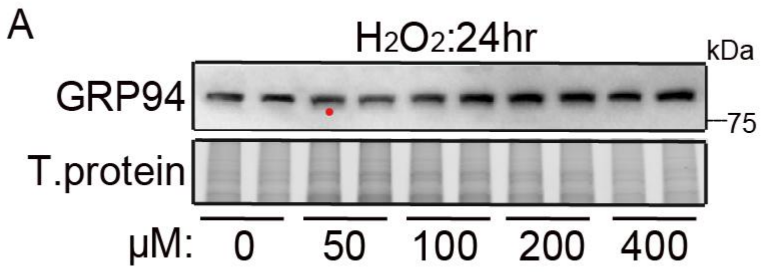

unedited blot

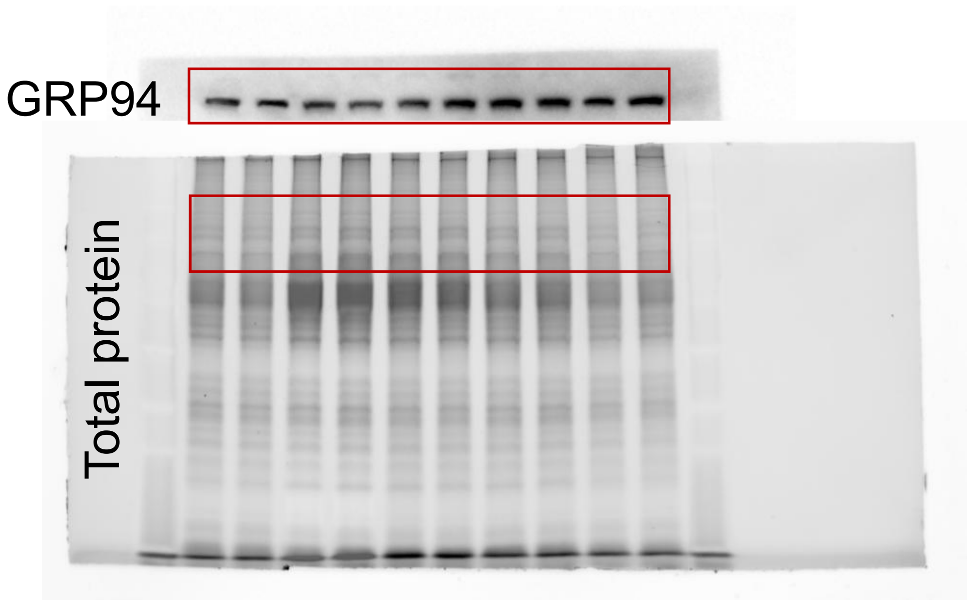

Figure 2B

unedited blot

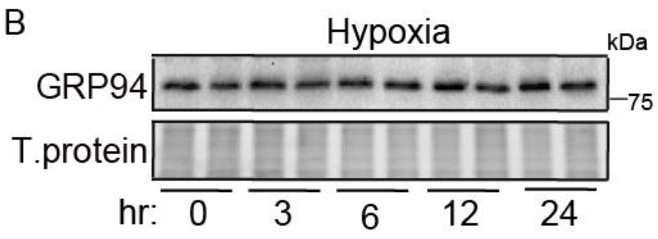

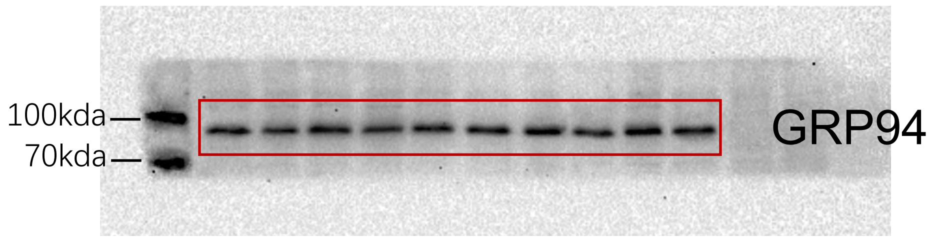

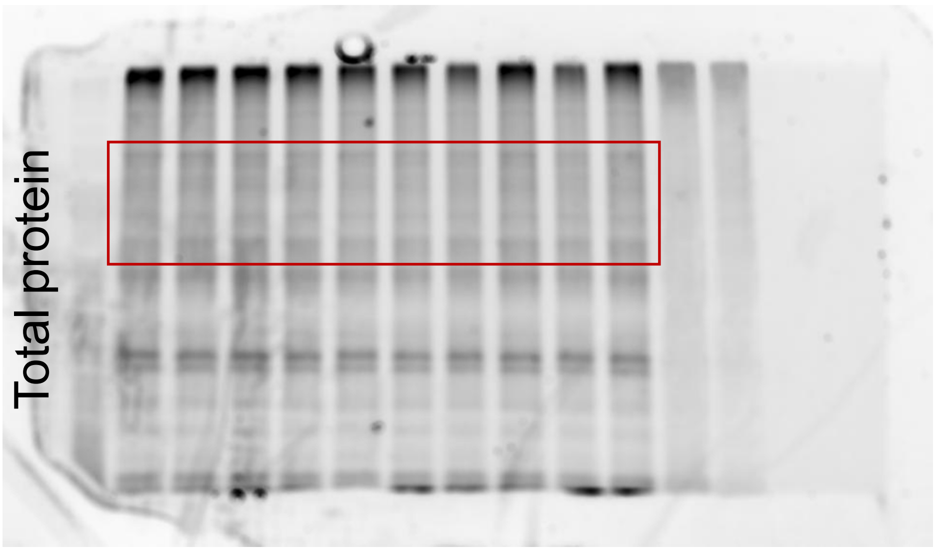

Figure 2E

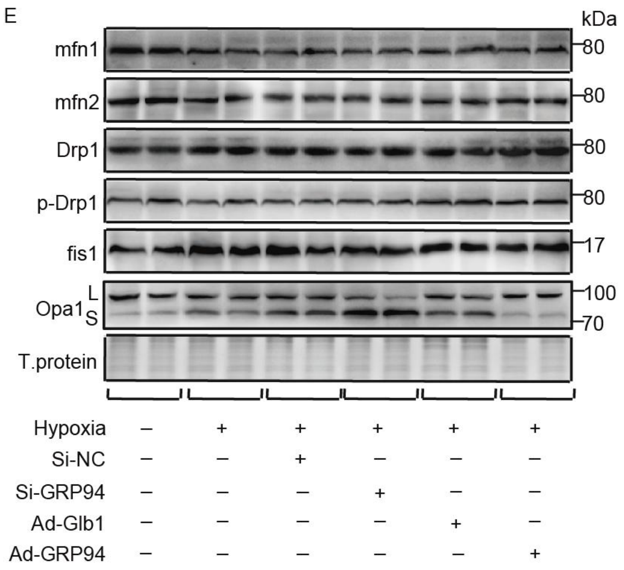

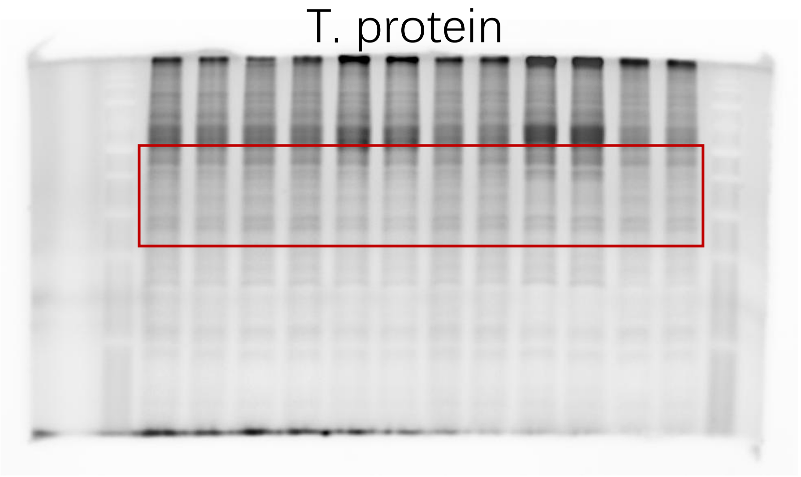

unedited blot

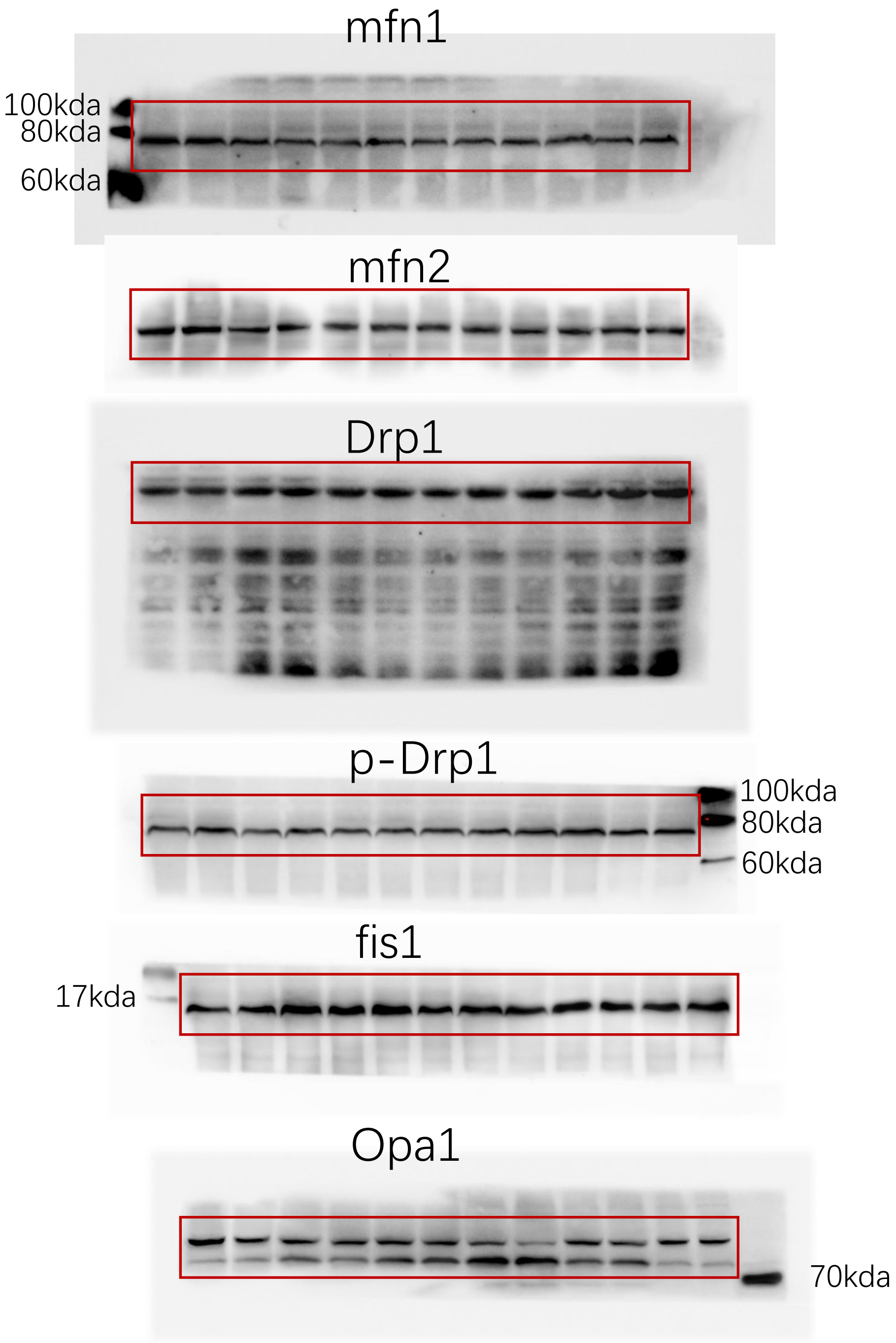

Figure 3B

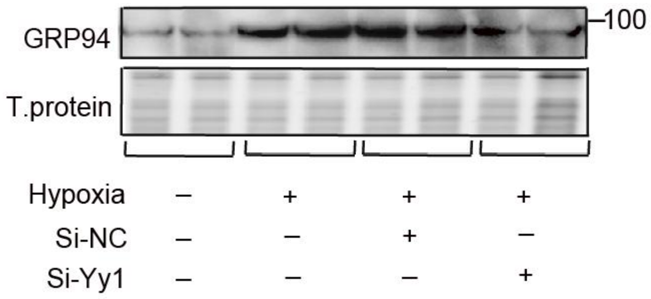

unedited blot

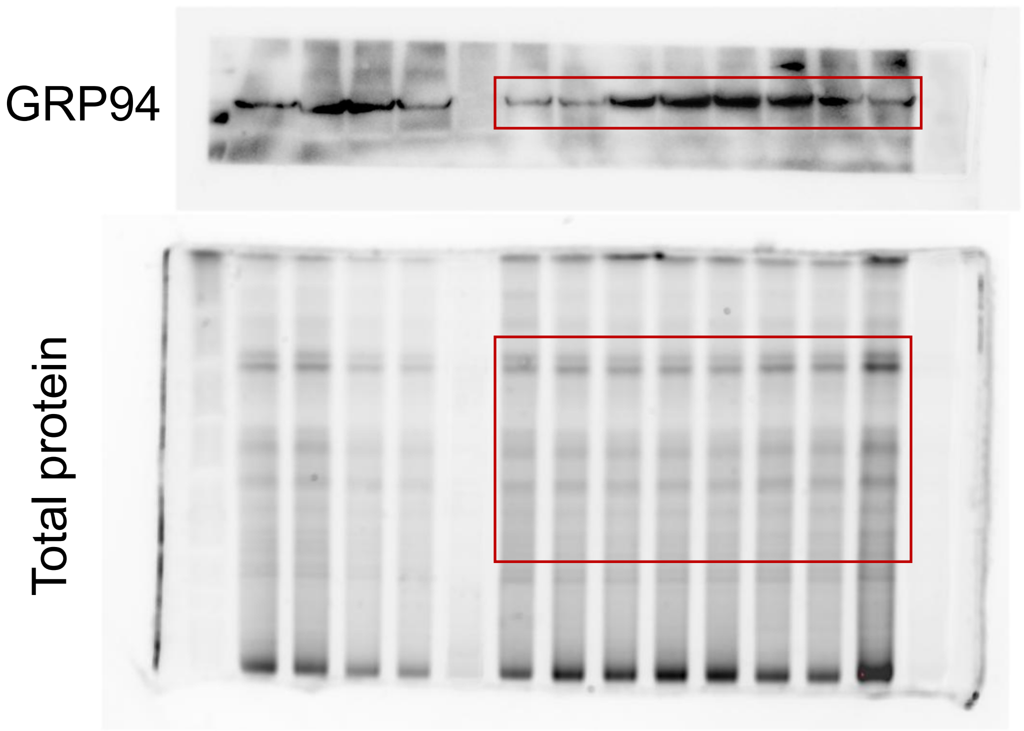

Figure 3C

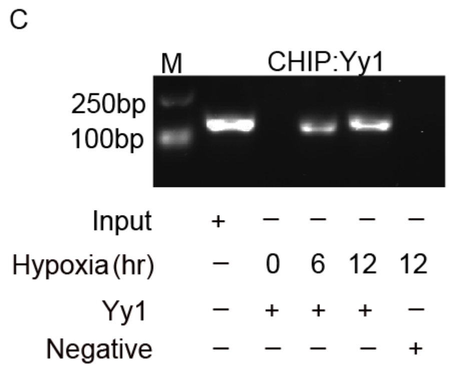
C
M
CHIP:Yy1
250bp
100bp
Input + – – – –
Hypoxia (hr) – 0 6 12 12
Yy1 – + + + –
Negative – – – – +

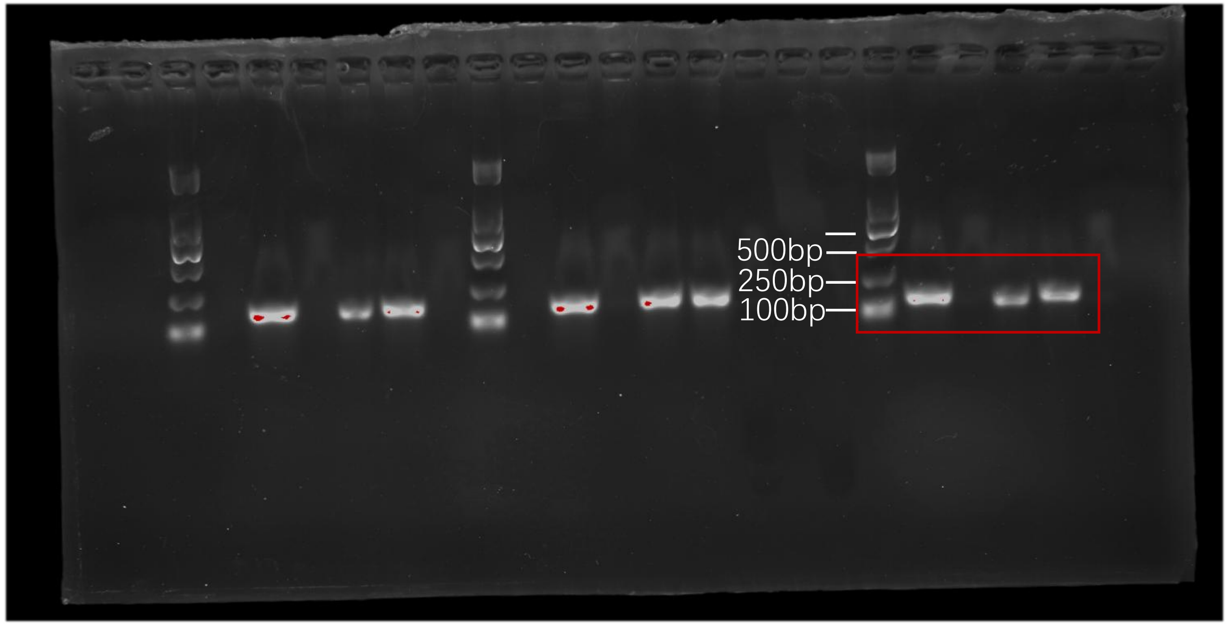
500bp
250bp
100bp

Figure 3D

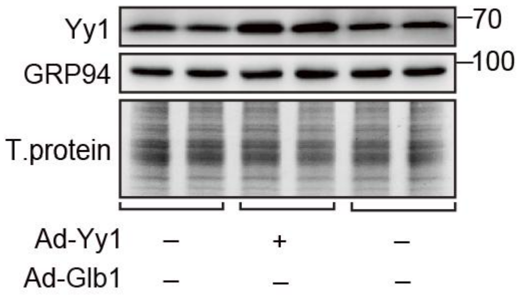

unedited blot

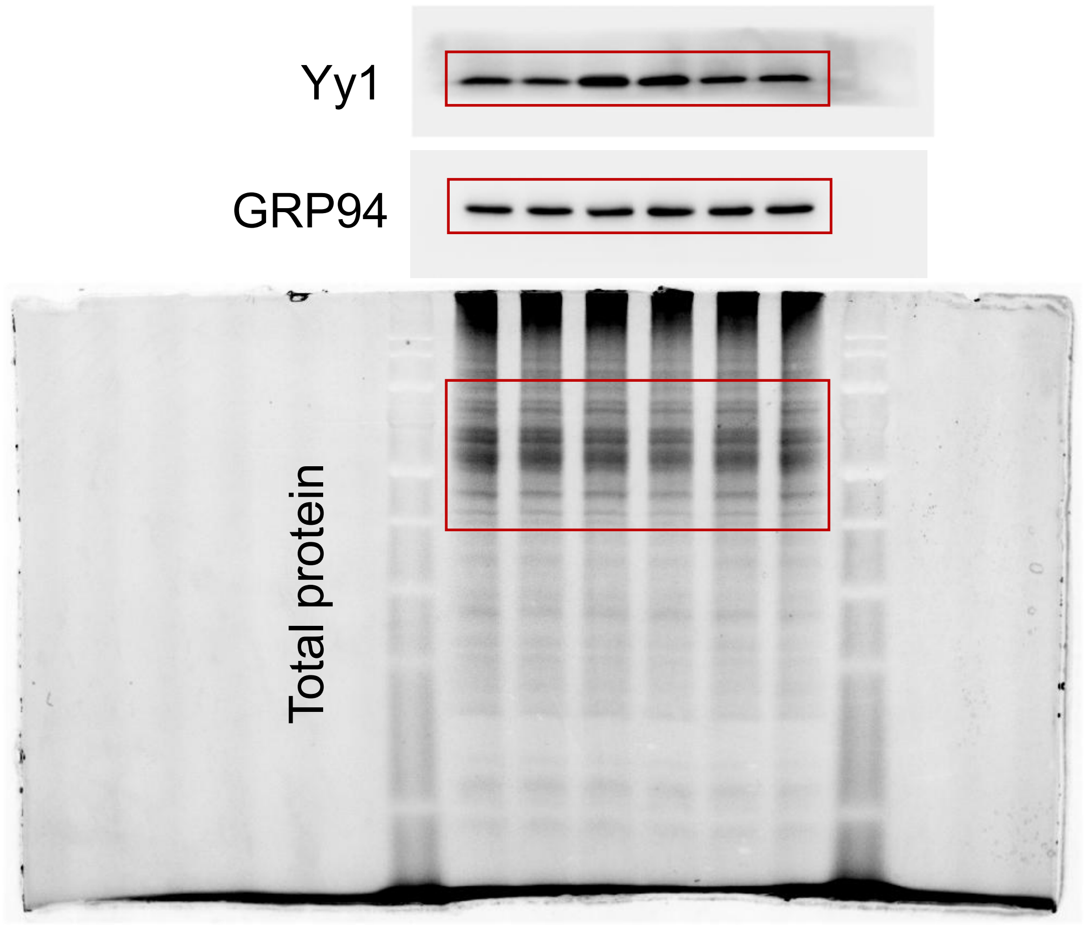

Figure 3E

unedited blot

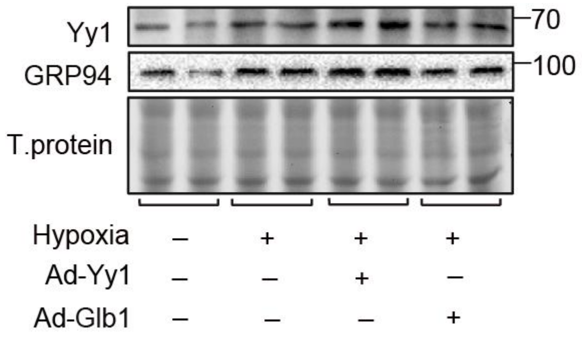

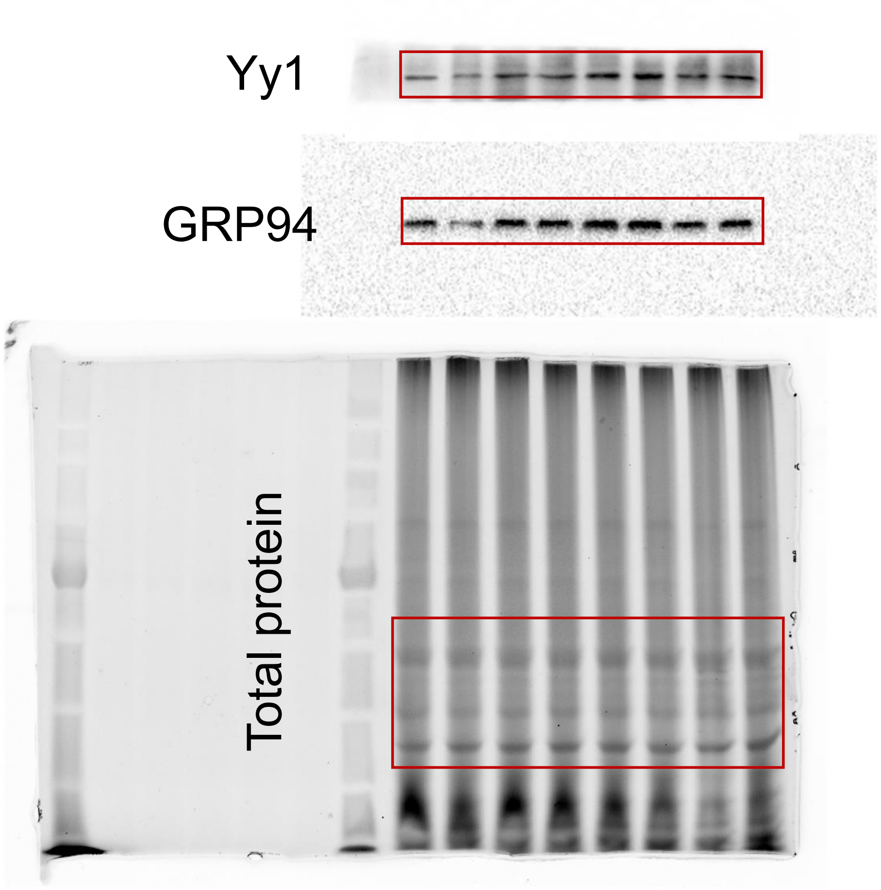

Figure 4C

unedited blot

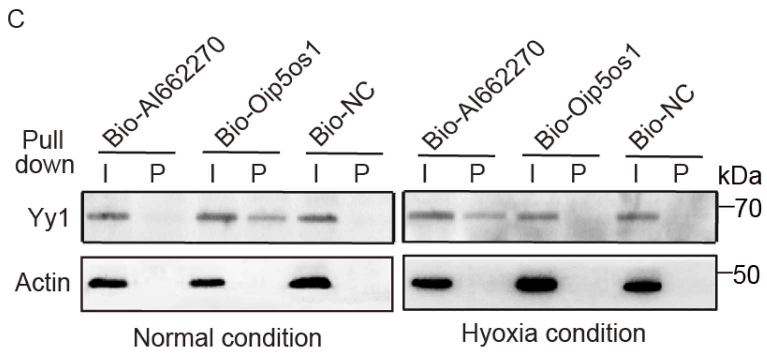

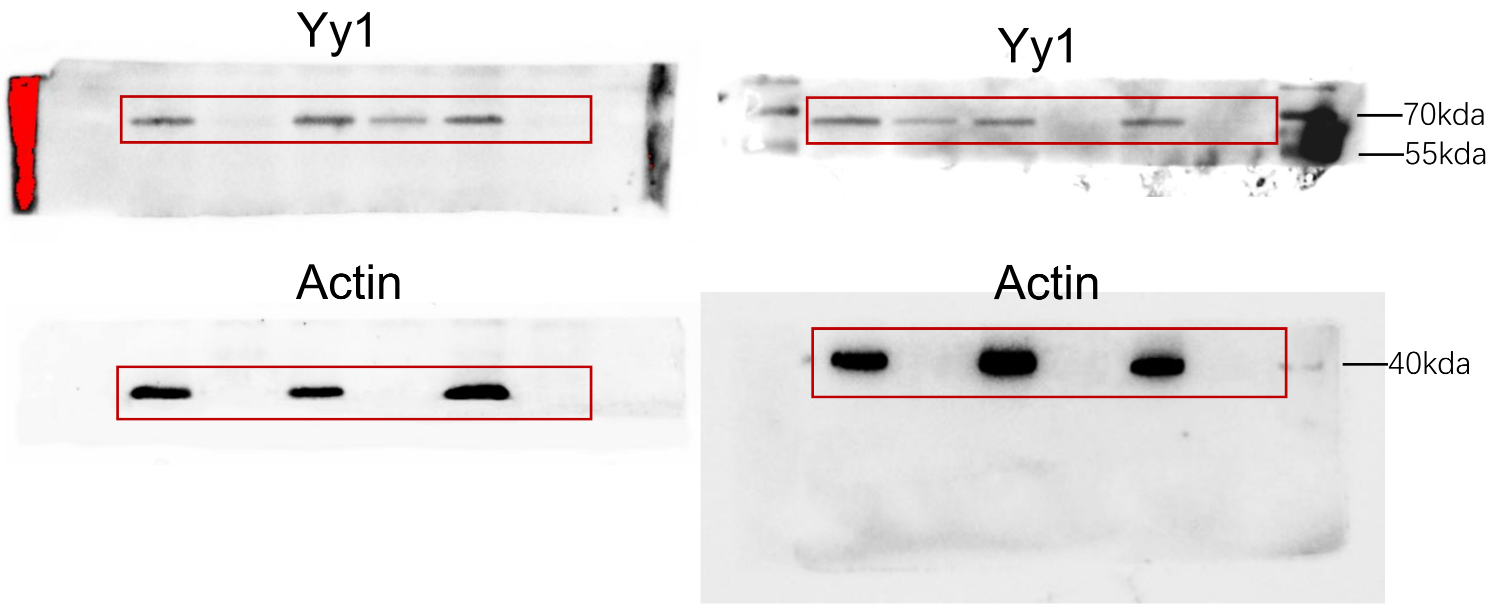

Figure 4F

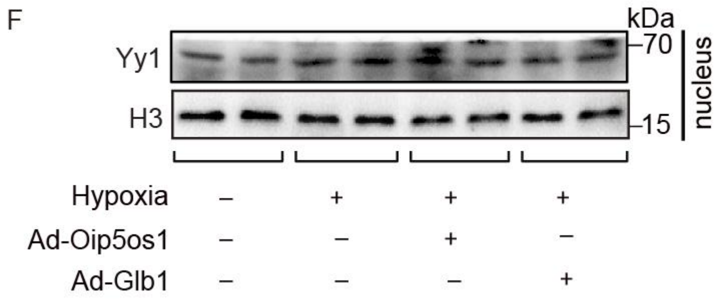

unedited blot

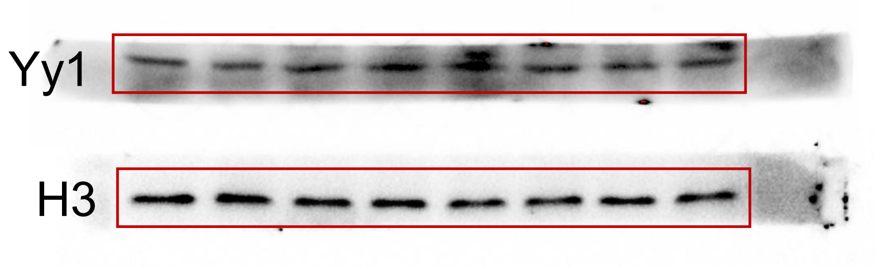

Figure 4G

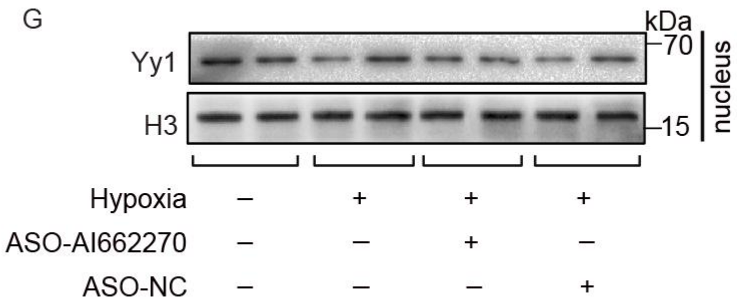

unedited blot

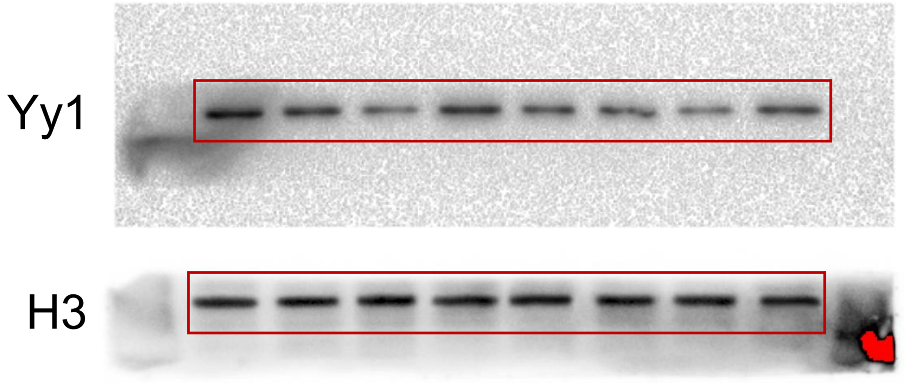

Figure 4H

unedited blot

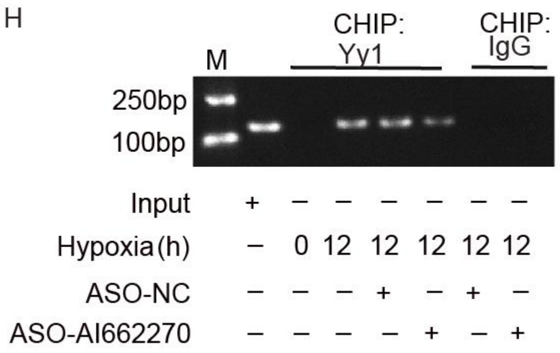

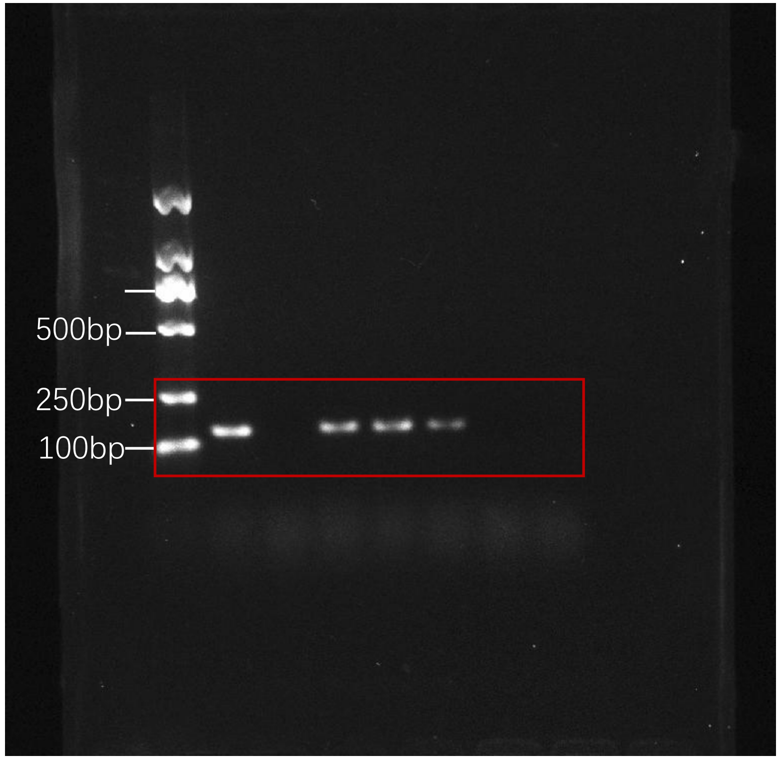

Figure 4I

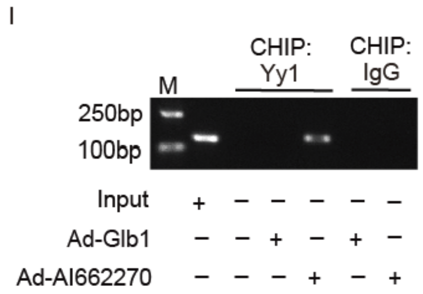

unedited blot

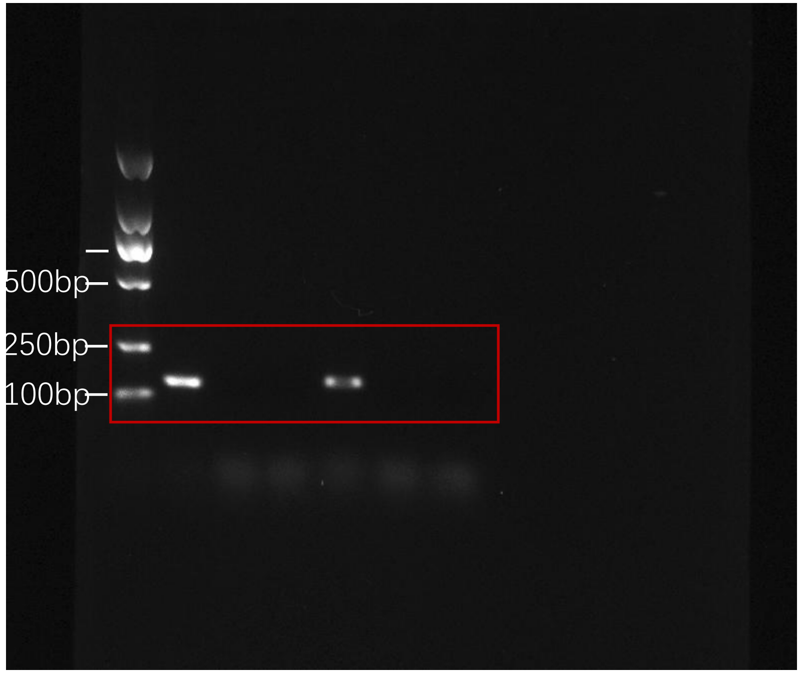

Figure 4J

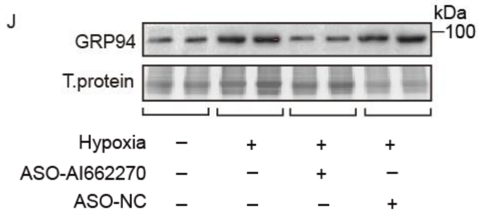

unedited blot

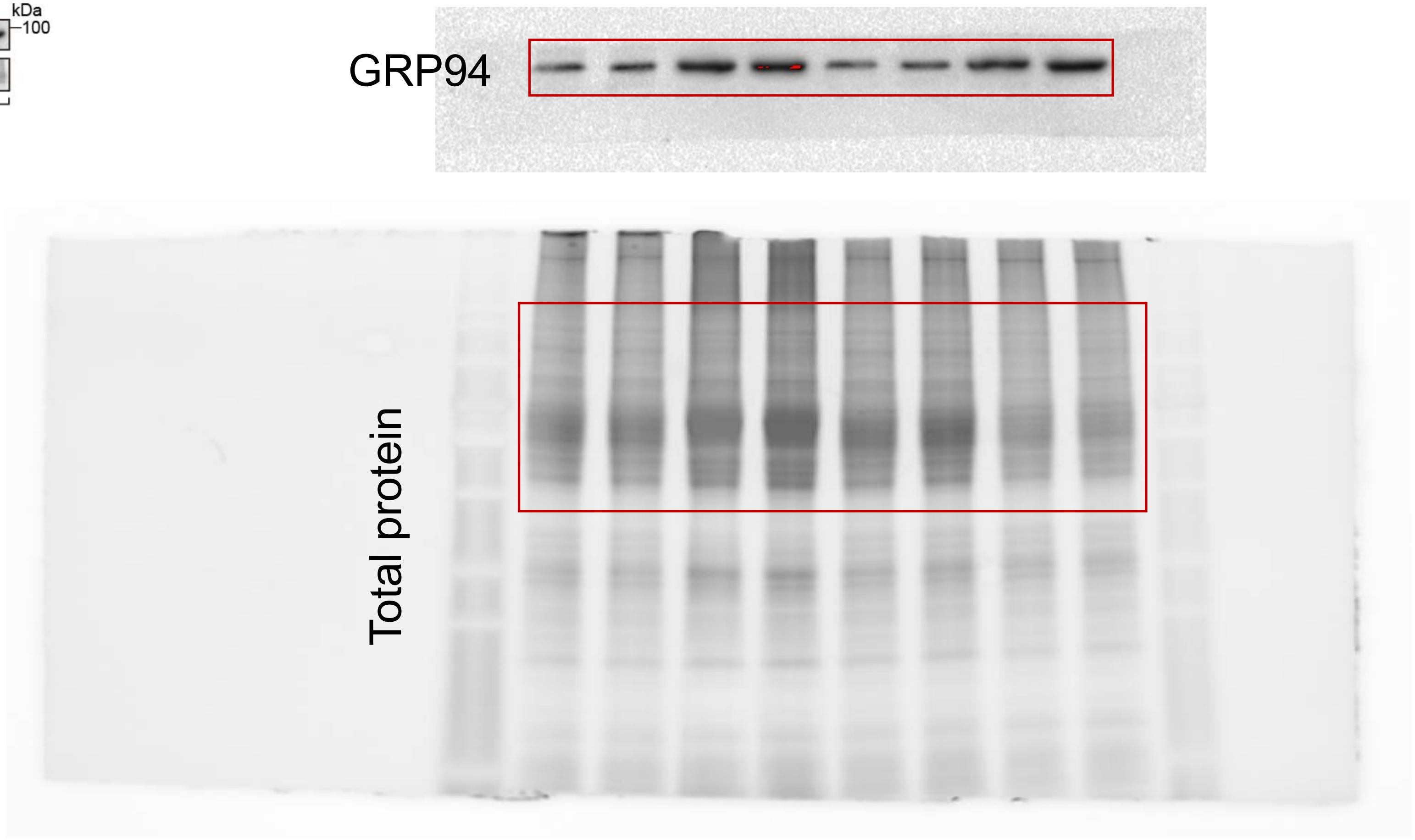

Figure 4K

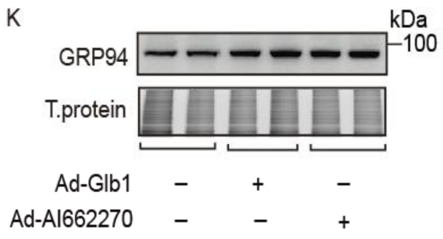

unedited blot

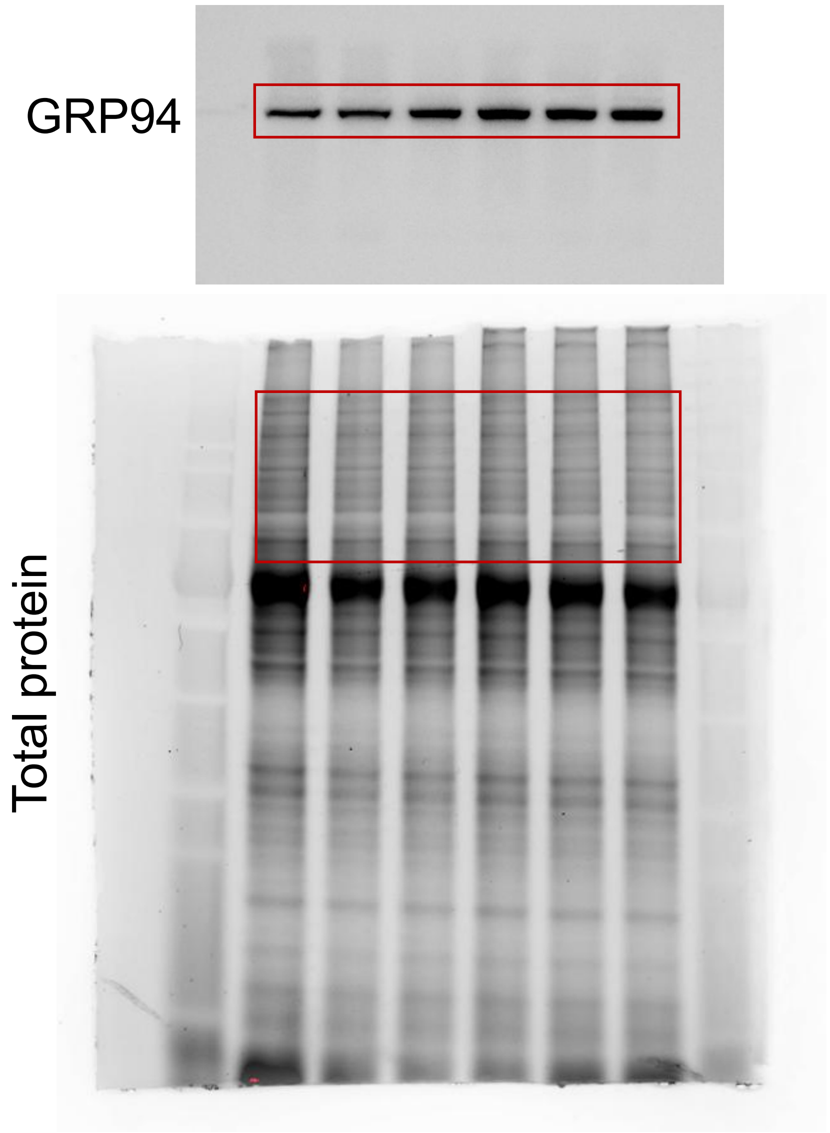

Figure 5D

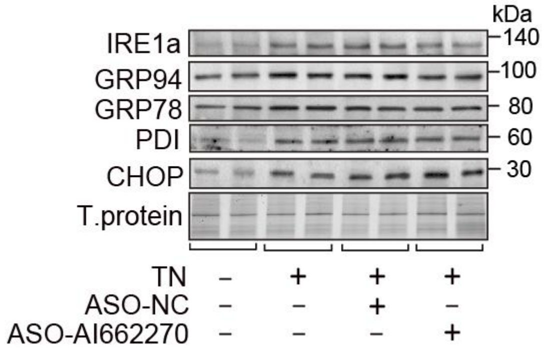

unedited blot

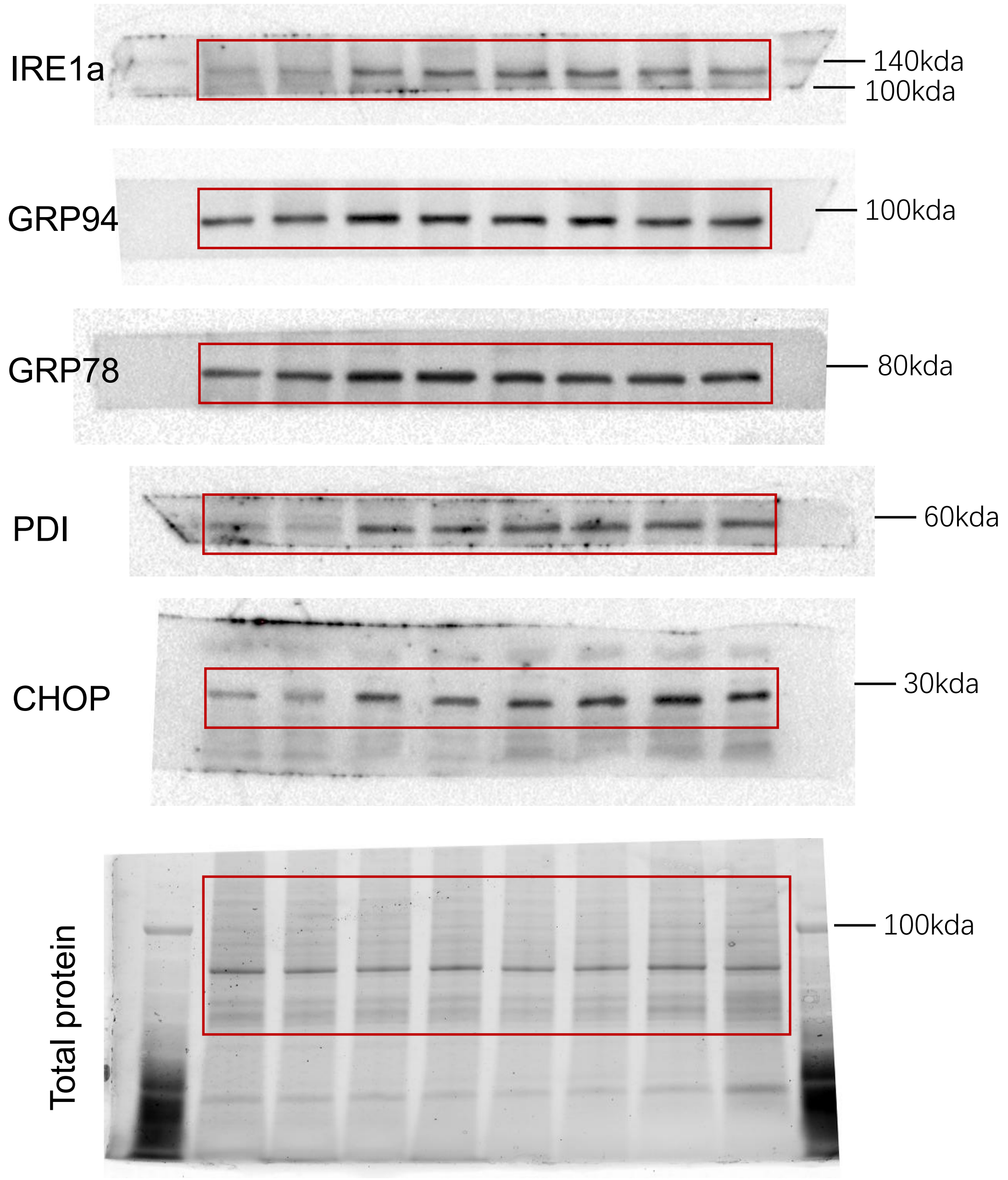

Figure 5E

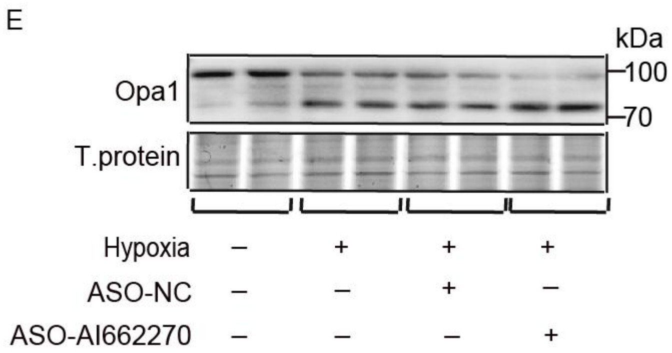

unedited blot

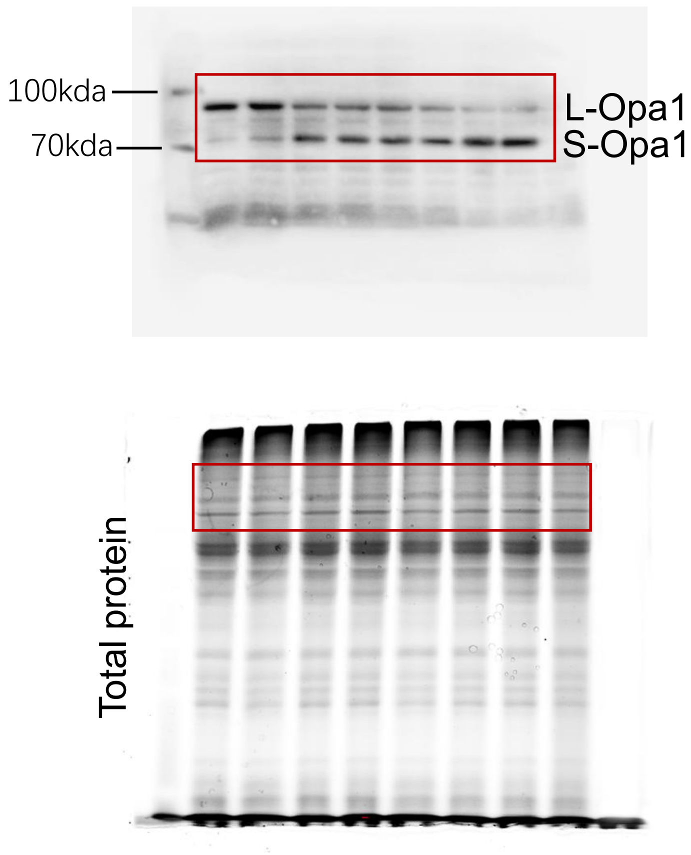

Figure 6A

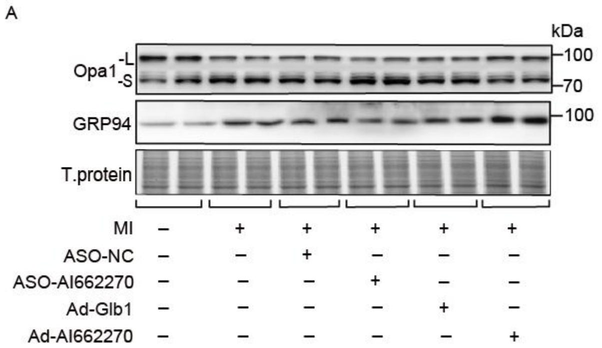

unedited blot

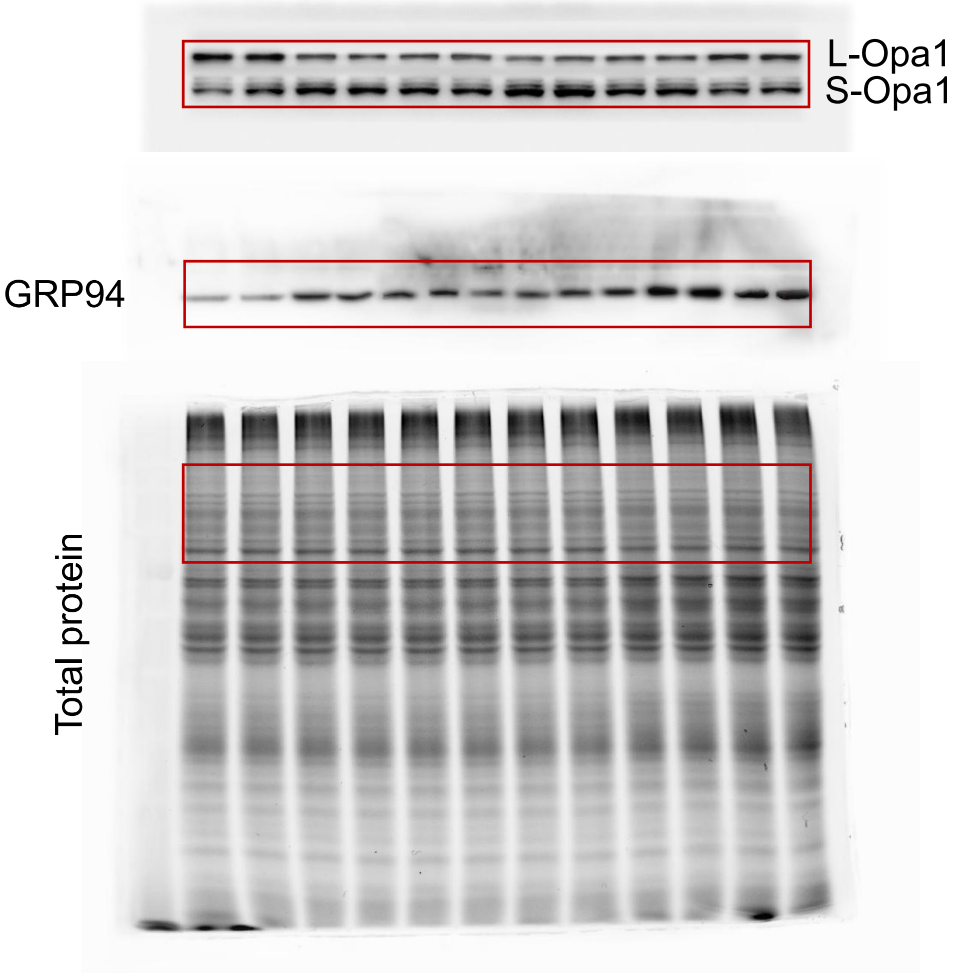

Figure 7C

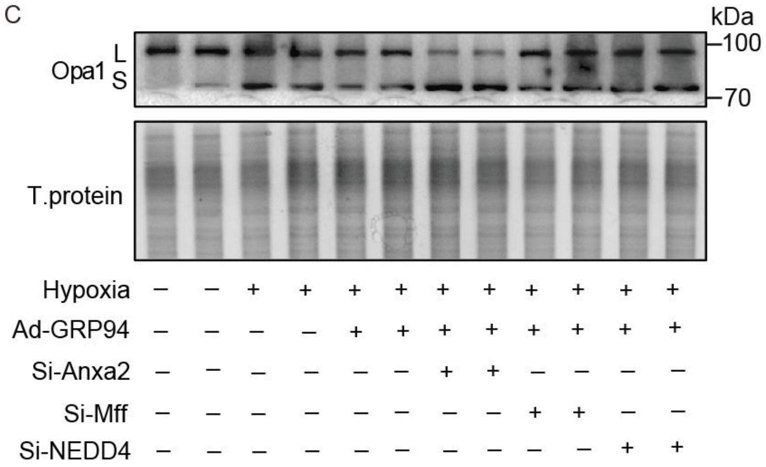

unedited blot

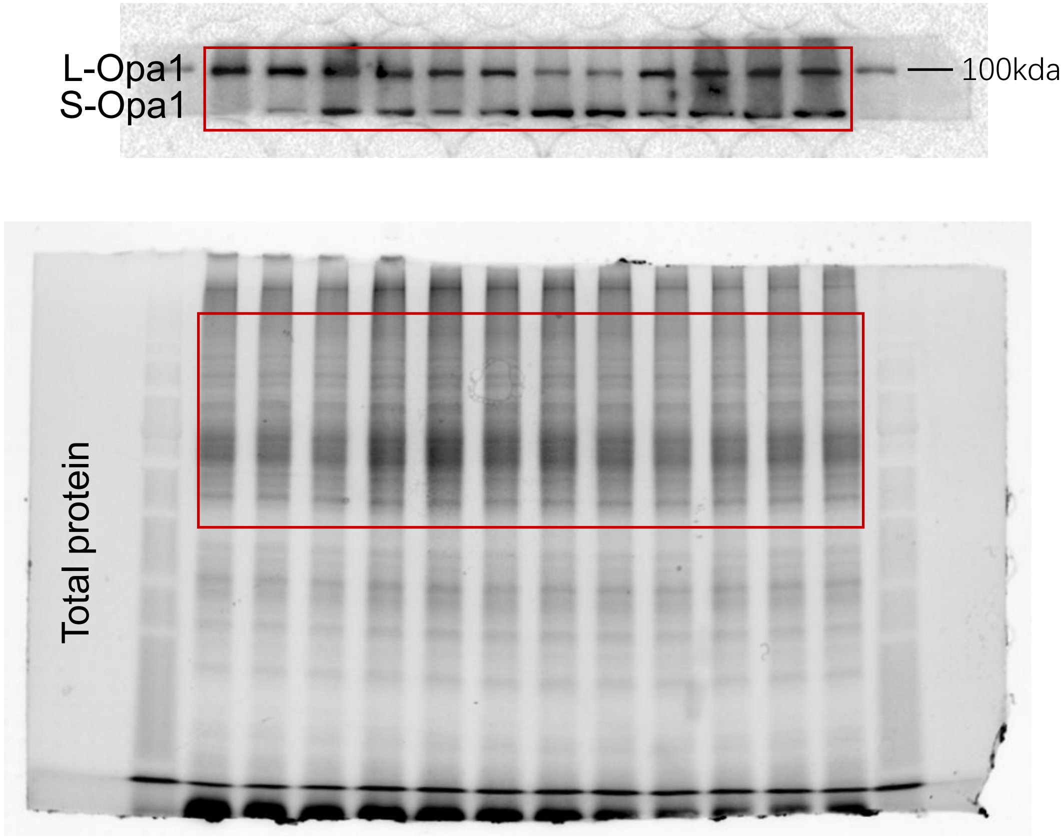

Figure 7D

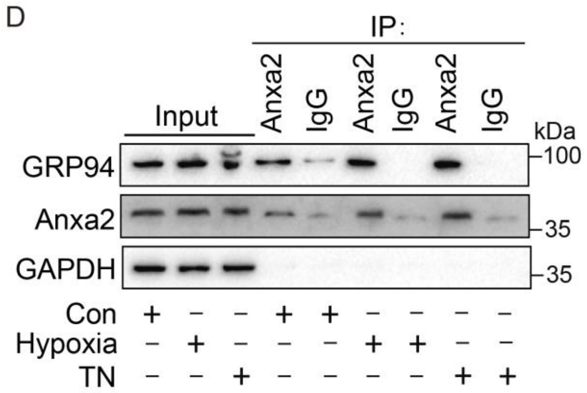

unedited blot

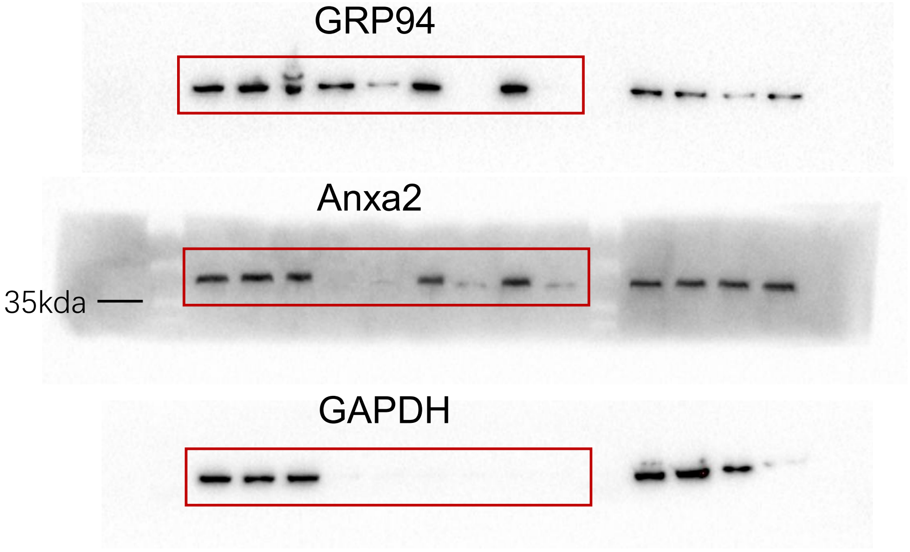

Figure 7E

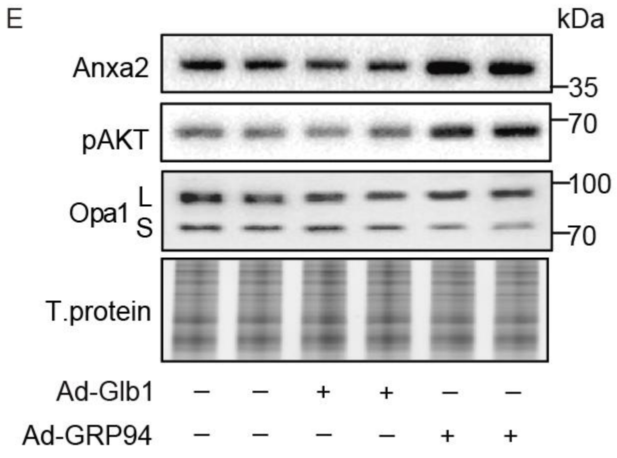

unedited blot

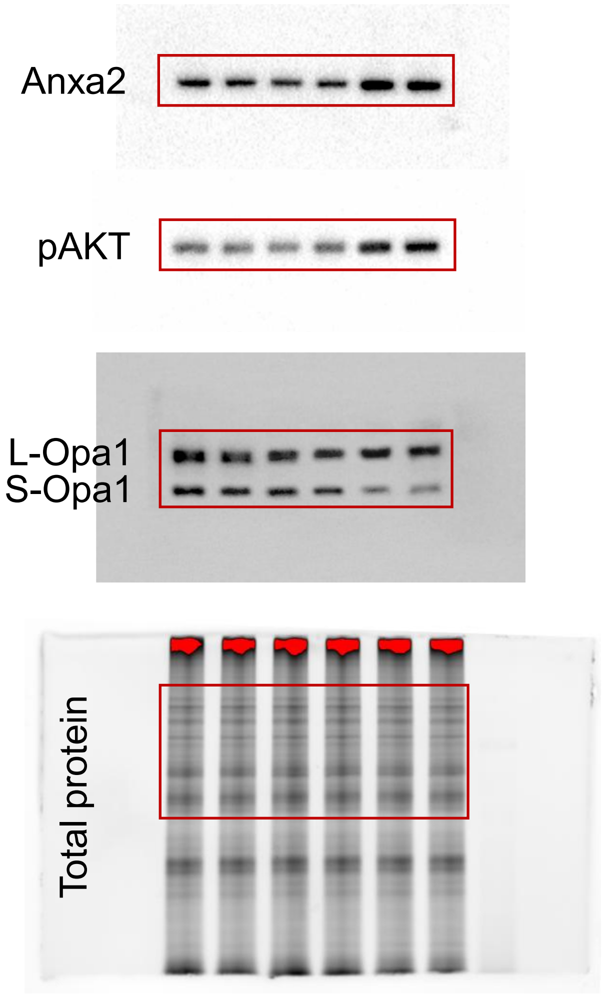

Figure 7F

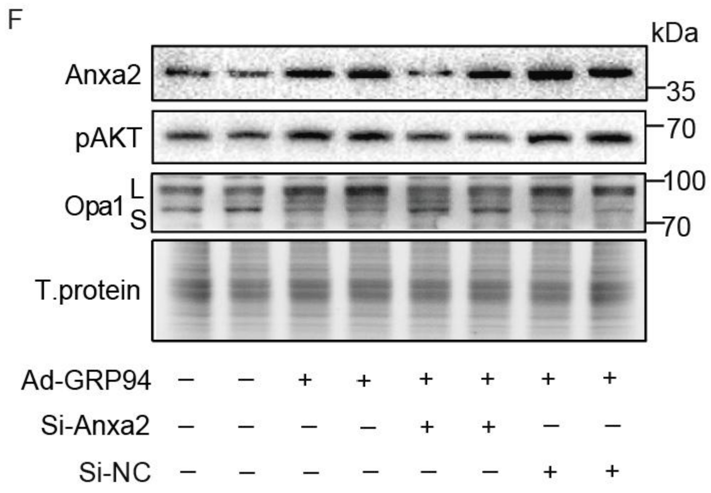

unedited blot

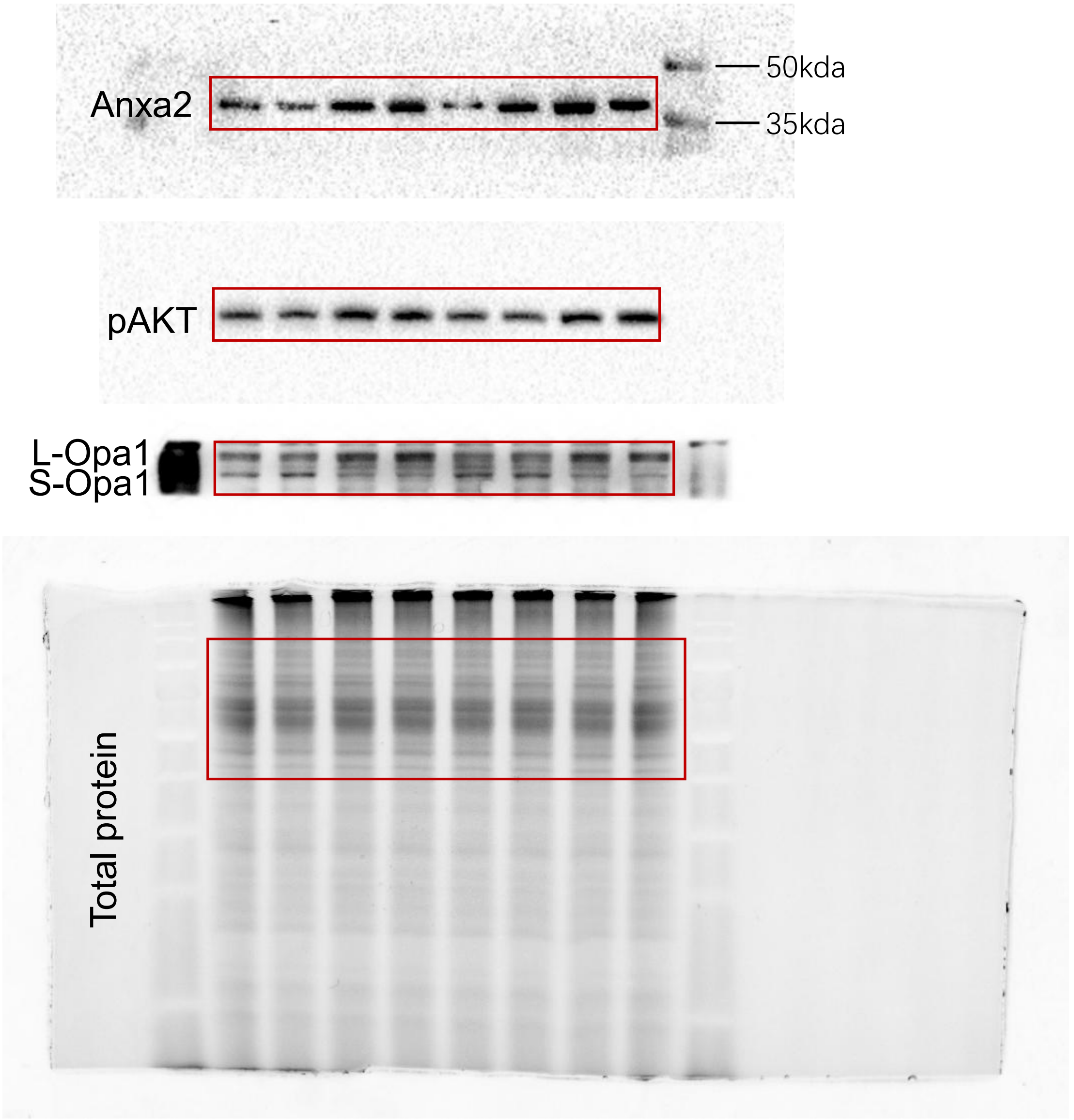

Figure 7G

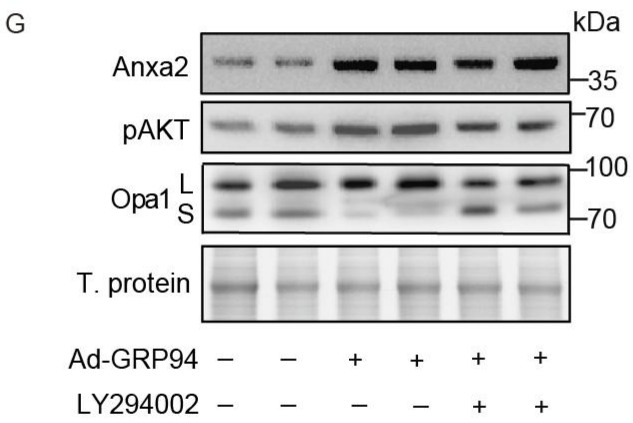

unedited blot

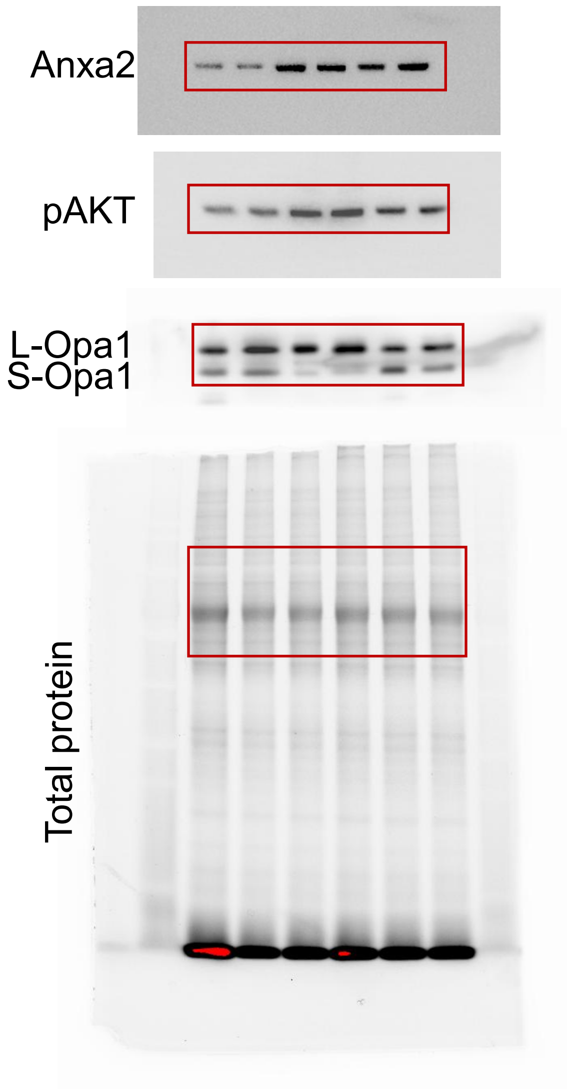

Figure S2A

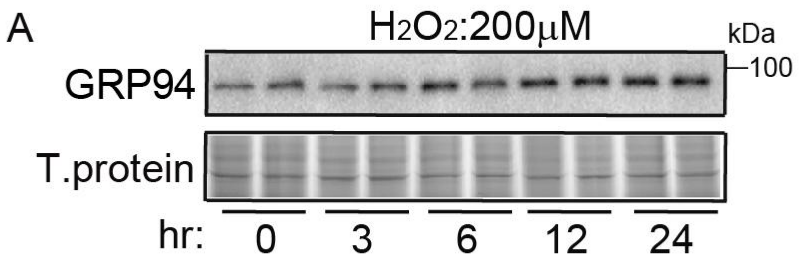

unedited blot

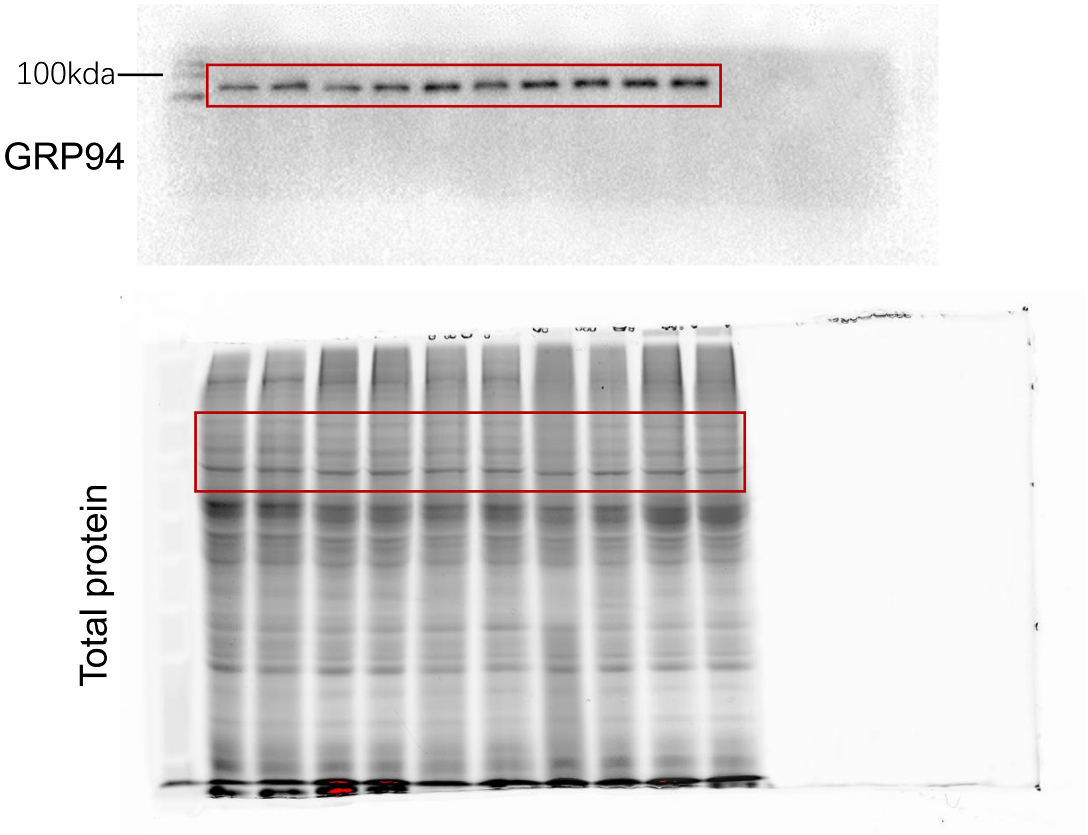

Figure S2B

unedited blot

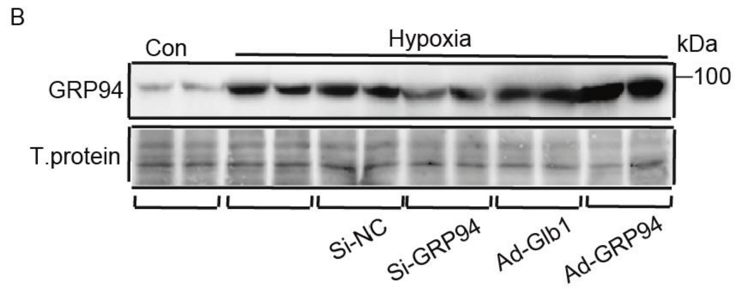

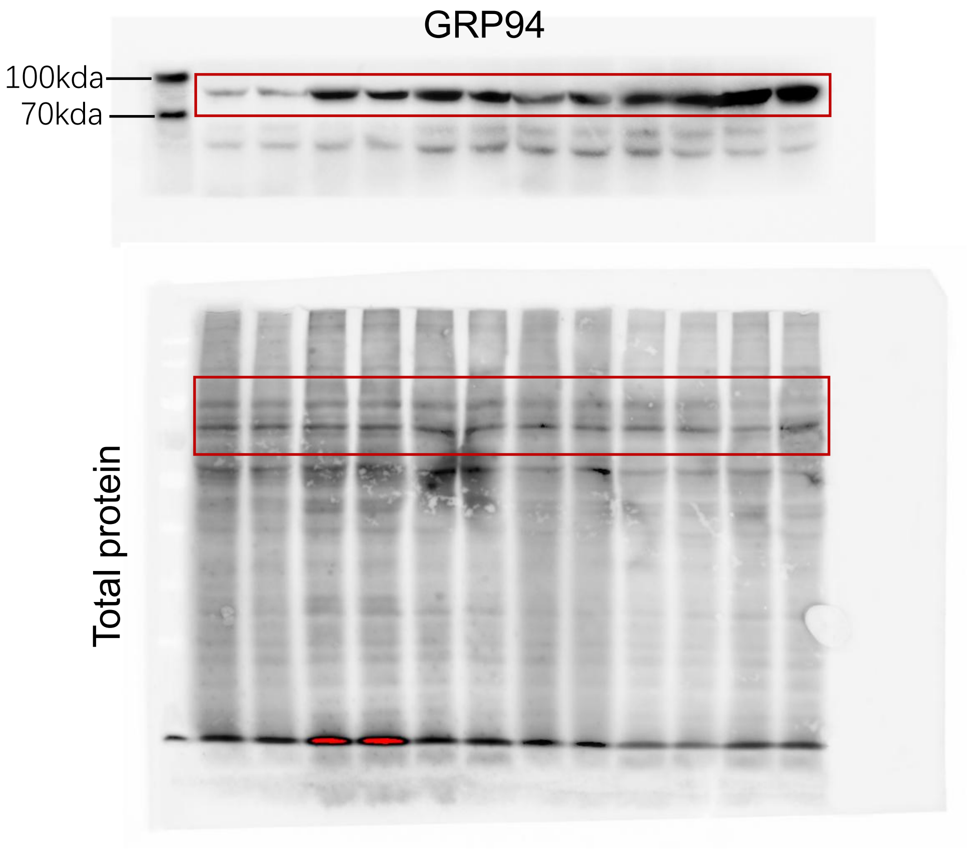

Figure S2I

unedited blot

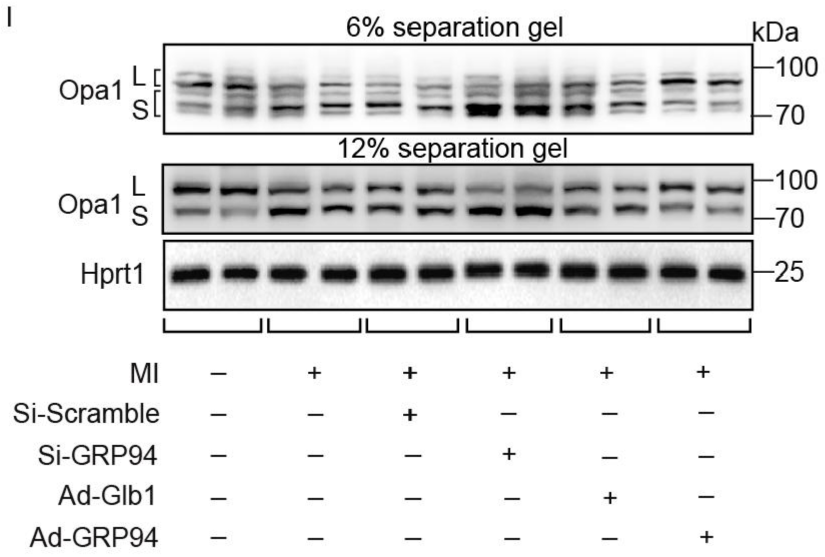

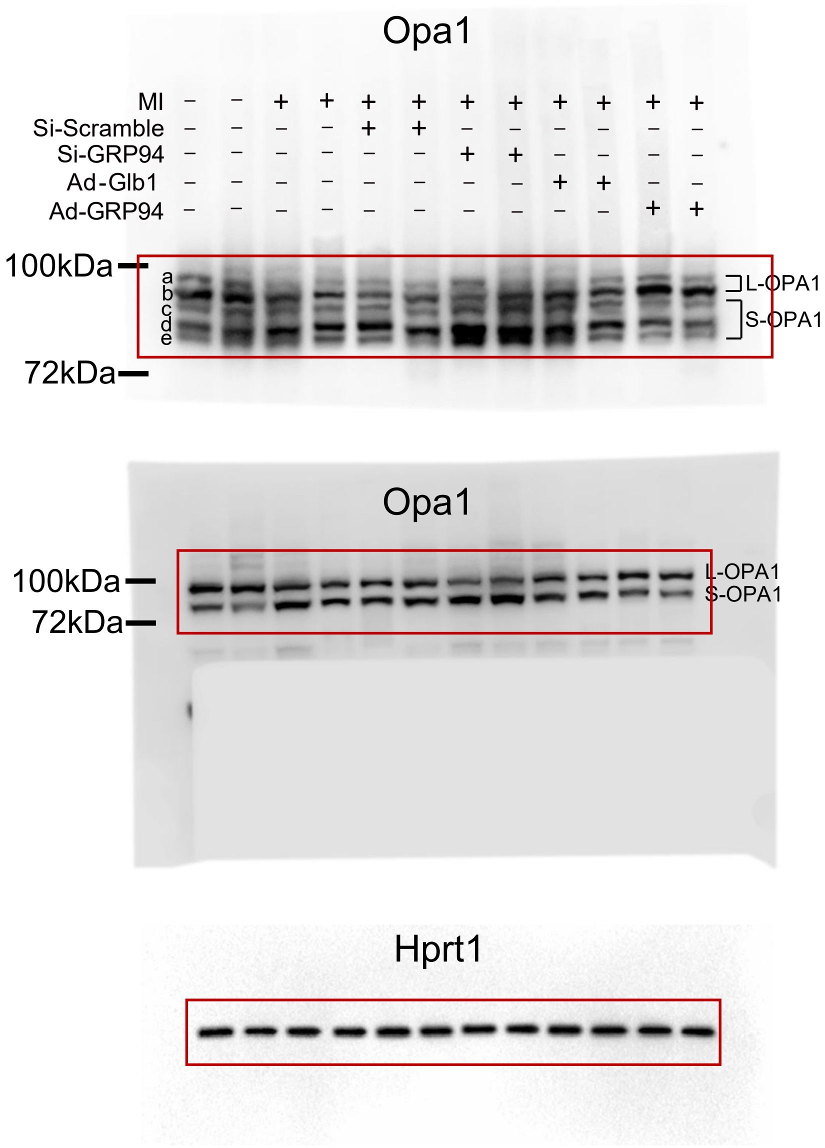

Figure S3D

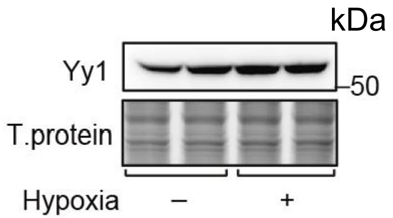

unedited blot

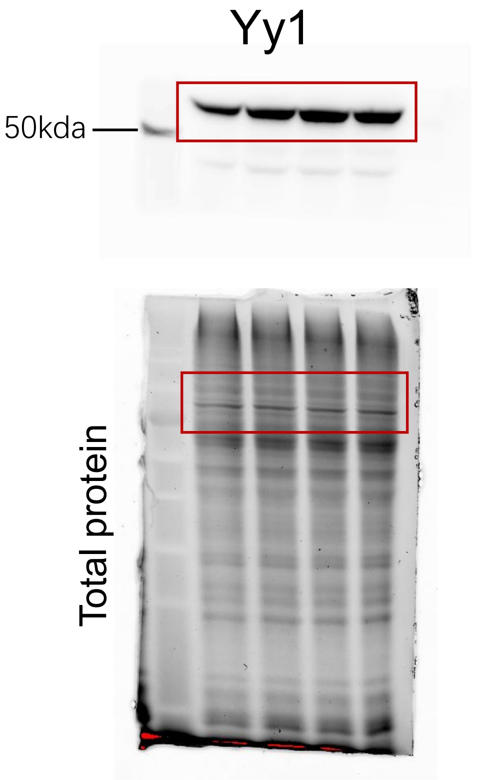

Figure S3E

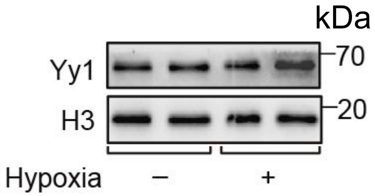

unedited blot

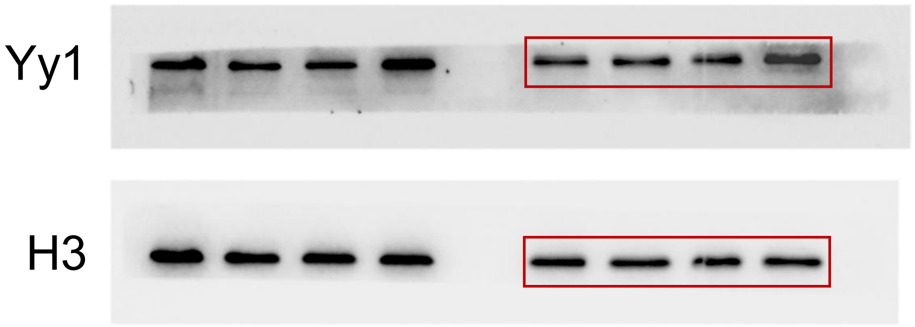

Figure S4F

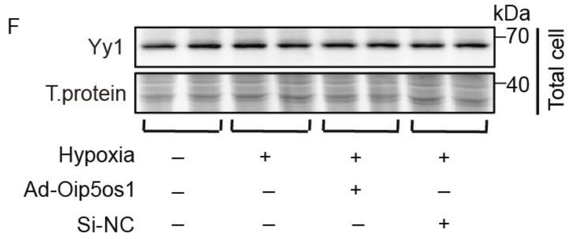

unedited blot

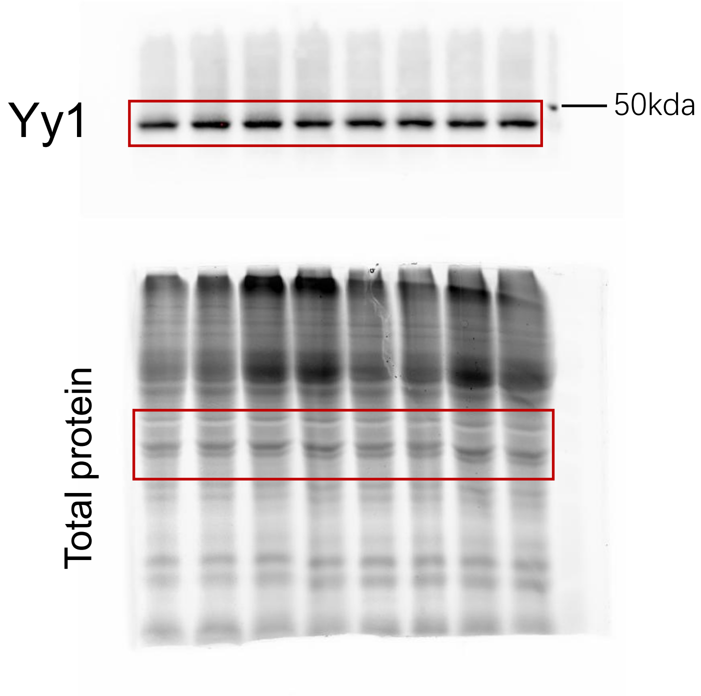

Figure S4G

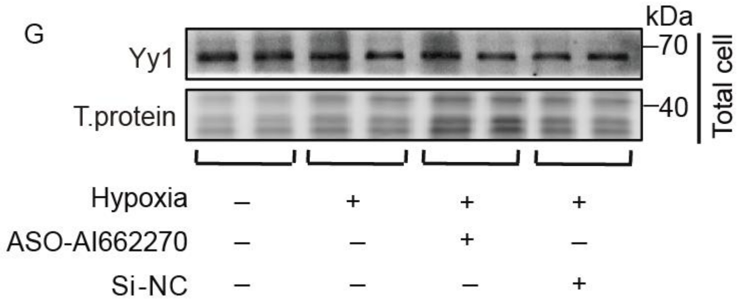

unedited blot

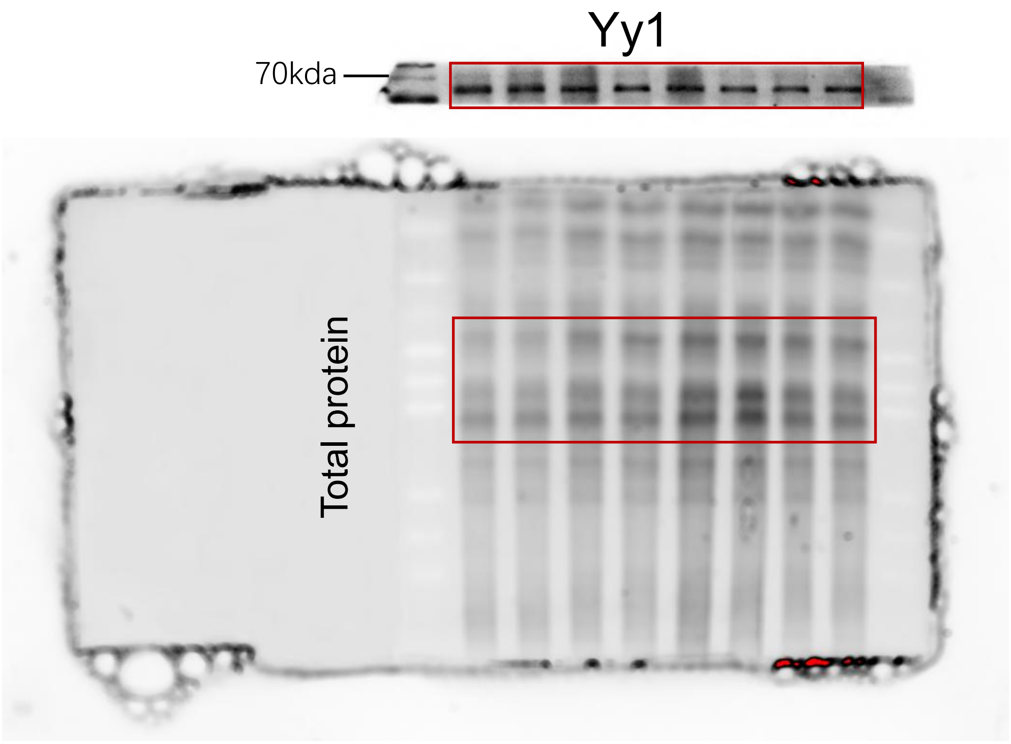

Figure S7B

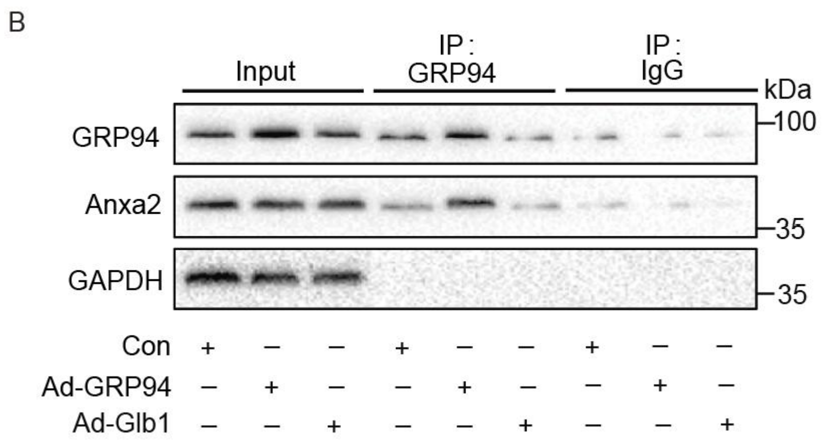

unedited blot

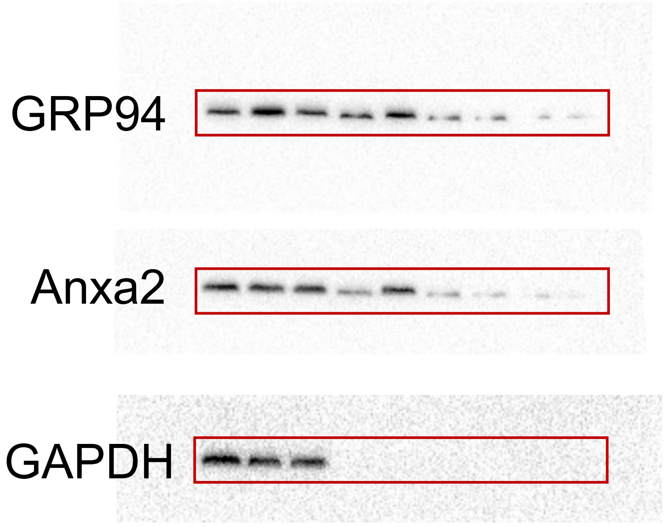

Figure S7H

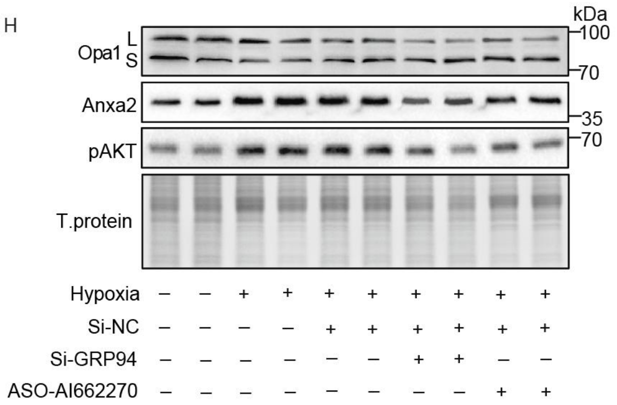

unedited blot

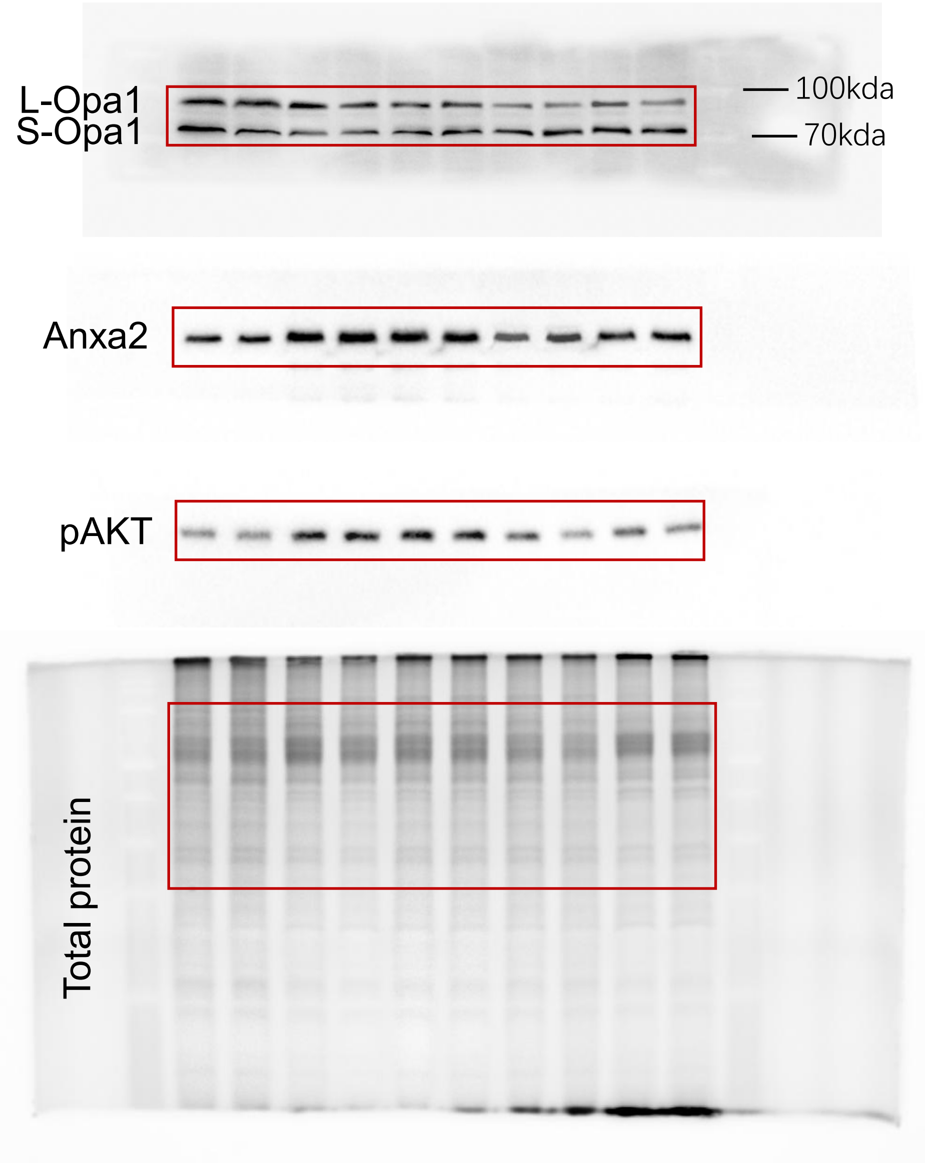

Figure S7I

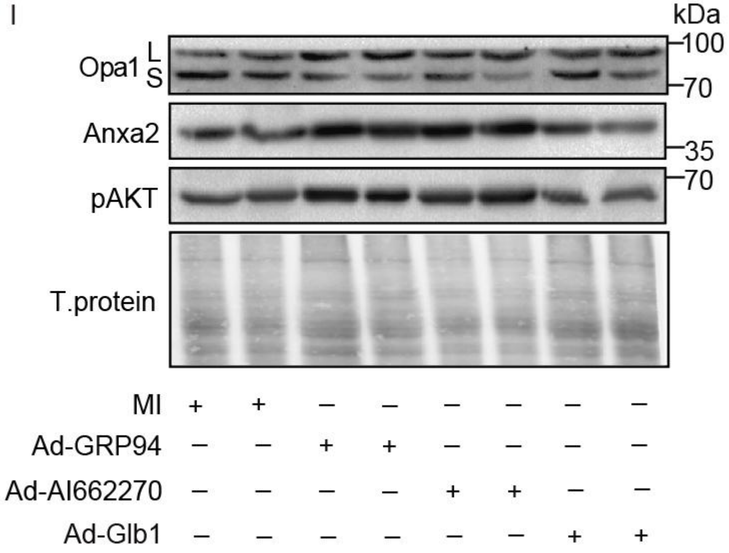

unedited blot

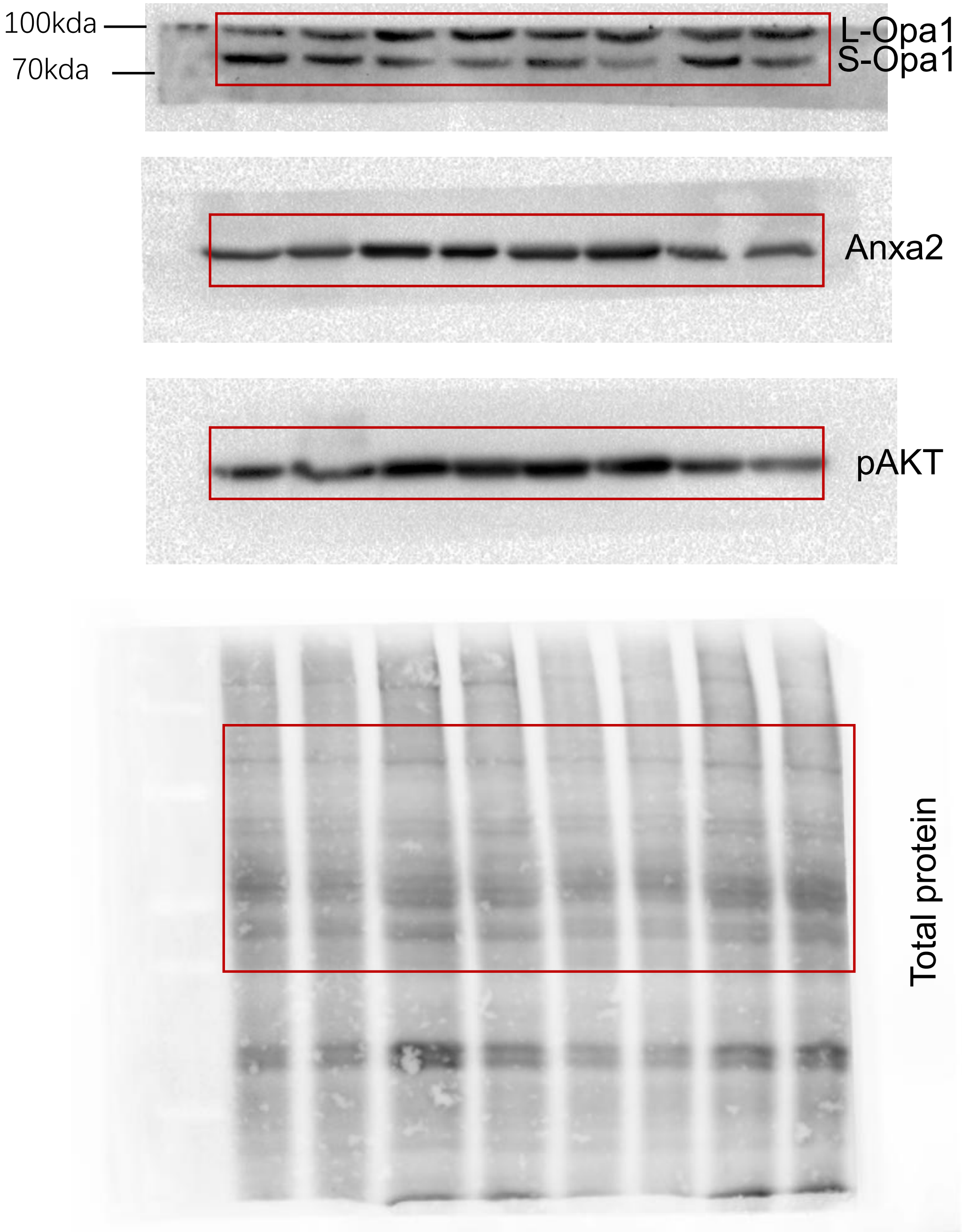

Figure S7J

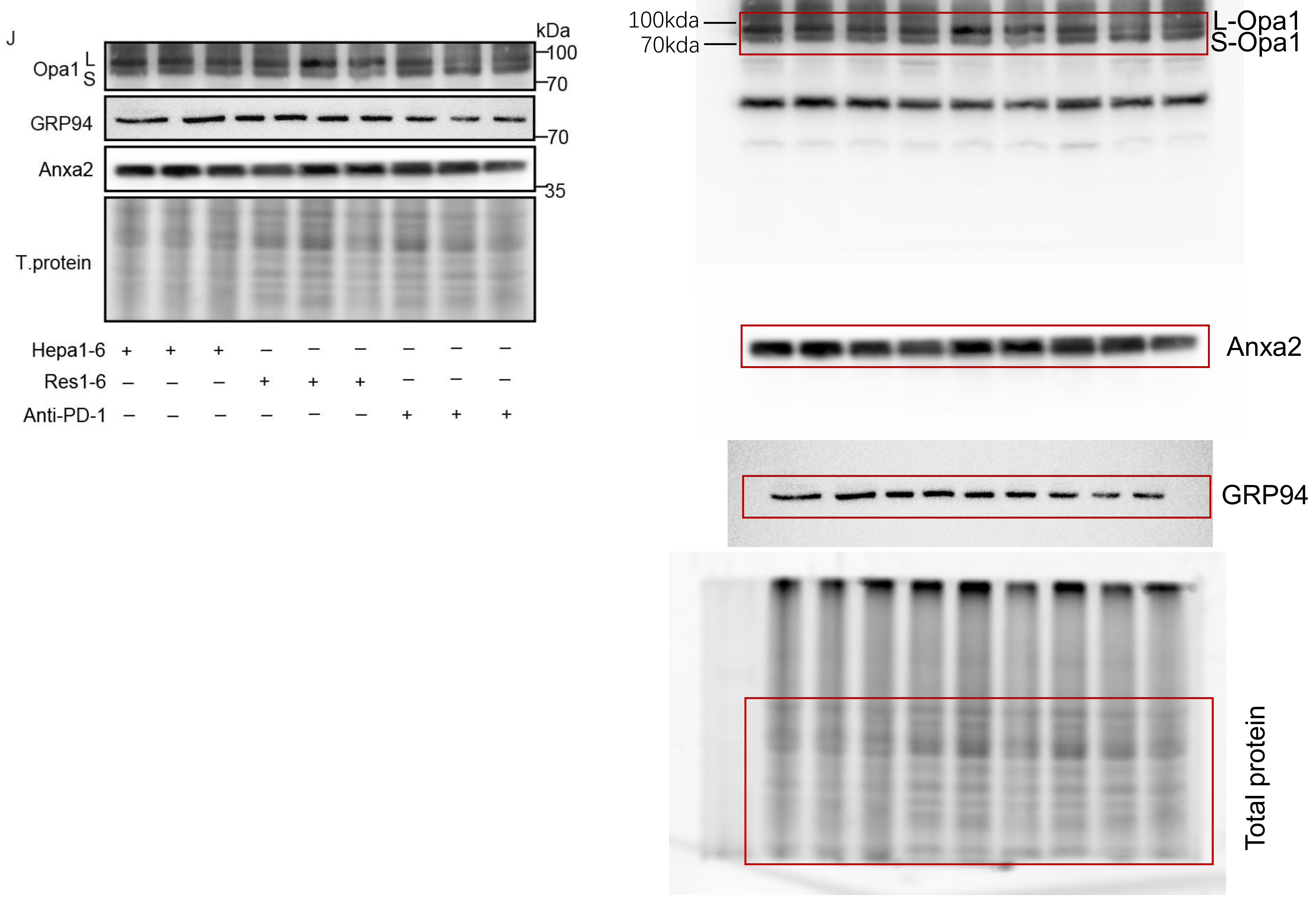

J
Opa1 L S
GRP94
Anxa2
T.protein
kDa
100
70
70
35
Hepa1-6 + + + – – – – – –
Res1-6 – – – + + + – – –
Anti-PD-1 – – – – – – + + +
unedited blot
100kda
70kda
L-Opa1
S-Opa1
Anxa2
GRP94
Total protein
